# Supplementary material for: Value and choice as separable and stable representations in orbitofrontal cortex
Source: Nat Commun. 2020 Jul 10;11:3466. doi: 10.1038/s41467-020-17058-y (PMC7351792; doi:10.1038/s41467-020-17058-y)
Supplement: Supplementary file 1 — Supplementary Information [file 41467_2020_17058_MOESM1_ESM.pdf]

Supplementary Information

Value and choice as separable and stable representations in orbitofrontal cortex

Daniel L. Kimmel, Gamaleldin F. Elsayed, John P. Cunningham, and William T. Newsome

June 2, 2020

Supplementary Discussion

On classifying stability

At short timescales, the distinction between stable and unstable representations can be subjective. For instance, we argued that the early benefit representation (Figure 5c,d) was stable for ~1 s, while another observer may describe it as a slowly evolving sequence. Going forward, developing objective tests to distinguish these cases will be critical, particularly given the prevalence of apparent sequences in the rodent[1–4]. Nonetheless, the extended stability of most representations (e.g., mid-trial benefit, choice, and expected reward) is unequivocally distinct from sequence-like encoding.

The type of stability we describe in OFC (i.e., consistent selectivity within the trial) contrasts other types of stability in the literature. For instance, selectivity may change within the trial, but, for a given time in the trial, remain consistent across experimental sessions[2,5]; or selectivity may be consistent between behavioral contexts, irrespective of temporal stability[6].

Supplementary Figures

Behavior

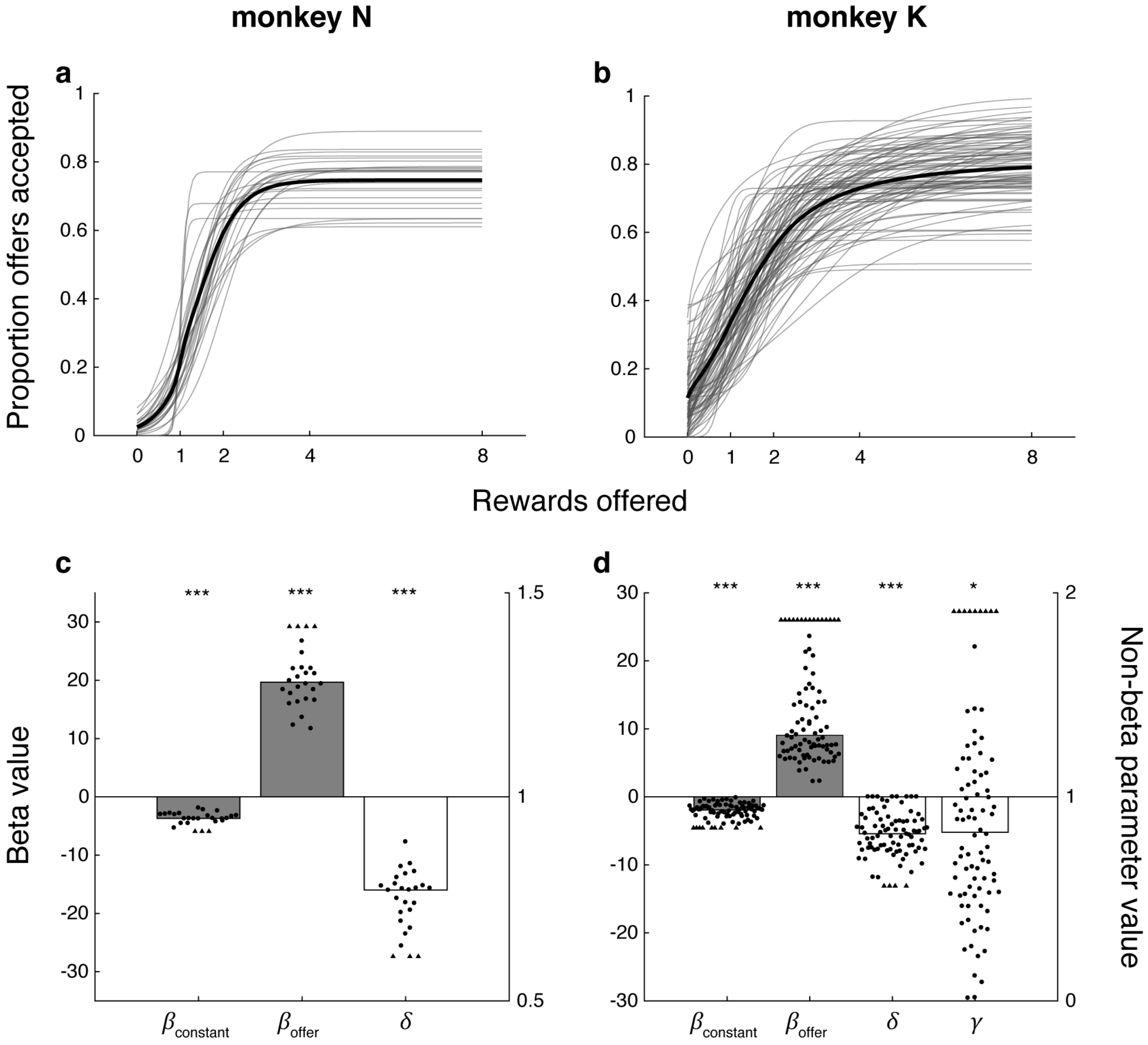

Supplementary Figure 1. Logistic regression of choice behavior.
(a,b) The logistic function (Equation (1)) fit to the choice behavior from individual sessions is plotted (thin gray curves) as a function of offer size for monkeys N (a) and K (b). The average curve across sessions (thick black curve) was computed as the mean of the individual curves for visualization only, and does not represent a separate logistic function fit to the aggregate data.

The smoothly increasing accept rate within individual sessions demonstrated that valuation of the offer varied from trial to trial, not merely between sessions (as demonstrated in Figure 1b). Alternatively, an abrupt step function would have suggested that animals applied a constant threshold for offer size, above which they accepted all offers.

(c,d) The logistic model included coefficients for offer size (β_{offer}) and a constant ($\beta_{constant}$) and additional parameters for a sub-unity saturation point (δ) and sublinear utility function (γ, monkey K only). For monkeys N (c) and K (d), bar height represents the median parameter value across N=26 (c) and N=86 (d) experimental sessions for beta (β; gray bars) and non-beta (open bars) parameters, which

reference the left and right ordinates, respectively. Parameter values from individual sessions are shown as circles with outliers (triangles) plotted not-to-scale (all values were included in median calculation and hypothesis testing). Spread along the horizontal axis is arbitrary and for display purposes only. Asterisks indicate probability that the true median equaled the null hypothesis (horizontal line; * $p < 0.05$; *** $p < 0.001$; two-sided Wilcoxon signed rank test). Null hypotheses for beta and non-beta parameters were zero and one, respectively. Specific values for medians and associated p-values for monkey N or K, respectively, are as follows: β_{constant} = -3.7 or -1.9, p = $3*10^{-8}$ or $3*10^{-26}$; β_{offer} = 20 or 9.0, p = $3*10^{-8}$ or $3*10^{-26}$; δ = 0.77 or 0.82, p = $3*10^{-8}$ or $3*10^{-26}$; and, for monkey K only, γ = 0.83, p = 0.03.

In summary, both monkeys were significantly more likely to accept an offer the larger its magnitude, though with an overall tendency to reject all offers and a maximal acceptance rate for the largest offers around 80%. In addition, monkey K exhibited a sub-linear utility function as reflected in the fitted values of the parameter γ (see Methods), i.e., the increase in utility for a given increase in offer size became less as offer size increased.

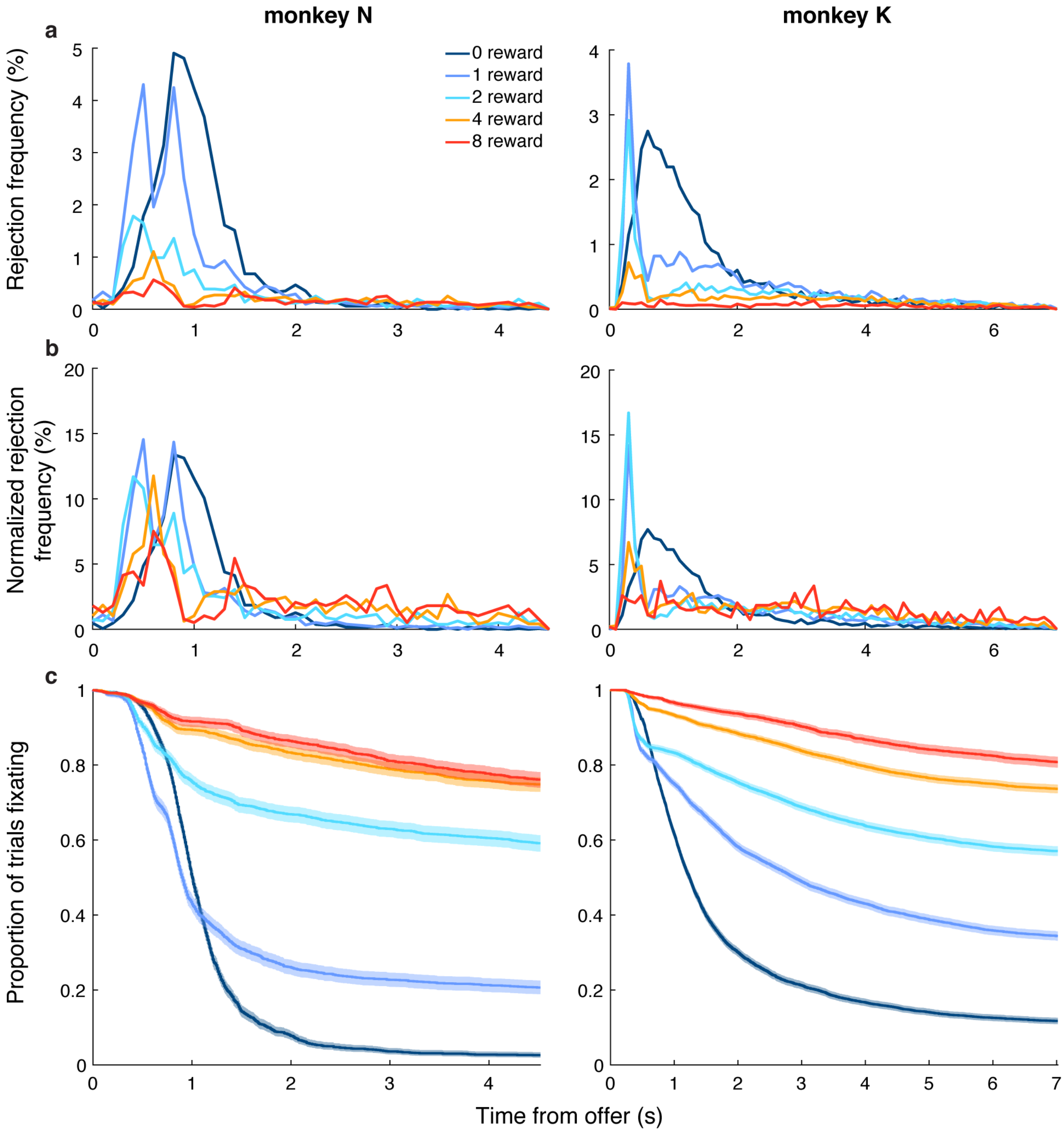

Supplementary Figure 2. Rejection timing.
(a,b) The frequency of fixation breaks (i.e., reject choices) per 100 ms time bin is shown for each offer size (colors, see legend) as a function of time from the onset of the offer period as the percentage of all rejections (a) or as percentage of rejections for the given offer size (b) for monkeys N and K (left and right panels, respectively). **(c)** The Kaplan-Meier estimator of the fixation survival function is shown for each offer size (colors as in (a,b)) as the proportion ± 95% confidence interval (shading) of trials in which the animal is fixating as a function of time from the onset of the offer period. The derivative of the survival function conditioned on maintenance of fixation approximates the hazard rate functions shown in Figure 1c. The hazard rates scaled inversely with offer size (Cox proportional hazards, excluding 0-rewards: β_{offer} ± s.e.m. = -0.29 ± 0.0098 or -0.29 ± 0.0069, $p < 10^{-16}$ or 10^{-16} (less than machine precision), monkey N or K, respectively; see Supplementary Figure 3 regarding 0-reward offers).

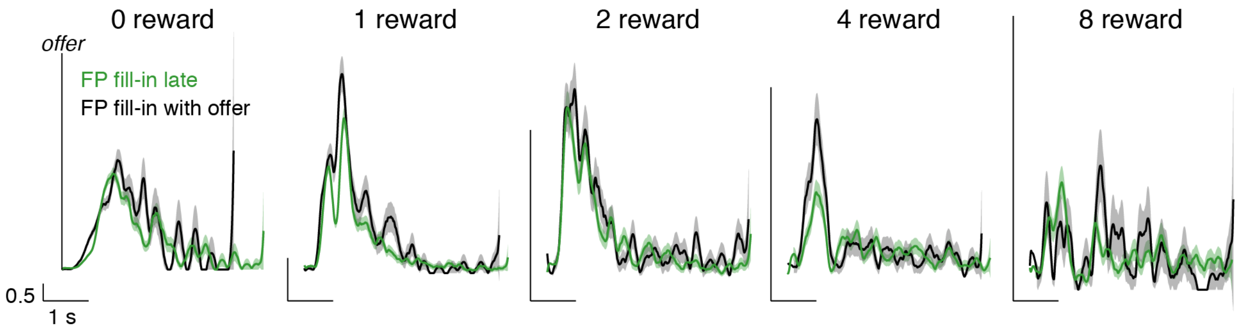

Supplementary Figure 3. FP fill-in timing and effect on rejection time.
In a subset of early experimental sessions for monkey N, the fixation point (FP) transitioned from an open annulus to a filled circle at the beginning of the work period (FP fill-in late), instead of the beginning of the offer period (FP fill-in with offer), as in all other sessions. Therefore, for these early sessions, the animal did not receive an explicit cue as to the beginning of the offer period on 0-reward offers (in contrast, on non-zero offers, the onset of the offer icons cued the offer period). We reasoned that this 0.5 s delay on 0-reward offers may have contributed to a delay in responding to the 0-reward offers in particular, and potentially to any size offer. Here we plot the hazard rate ± standard deviation (shading) of fixation breaks as a function of time from the offer (as in Figure 1c), with the rate shown separately for FP fill-in late (green curves) vs. FP fill-in with offer (black curves) sessions. Vertical and horizontal scale bars indicate 0.5 hazard rate and 1 s duration, respectively.

For all offer sizes, we observed no appreciable differences in the temporal profile of hazard rate depending on the timing of the FP fill-in cue. We concluded that the animal did not rely strongly on the FP fill-in cue in determining the timing of rejection responses. Nonetheless, regardless of FP timing, we observed a marked delay in hazard rates for the 0-reward offer compared to all other offers for both animals N and K (see Figure 1c and Supplementary Figure 2). The cause of this delay was unclear. The 0-reward cue may have been less salient, contributing to slower reaction times. However, intuitively, the distinction between FP fill-in late vs. with offer would likely have been more salient, and yet the FP timing did not account for the slower responses to 0-reward offers. This suggested that a temporal component of the decision process was fundamentally different for zero vs. non-zero offers and argued for analyzing the timing of 0-reward offers separately (as was done for the Cox proportional hazards analysis in Supplementary Figure 2).

Anatomy

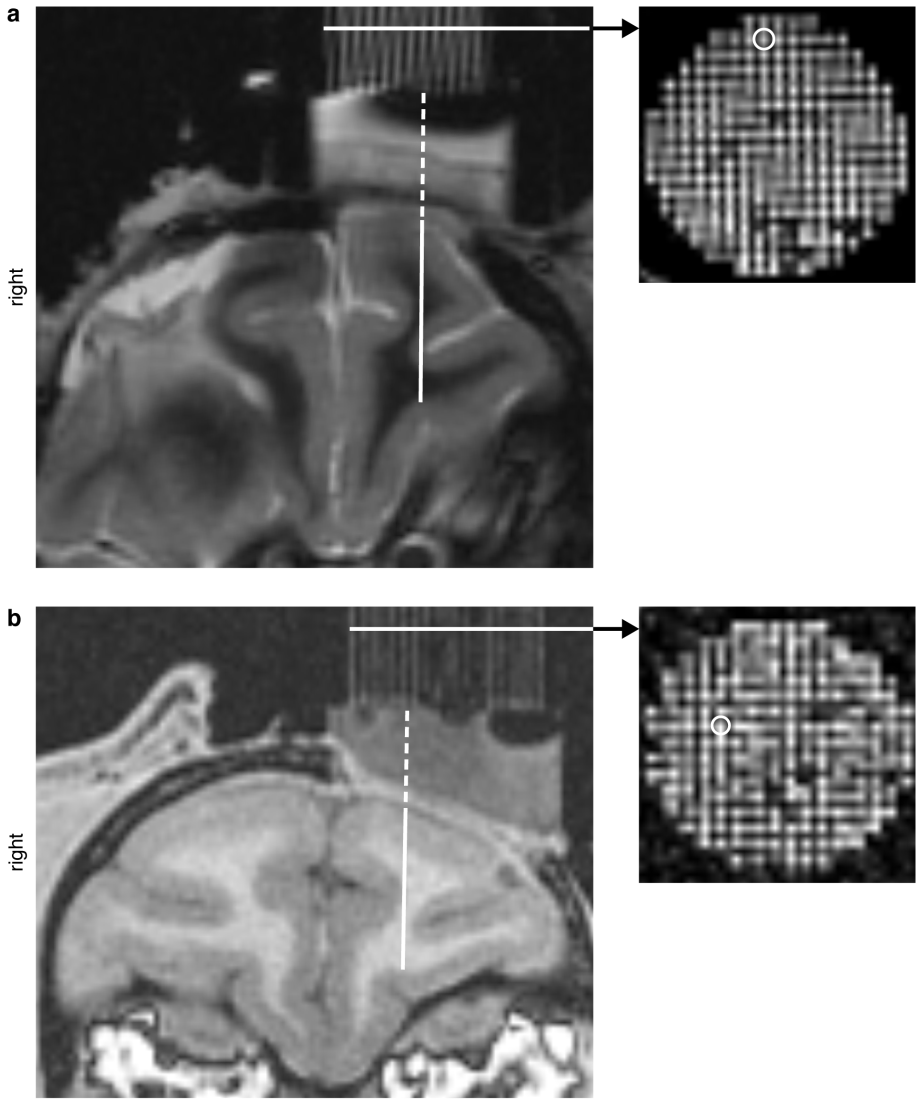

Supplementary Figure 4. MRI localization.
Anatomical MRI sequences were acquired with recording grid in place prior to physiological recording. **(a,b)** T2- or T1-weighted coronal slice is shown at the median anterior-posterior extent of recorded sites for monkey N (a) or K (b), respectively. Animal's right side is shown on the left side of the image, per radiological convention. The inset shows an axial slice through the recording grid (white horizontal arrow), with a representative grid hole circled corresponding to grid position 2A or 0G (43.1 or 36.6 mm anterior to the interaural line, measured via intra-surgical stereotaxic coordinates; 6.7 or 6.3 mm left of midline, measured from the post-surgical MRI) for monkey N or K, respectively (see Supplementary Figure 6). Recording grid was filled with salinized agarose solution to facilitate contrast on MRI. By registering the axial slice through the recording grid and the coronal slice containing the selected grid position, we traced the virtual electrode trajectory from the bottom of the recording grid to the dorsal surface of cortex (vertical dashed white line), and then from the dorsal surface to the ultimate recording site (vertical solid white line) for individual electrode penetrations. We estimated position along the trajectory with a calibrated micromotor. Here we show the virtual trajectory to the most dorsal aspect of OFC reached by the representative grid positions (above): the fundus or lateral bank of the medial orbital sulcus at 14.5 or 13.2 mm below the dorsal surface for monkey N or K, respectively. Note that the coronal slice prescription for monkey N was oblique to the coronal plane with the left hemisphere rotated anteriorly, accounting for the asymmetry in (a).

Properties of individual-unit contributions to low-dimensional representations

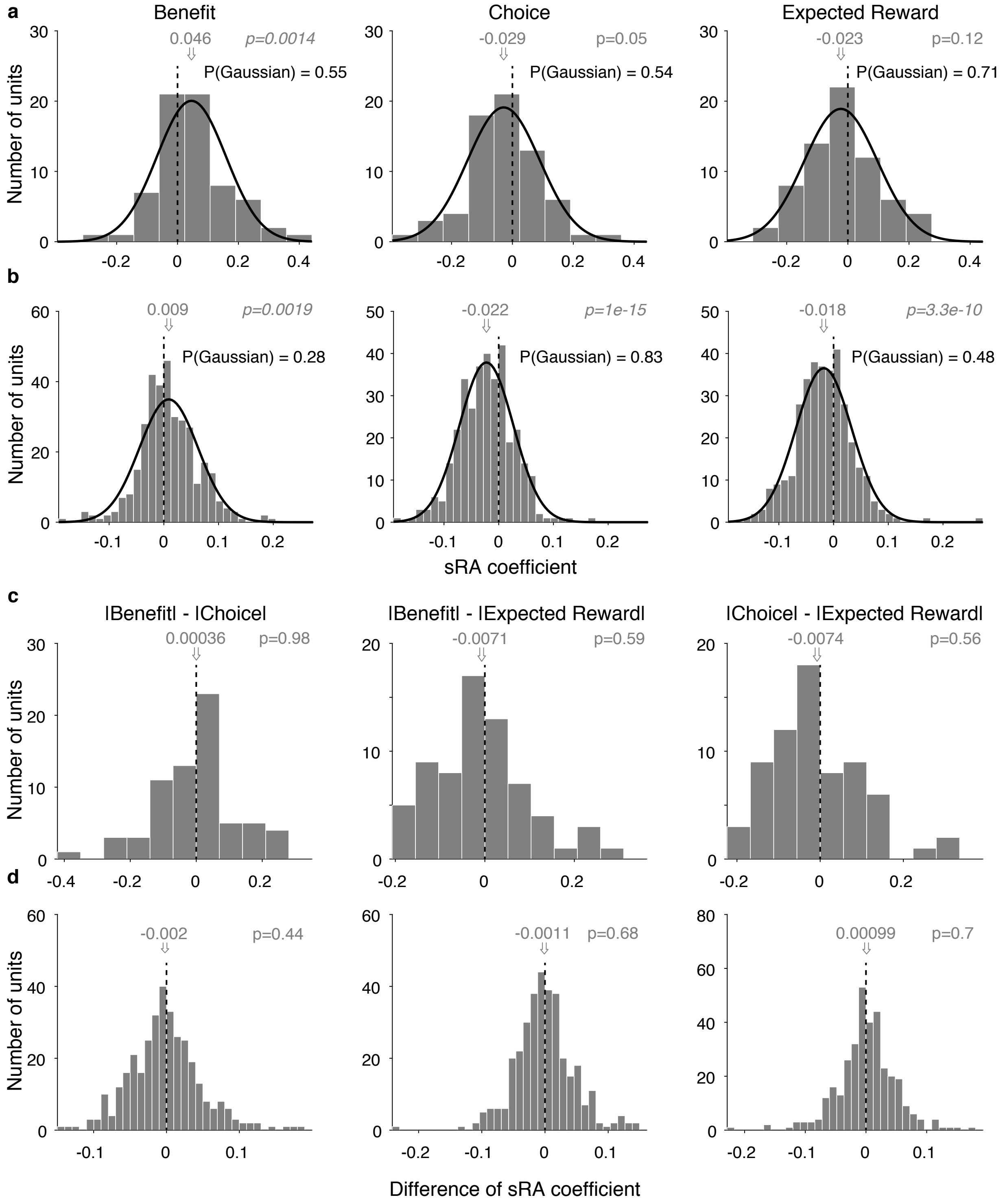

Supplementary Figure 5. Contribution of individual units to low-dimensional representations.
(a,b) Histogram of regression coefficients across the population that specified each unit's contribution to the static low-dimensional representations (sRAs) of BENEFIT (left panel), CHOICE (middle panel), and EXPECTED REWARD (right panel) for monkeys N (a) and K (b). Distribution mean (gray arrow and text) and p-value (gray text) from two-tailed *t*-test of null hypothesis that mean = 0 (vertical dashed line) are shown.

Gaussian functions were fit to each distribution (black curves). Probability (black text) of null hypothesis that observed distribution was Gaussian was computed via two-tailed Kolmogorov–Smirnov test. No distribution differed significantly from Gaussian. **(c,d)** Histograms of difference between absolute value of regression coefficients for pairs of representations within the same unit (|BENEFIT| - |CHOICE|, left; |BENEFIT| - |EXPECTED REWARD|; middle; |CHOICE| - |EXPECTED REWARD|, right) are shown for monkeys N (c) and K (d). All conventions as in (a,b). No distribution of differences differed significantly from zero, i.e., the absolute strength of encoding did not differ for linear (i.e., benefit and choice) vs. non-linear (i.e., expected reward) variables, as observed in other prefrontal areas[7] but not in parietal cortex, where linear terms were represented preferentially[8]. The encoding of linear vs. non-linear variables maps directly onto the concepts of linear vs. non-linear mixed selectivity, which confer distinct advantages for population decoding[9]. For present figure, all regression coefficients were found without orthogonalizing the sRAs.

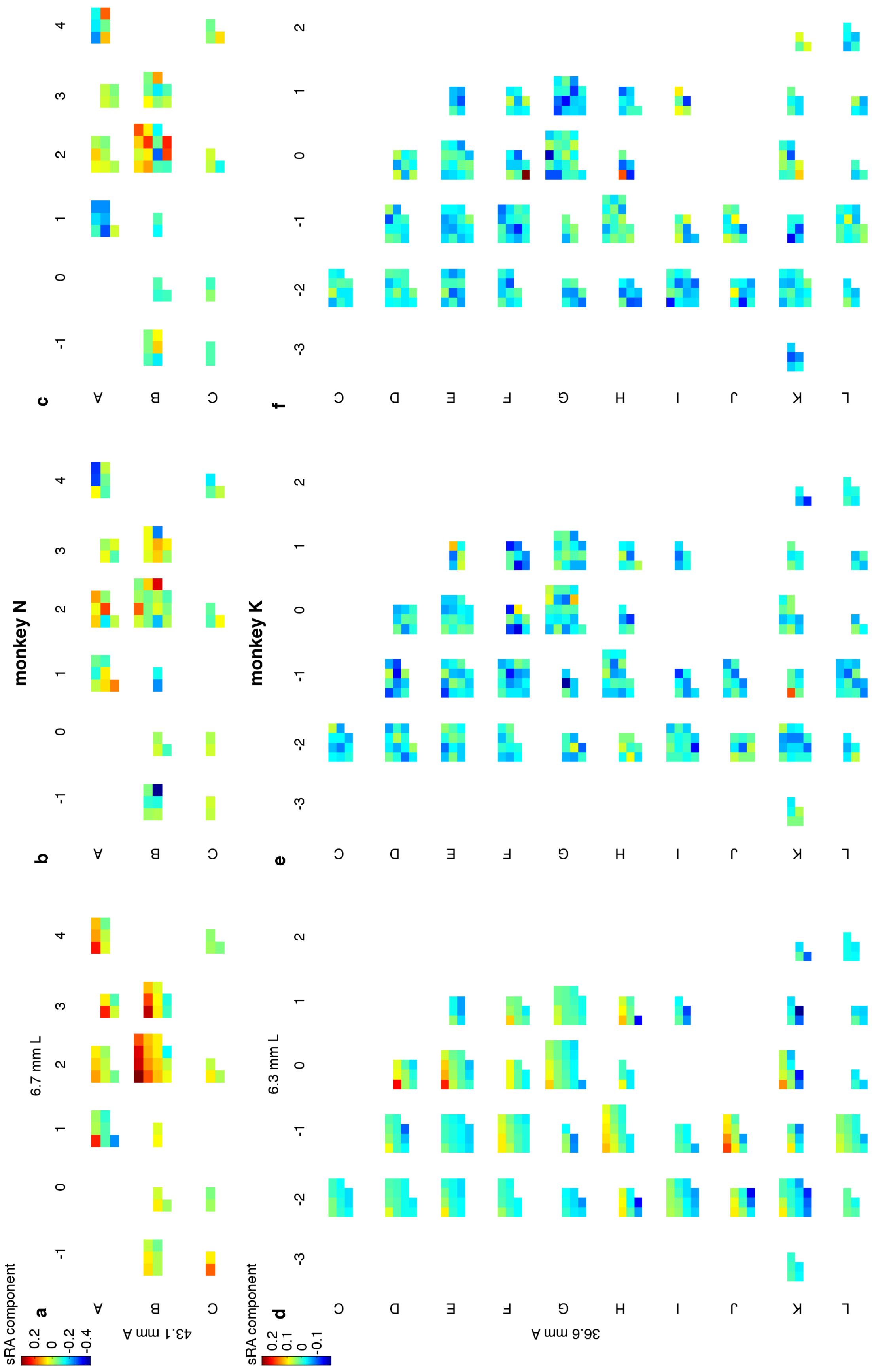
Benefit
Choice
Expected Reward
monkey N
monkey K
6.7 mm L
6.3 mm L
43.1 mm A
36.6 mm A
sRA component
sRA component
a
b
c
d
e
f

Supplementary Figure 6. Anatomical distribution of sRA coefficients.
The contribution of each unit to the low-dimensional representations (sRAs) of BENEFIT (a,d), CHOICE (b,e), and EXPECTED REWARD (c,f) are shown as a function of anatomical recording site for monkeys N (a-c) and K (d-f). Each pixel corresponds to an individual unit and the pixel color (referencing animal-specific color scale at left) indicates the value of the regression coefficient, i.e., the contribution of that unit to the corresponding sRA. Units (pixels) recorded from the same recording grid position (irrespective of electrode depth) are grouped together (roughly rectangular blocks) and, within each group, ordered (left to right, then top to bottom) by increasing values of the BENEFIT coefficient; the same order is preserved for the other sRAs. Blocks are arranged by corresponding 1 mm x 1 mm recording grid position from anterior to posterior (top to bottom rows) and medial to lateral (left to right columns). The grid row (letter) and column (number) labels are arbitrary. In panels (a,d), the coordinates of the grid position (2A or 0G for monkey N or K, respectively) referenced in Supplementary Figure 4 are labeled in mm anterior to the interaural line and left of midline. Note that the anterior-posterior coordinate was measured stereotaxically in reference to the center of the recording cylinder at the surface of the skull, ~30mm superior to the recording sites. Therefore, the AP coordinates of the actual recording sites may differ given small deviations in electrode trajectory in the coronal plane. In contrast, the left-right coordinate was measured in the MR images at the level of the recording site.

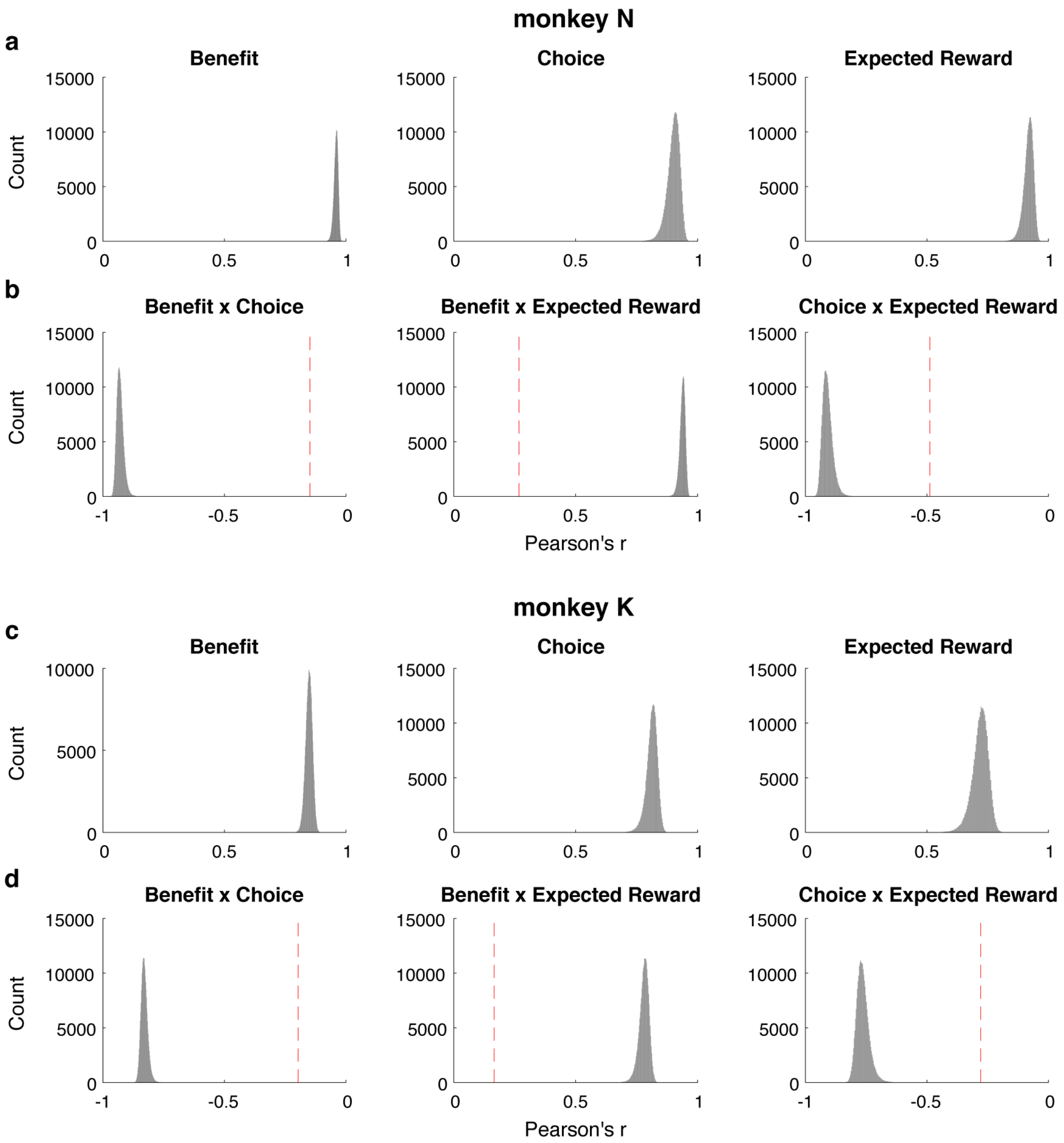

Supplementary Figure 7. Reliability of and correlation between low-dimensional representations. We generated S = 700 sampling datasets by randomly selecting Q of Q trials with replacement for each unit, and discovering the trio of non-orthogonalized sRAs independently for each dataset (Methods). To estimate the reliability of the sRAs, we computed the Pearson's correlation $\tilde{r}_{ii}$ between sRAs for variable $i \in$ {BENEFIT, CHOICE, EXPECTED REWARD} from all pairs of sampling datasets (i.e., for S datasets, we measured $(S^2 - S)/2$ pairs per variable). **(a,c)** Histograms of $\tilde{r}_{ii}$ revealed very high reliability of the sRAs ($\tilde{r}_{ii}$ > 0.92 or 0.75 for monkey N (a) or K (c), respectively; Supplementary Table 2).

We were interested in the separability between representations of distinct variables i and $j \in$ {BENEFIT, CHOICE, EXPECTED REWARD}. We defined representations of i and j as *separable* when the correlation r_{ij} between sRAs for variables i and j was less than expected by chance given a null model in which representations of i and j were perfectly correlated (or anticorrelated, i.e., $|r| = 1$), but subject to imperfect reliability (i.e., independent noise), as estimated by $\tilde{r}_{ii}$ and $\tilde{r}_{jj}$, thus resulting in an observed

absolute correlation of less than unity. We defined the null model empirically as $\tilde{r}_{ij} = \pm\sqrt{\tilde{r}_{ii}}\sqrt{\tilde{r}_{jj}}$ (Methods) and took only the positive values. For display purposes only, we multiplied $\tilde{r}_{ij}$ by the sign z of the observed correlation r_{ij} (all statistical tests were performed on $|r_{ij}|$ and $\tilde{r}_{ij}$). **(b,d)** Histograms of the null model $z\tilde{r}_{ij}$ for monkeys N (b) and K (d) are shown for each pair of variables *i* and *j* and compared to the observed correlation r_{ij} (vertical dashed red line). For all pairs of variables, the between-variable correlations were found to be highly separable (i.e., $|r_{ij}| < \tilde{r}_{ij}$ via 1-tailed t-test, $p < 10^{-16}$ (less than machine precision); Supplementary Table 2).

Activity of low-dimensional representations

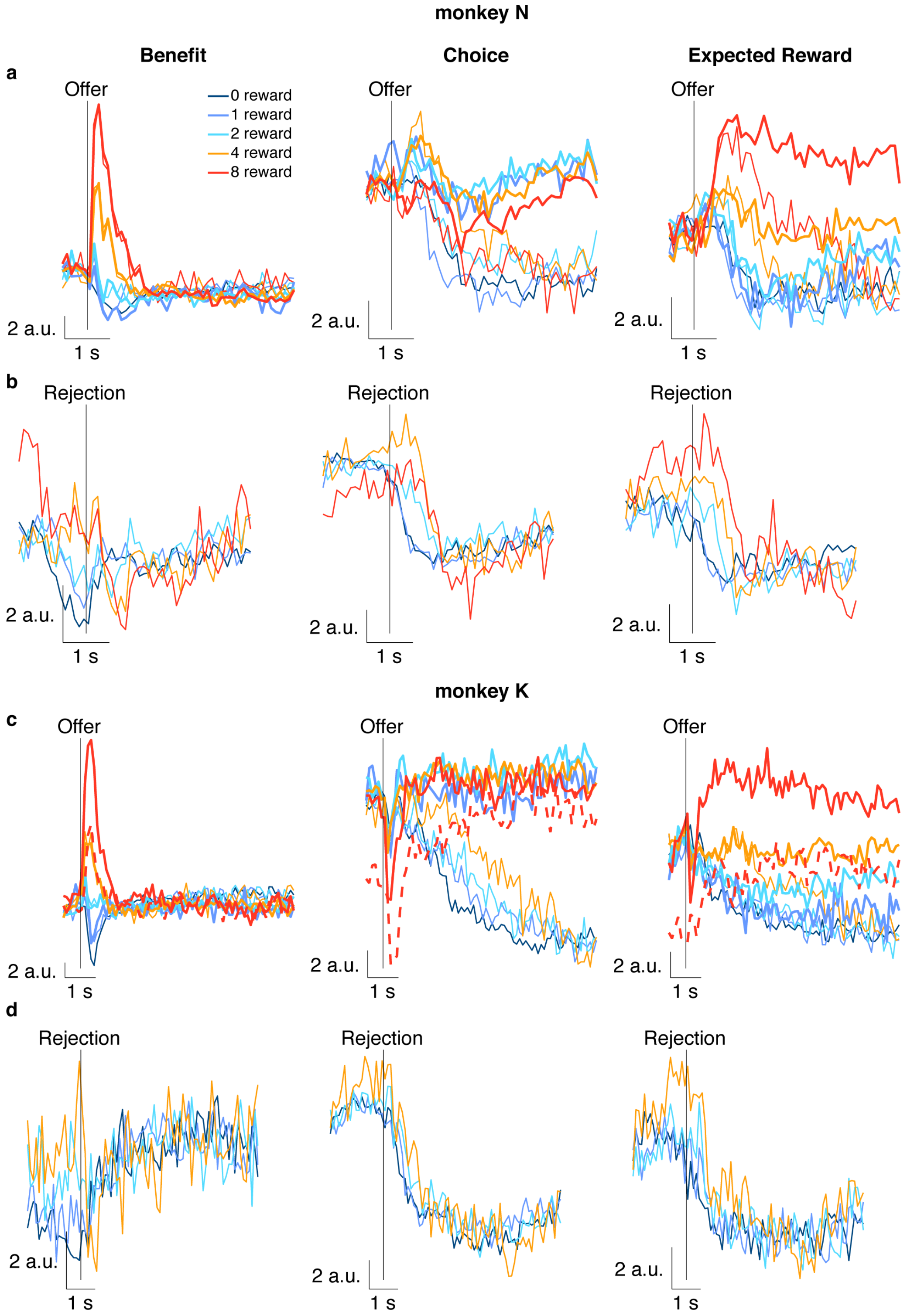

Supplementary Figure 8. Activity of low-dimensional representations aligned to offer and rejection.

Neural responses $\bar{\bar{R}}$ (which included the common-condition response; see Methods) were aligned to the time of the offer **(a,c)** or rejection **(b,d)** for monkeys N (a,b) and K (c,d) and projected onto the low-

dimensional representations (sRAs) of BENEFIT (left panels), CHOICE (middle panels), and EXPECTED REWARD (right panels). The response for each combination of offer size (colors, see legend) and choice (accept or reject choices in thick or thin curves, respectively) is shown as a function of time.

We examined these projections onto the sRAs (i.e., sRA *activity*) so as to interpret the gradual separation of CHOICE activity for accept and reject choices seen in Figure 4a,b. The slow dynamics could arise from underlying single-trial responses with similarly slow dynamics (i.e., “ramps”), or from single-trial responses with rapid changes in activity (i.e., “steps”), but for which the trial-average responses are temporally blurred due to trial-to-trial variability in the rejection time.

By including the common-condition response and aligning to the offer (a,c), we again observed gradual separation of accept and reject responses for both animals. However, for monkey N, we now observed a rapid change in CHOICE activity that preceded the separation and therefore was common to accept and reject choices. In addition, the subsequent gradual separation was equally due to the slow recovery of the accept responses and not due exclusively to further divergence of the reject responses, as would be predicted if reject choices were associated with temporally blurred step-like dynamics. By aligning to the rejection (b,d), we observed a rapid change in the reject response after the rejection; however, this change was comparable in rate and magnitude to the offer-aligned change shared by accept responses, thus suggesting the rapid rejection-aligned dynamics were explainable by a common-condition response to the offer (i.e., common to accept and reject choices) and not a rapid step-like response to the rejection.

In contrast, for monkey K, we observed that the offer-aligned separation of accept and reject responses was primarily driven by a gradual change in the reject response (c). When aligning to the rejection (d), the dynamics of the reject response appeared qualitatively more rapid than when aligned to the offer, suggesting that the separation of accept and reject responses was likely driven by a rapid change in neural activity on reject trials that was likely temporally aligned to the time of the rejection.

Taken together, the separation of CHOICE activity into accept and reject responses may derive from different sources for the two animals: a slowly evolving response to accept choices (i.e., ramp) in monkey N, whereas a rapid response to the rejection (i.e., step) in monkey K.

In addition, we considered the apparent “bleed through” of offer size information onto the CHOICE sRA when aligning to the offer, particularly for reject choices (a,c, middle panels). Notably, the choice selectivity appeared increasingly offset in time for larger offers. As the median rejection times were later for larger offers (Supplementary Figure 2), this suggested that the apparent offer-selectivity was merely choice-selectivity arising at systematically later delays for larger offers. We tested this prediction by aligning to the time of rejection (b,d, middle panels), which would eliminate differential responses to offer size that were merely due to different rejection times. For monkey K, we observed no sensitivity to offer size (d), consistent with our prediction. However, for monkey N, the rejection-aligned responses showed less sensitivity to offer size than when aligned to the offer (b vs. a), but were still somewhat sensitive to offer, implying some overlap in the dimensions that represented benefit and choice mid-trial—overlap we examined specifically in Supplementary Figure 24.

Finally, we considered the contribution of post-rejection gaze to differential accept vs. reject CHOICE activity. For monkey N, the fact that the choice discrimination was primarily due to changes from baseline on *accept* trials (a, middle panel, thick lines) suggested that gaze on *reject* trials played little role in differential CHOICE activity. Likewise, the lack of evidence for a post-rejection (i.e., post-saccadic) phasic, or step-like, response in monkey N, which might be expected if reject responses were highly gaze dependent, further argued against gaze-related activity driving differential CHOICE responses. However, for monkey K, the choice discrimination appeared to be driven by the reject responses and evidence for a post-rejection phasic response was stronger (discussed above); thus we could not exclude the role of post-rejection gaze in monkey K.

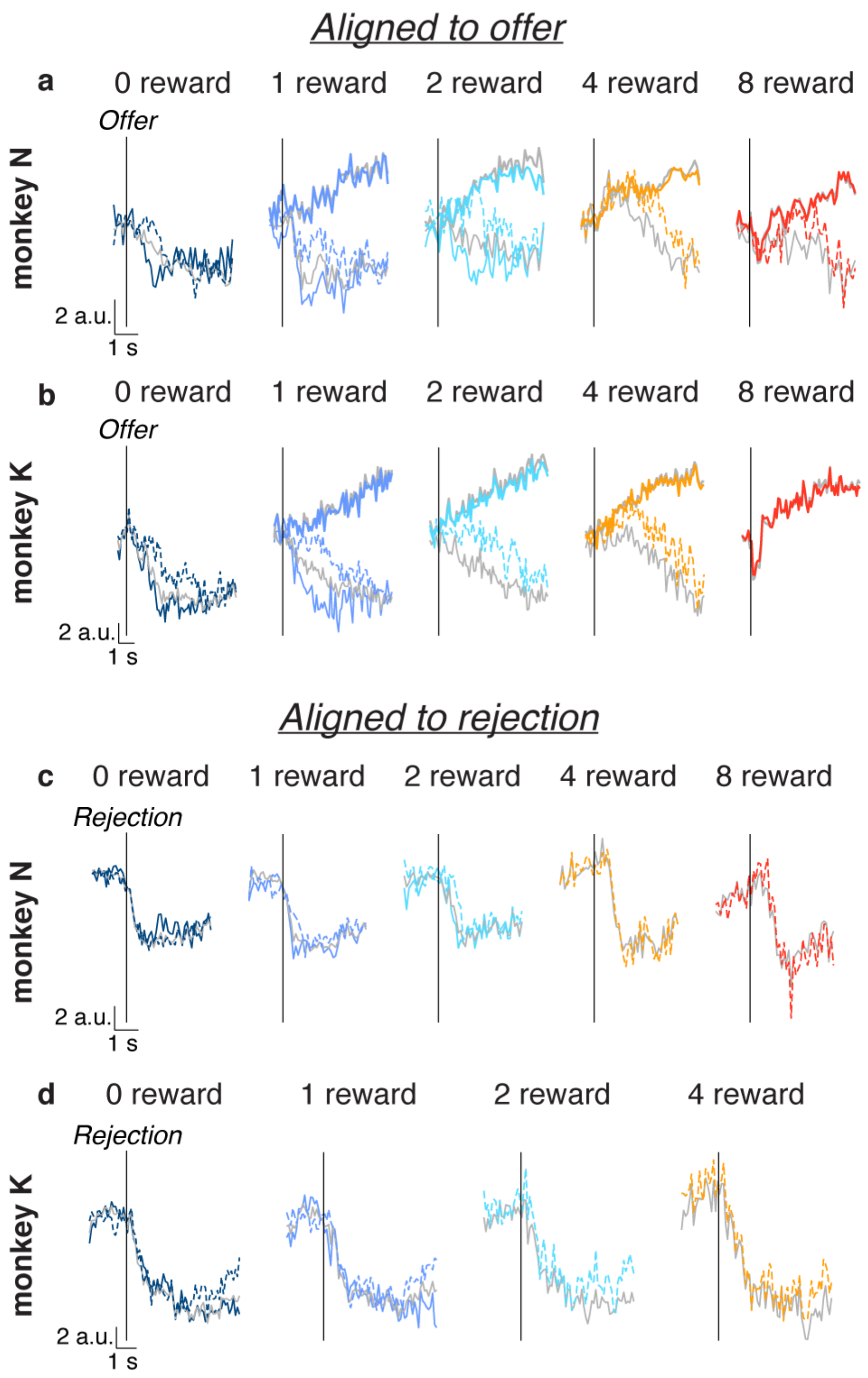

Supplementary Figure 9. Relating neural dynamics to decision dynamics.
Here we relate the low-dimensional neural dynamics to the trial-level decision dynamics. Specifically, we reasoned that if the CHOICE sRA reflected the animal's decision process, then the time at which it discriminated the animal's choice should depend on the time at which the choice was rendered. Our serial recordings precluded a trial-by-trial analysis linking variation in sRA dynamics to choice timing, and so instead we compared sRA activity between trials stratified into either early or late choices.
(a-d) The time course of the high-dimensional population activity projected onto the CHOICE sRA (i.e., CHOICE *activity*) is shown on separate panels for each offer size (colors, as labeled) and separated by accept and reject choices (thick and thin curves, respectively) for monkeys N (a,c) and K (b,d). Reject

choices are further separated by *early* and *late rejections* (solid and dashed curves, respectively), defined as occurring before or after, respectively, the median rejection time across all offers for each animal (0.92 or 1.3 s, monkey N or K). In **(a,b)**, the responses are aligned to the onset of the offer and mean-subtracted (as in Figure 4a,b); in **(c,d)**, the responses are aligned to the rejection but are *not* mean-subtracted (as in Supplementary Figure 8b,d). Colored curves reflect activity of the subset of units with adequate trial counts for the present analysis (see below). For reference, the projections for *all* accept and reject trials from the full population are shown in thick and thin gray lines, respectively (identical to middle panels in Figure 4a,b or Supplementary Figure 8b,d).

Aligning to the offer (a,b), the CHOICE activity diverged from accept choices (thick curves) at a later time for late than early rejections (dashed vs. solid color thin curves). For offers with too few early rejections to be observed separately, we compared late rejections and *all* rejections (dashed color vs. solid gray thin curves), which also showed a delayed divergence for later rejection times. This sensitivity of CHOICE activity on rejection time is consistent with our proposal that the sRA dynamics reflected the underlying decision dynamics. When aligning to the rejection (c,d), the CHOICE activity was virtually identical for early vs. late rejections both before and after the rejection, further supporting the thesis that the CHOICE sRA reflected the binary state of the animal's decision at the time of the choice, independent of the time elapsed since the offer. As discussed in Supplementary Figure 8, we could not definitely exclude the contribution of post-rejection gaze to the differential CHOICE activity. However, we observed that the breaking eye movements were smaller for later rejections and for larger offers (not shown), consistent with accidental fixation breaks (see Figure 1c and related main text). Despite these overt post-rejection behavioral differences between early vs. late rejections, they did not manifest as differences in the post-rejection CHOICE activity for early vs. late rejections, further reducing the likelihood that the CHOICE sRA reflected an indirect consequence of the decision, rather than the decision itself.

The activity of the BENEFIT and EXPECTED REWARD sRAs is not shown for simplicity. In summary, activity of the BENEFIT sRA was virtually identical for early- and late-rejections. Though the animal's initial valuation of the offer may have influenced rejection time (i.e., presumably later rejections reflected higher initial valuation), any differences in initial valuation were not reflected in the BENEFIT sRA activity, consistent with the lack of a choice predictive, post-offer signal in the single-neuron analysis (Supplementary Figure 11). The relationship between rejection timing and the EXPECTED REWARD activity was equivocal. However, to the extent that activity for late rejections differed from either early or all rejections, the late-rejection activity tended to hew more closely to the activity on the corresponding accept trials. This trend was consistent with EXPECTED REWARD encoding the animal's real-time valuation that, in turn, informed how likely the animal maintained its initial behavioral policy to accept the offer.

Condition and unit selection:

As in the main text, certain conditions—now defined by offer, choice, *and* rejection time—had too few trials per unit to accurately estimate trial-average responses and were excluded. We required that a given condition have at least 5 trials per unit for that condition to be included, as in the main text. To preserve as many conditions as possible in the present analysis, we allowed for conditions that did not meet this minimum trial count for all units, but did meet the criterion for at least 85% of units. As such, we eliminated 2 of the original 9 conditions for monkey N (early rejections of 4- and 8-reward offers) and 2 of the original 8 conditions for monkey K (early rejections of 2- and 4-reward offers). (Recall that some conditions had already been eliminated for insufficient trial count in the main analysis.) Units not meeting the minimum trial count for one or more of the included conditions were eliminated: 16 of 68 units (24%) for monkey N and 73 of 342 units (21%) for monkey K.

To allow for comparison with the main text, we preserved the CHOICE sRA coefficients from the main text. However, because we eliminated certain units in the present analysis, we effectively eliminated the corresponding dimensions from the sRA, necessarily reducing the magnitude of the new projections. To assess the impact on magnitude, we compared the accept trials between the full and pared-down populations (gray vs. color *thick* curves) and observed only a minor decrement. This decrement was markedly smaller than the difference between all rejections in the full population and either the early or late rejections in the pared-down population (gray vs. colored *thin* curves), meaning these differences between rejections in the full vs. pared-down populations were unlikely due to the changes in population membership. Note that the principal comparisons between accept choices, early rejections, and late rejections (colored curves), discussed above, were all within the pared-down population, and thus were not attributable to changes in population membership.

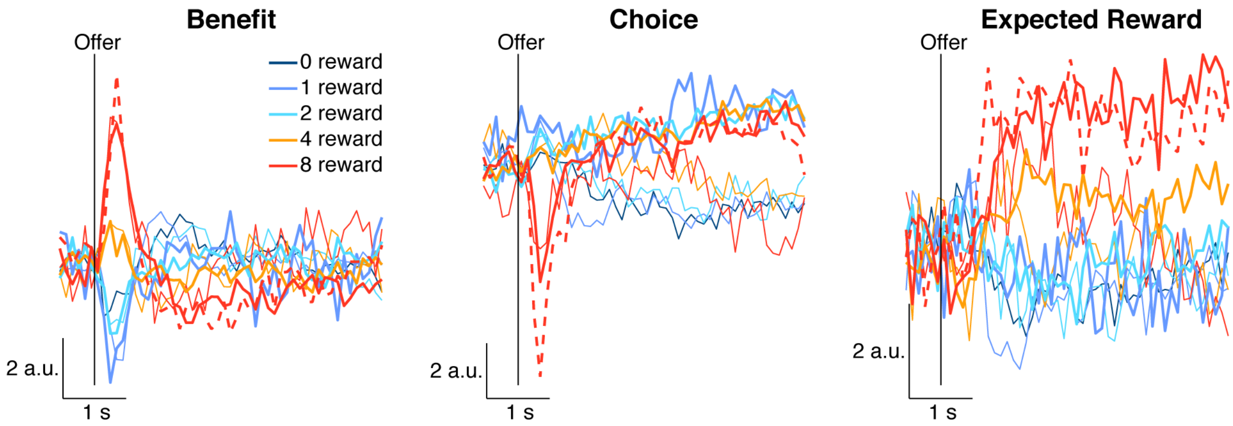

Supplementary Figure 10. Activity of low-dimensional representations including singleton offer for monkey N.
We separately discovered the low-dimensional representations (sRAs) in a subpopulation limited to units recorded with the singleton offer for monkey N. (For monkey K, all units were recorded with the singleton offer, as shown in Figure 4b). As for monkey K, the singleton response was not included when computing the sRAs. We projected the population response onto the sRAs of BENEFIT (left), CHOICE (middle), and EXPECTED REWARD (right) for each offer size (colors, see legend) and accept and reject choices (thick and thin curves, respectively) as a function of time from the onset of the offer period (vertical black line). The response to the singleton offer (thick dashed red curve) was comparable to the value-matched, 8-reward non-singleton offer (thick solid red curve), consistent with the OFC population encoding the value and not visual properties of the stimulus. The animal rarely rejected the singleton offer, and so too few trials existed to analyze this condition.

Choice probability in individual units

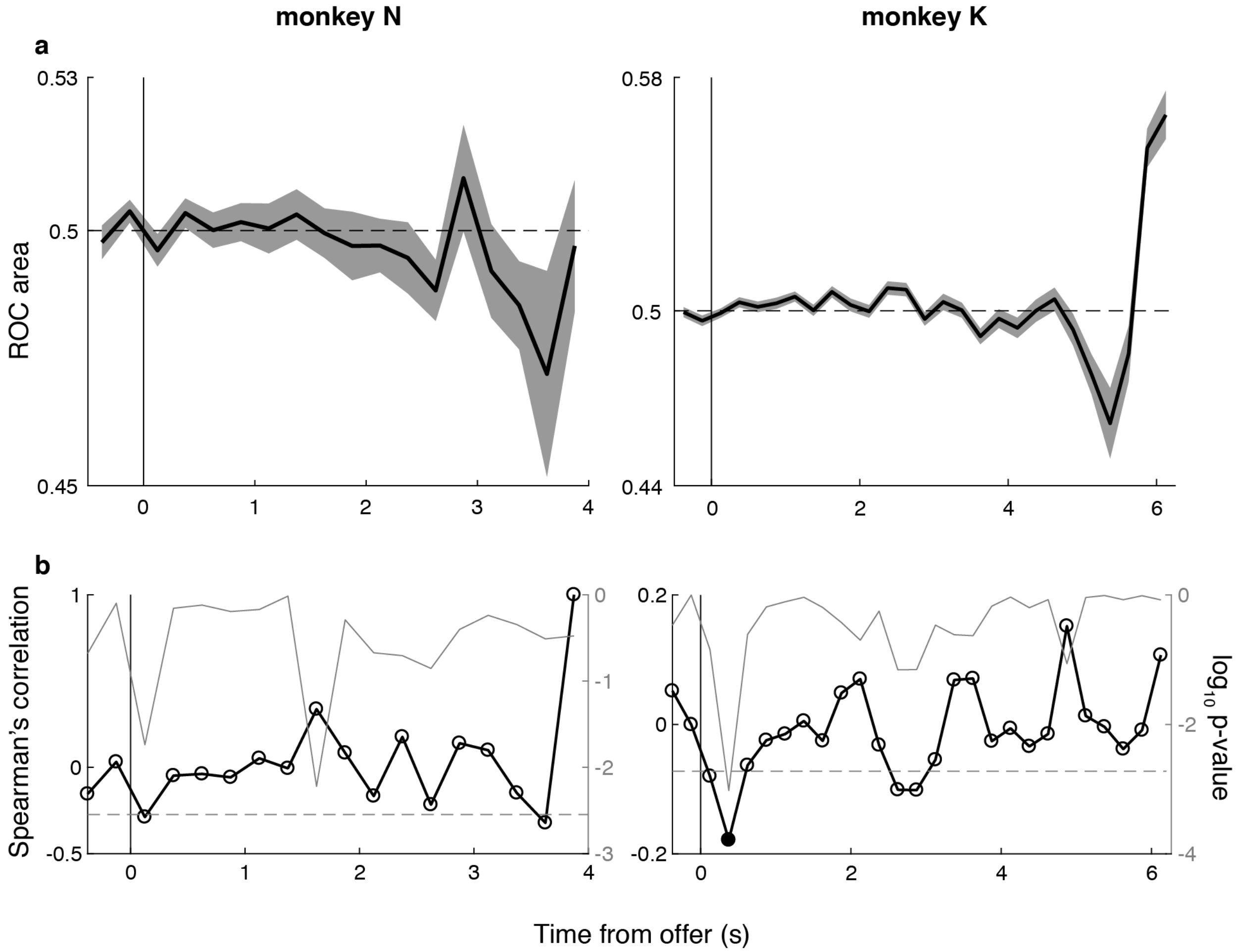

Supplementary Figure 11. Choice probability in individual units.
We reasoned that if OFC activity were used by downstream circuits to estimate the value of a given offer, and that trial-to-trial fluctuations in value accounted for variability in choice of a given offer size, then we would expect trial-to-trial variation in OFC activity to predict the animal's choice. Choice-predictive responses have been observed in sensory areas representing the sensory stimulus in perceptual decision-making studies[10,11]. However, evidence for robust choice-predictive responses in OFC has been equivocal[12,13]. We quantified the choice predictivity in OFC using the well-established choice probability (CP) metric, as described elsewhere[10]. Because our analysis of the population representations (i.e., sRAs and dRAs) relied on trial-average responses pooled across sessions (and thus could not exclude post-rejection activity on single trials), we measured CP at the individual-unit level.

Briefly, for each unit, we extracted the neural response r(t,T) in 250 ms non-overlapping time bins t aligned to the offer presentation on each trial T. So as to isolate responses *predictive* of an upcoming choice, we excluded trials for a given time bin in which the animal had rejected the offer prior to that time bin. Therefore, later time bins necessarily included fewer trials. To control for the known influence of offer size on the neural response, we z-transformed r(t,T) within each offer size (provided at least two trials were observed for a given offer and time bin) and combined responses across offers to give the normalized response R(t,T). We stratified R(t,T) according to the choice on trial T and compared the distributions of responses on accept vs. reject trials at each time bin t, which we quantified as the area under the receiver operator characteristic (ROC) curve, or equivalently, CP(t)—the probability of an ideal observer correctly classifying a trial as an accept choice given the neural response[14]. We eliminated time bins with fewer than 10 trials (across offers) contributing to either the accept or reject distribution. CP(t) =

0.5 indicated chance performance. Values significantly greater or less than 0.5 indicated the response was choice predictive, with higher firing rates predicting accept or reject choices, respectively.

(a) The median CP(*t*) across units (i.e., ROC area; thick black curve) is shown as a function of time from the onset of the offer period for monkeys N and K (left and right panels, respectively). Gray shading indicates +/- the median absolute difference (i.e., median(|CP(*t*) – median(CP(*t*))|)). Note that the curves do not extend the full length of the trial because the later time bins had too few trials to include (i.e., most rejections had occurred by this time). In no time bin was CP(*t*) significantly different from chance by Wilcoxon signed rank test of null hypothesis that median CP = 0.5 (horizontal dashed-line), two-sided and Sidak-corrected for multiple time bins.

The computation of CP required analysis of single-trial data so as to isolate responses occurring before the time of rejection, a time which varied across trials. In contrast, all population-level analyses, such as oTDR, depended on trial-average data so as to pool across serially recorded units. Thus, the choice predictive analyses were limited to individual units. In an effort to relate the above individual-unit analysis to the population-level analyses discussed in the main text, we compared CP(t) to the contribution individual units made to the population low-dimensional representations (sRAs). Specifically, we reasoned that units representing variation in value *across* offer sizes (as measured by their contribution to the BENEFIT sRA) may have represented trial-to-trial fluctuations in value *within* an offer size (as measured by CP), a relationship observed for other brain regions (e.g., in visual area MT, units encoding visual motion direction across different levels of motion strength also predicted the subject's perceptual report within a given level of motion strength[15]). In addition, this hypothesis would address a second limitation of averaging CP across units. Namely, averaging assumed a common sign of choice predictive encoding (e.g., firing more before accept vs. reject choices), whereas the sign of encoding may have been heterogeneous across the population, which would be obscured by simple averaging. By relating CP to the encoding of offer size, we inferred the sign of within-offer value encoding (i.e., CP) from the sign of between-offer value encoding (i.e., coefficient for BENEFIT sRA).

(b) Specifically, we computed the Spearman's correlation coefficient (thick black curve and open circles, referencing left ordinate) between CP(*t*) for a given unit and the BENEFIT sRA coefficient corresponding to that unit as a function of time *t* from the onset of the offer period for monkeys N and K (left and right panels, respectively). The associated $\log_{10}$ probability p of observing the correlation coefficient by chance (thin gray curve; p-value from Matlab's *corr* function, which uses the Fisher transformation of the correlation, one-sided, uncorrected) is plotted on the same panels and references the right ordinate. We marked as significant (filled circles) those time bins when p was less than a 0.05 threshold after Sidak-correction for multiple comparisons across time bins (corrected p-threshold shown as gray horizontal dashed line). At no time was a significant correlation observed for monkey N, and only a single time bin was significant for monkey K (filled circle).

In summary, individual units in OFC did not demonstrate choice predictivity. That is, on average, trial-to-trial variation in firing rate was not systematically related to trial-to-trial variation in choice. In addition, we did not observe a systematic relationship between a unit's putative choice predictive signal (i.e., CP) and its contribution to the population's encoding of offer value (i.e., BENEFIT sRA), with the exception of a single time bin for monkey K only. To address the question of choice predictivity in OFC, simultaneous recording of many units would be required to observe single-trial choice representations at the level of the population.

Sensitivity and specificity of the low-dimensional representations

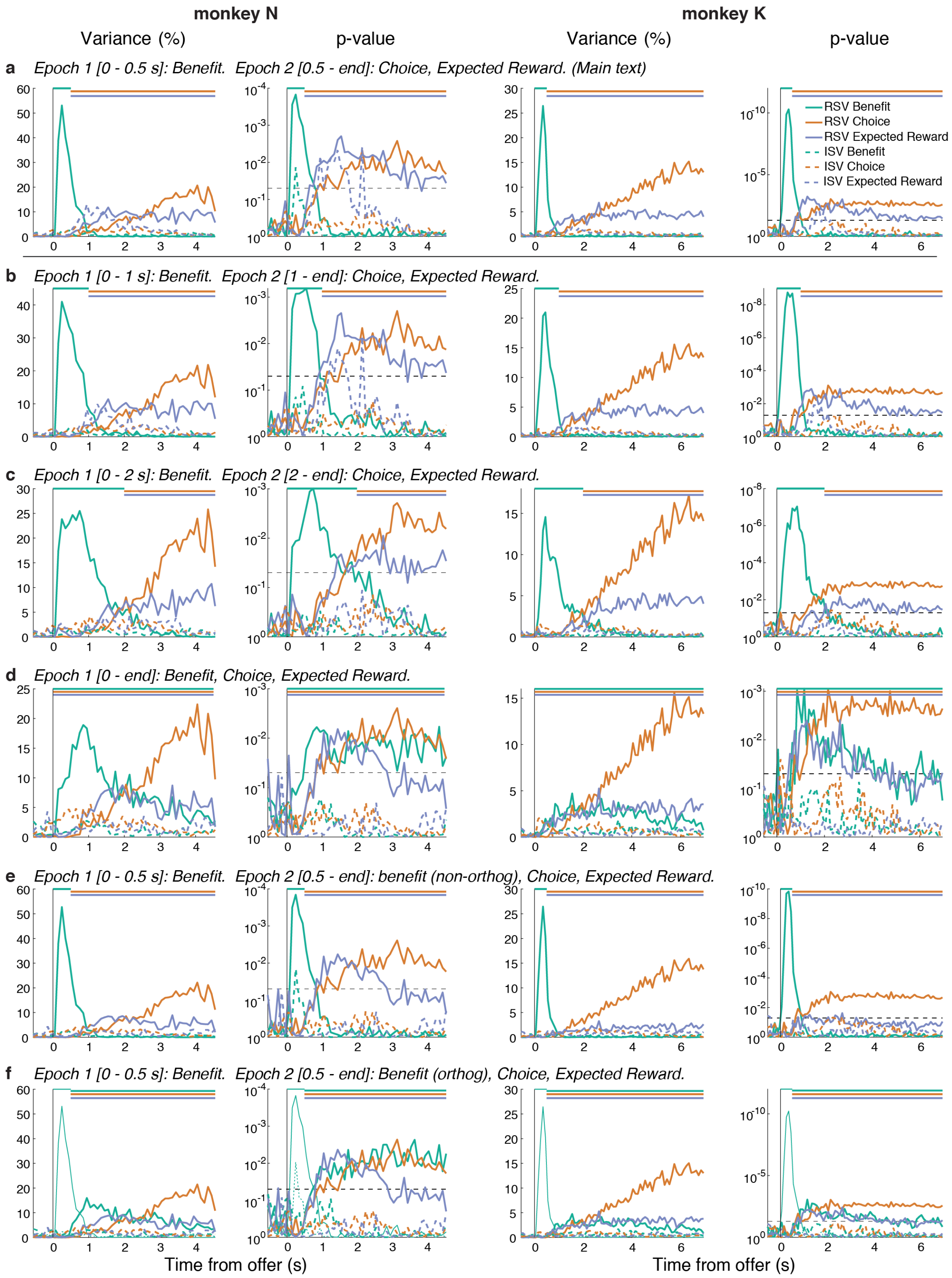

Supplementary Figure 12. Effect of temporal epoch on the sRAs.
(a-f) The relevant and irrelevant signal variance (RSV and ISV, solid and dashed curves, respectively) are shown in columns 1 and 3, with associated $\log_{10}$ p-values in columns 2 and 4, for BENEFIT (green), CHOICE

(orange), and EXPECTED REWARD (blue) as a function of time from the onset of the offer period for monkeys N (columns 1-2) and K (columns 3-4). P-values were one-sided, uncorrected, and derived empirically in comparison to random dimensions (see Methods); horizontal gray dashed line shows p = 0.05. Each row shows a separate implementation of oTDR with distinct temporal epochs for computing the sRAs (italicized headings). The colored horizontal bars at the top of each panel indicate the temporal epoch in which the color-matched sRA was computed.

(a) The oTDR implementation used in the main text is shown, with BENEFIT computed in Epoch 1 (corresponding to the offer period, 0 – 0.5 s) and CHOICE and EXPECTED REWARD computed in Epoch 2 (corresponding to the work period, 0.5 s to end of the trial). The sRAs explained significantly large portions of relevant variance throughout the epoch in which they were computed and little variance outside that epoch (solid curves). In addition, the ISV (i.e., variance unrelated to the targeted variables; dashed curves) was generally very small and statistically insignificant; this was true across the various implementations of oTDR and is not further discussed.

(b,c) We tested the impact on sRA sensitivity of extending the duration of Epoch 1 to either 1 s (b) or 2 s (c), while shortening Epoch 2 accordingly. Extending Epoch 1 reduced the RSV explained by BENEFIT (green curves) and, particularly for the 2 s duration (c), marginally increased the RSV for CHOICE (orange curves). These changes were predicted from the dRA similarity analysis: the encoding of benefit was stable during the 0.5 s offer period, then changed abruptly (Figure 5c-f, left panels). Therefore, targeting an sRA exclusively to the 0.5 s offer period would more fully capture the available signal, whereas extending the epoch in which the sRA was computed would necessarily dilute the specificity of the resulting sRA for the time-limited representation, as the sRA would now reflect a compromise between two, highly dissimilar representations (corresponding to the periods marked with red and blue brackets in Figure 5e,f, left panels). Likewise, because the representation of choice did not emerge until ~1.5 s (Figure 5c-f, middle panels), targeting the CHOICE sRA exclusively to this temporal epoch (as in (c)) resulted in a marginally greater RSV. Nonetheless, in the case of the choice signal, the representation was highly stable for an extended period and was without a competing representation prior to this period. Thus the CHOICE sRA suffered very little by including, as in the main analysis, the roughly 1 s period (0.5 s ~ 1.5 s) prior to the emergence of choice encoding. Despite these various quantitative effects of extending Epoch 1, the qualitative conclusions remained unchanged: the static sRAs of BENEFIT, CHOICE, and EXPECTED REWARD captured a high and statistically significant portion of variance related to their respective signals throughout the temporal epochs in which they were computed.

(d) Next we considered the case of having no *a priori* assumptions about the timing or stability of the task-relevant representations. In this implementation, we computed all three sRAs within a single epoch spanning the entire trial. As expected, the maximum explanatory power of the BENEFIT sRA decreased, but remained significant and spanned a longer portion of the trial, consistent with pooling over different representations of benefit during different phases of the trial, as discussed above. The RSV for CHOICE was largely unaffected by use of a single temporal epoch, showing how oTDR is generally robust to inclusion of periods of non-coding activity (i.e., before 1.5 s). However, the RSV for EXPECTED REWARD was diminished, particularly in the last 1 ~ 2 s of the trial, where it dropped below the significance threshold. Note that in the single-epoch model (d), EXPECTED REWARD was forced to compete with BENEFIT to explain variance during a time when the two signals were correlated (see Supplementary Figure 24a,b, middle panels, showing similar dRAs for benefit and expected reward late in the trial). Therefore, the decrease in RSV for EXPECTED REWARD was likely due to the inclusion of the temporally overlapping benefit regressor during this later period, rather than due to the expansion of the epoch in which EXPECTED REWARD was computed (i.e., starting at 0 s instead of 0.5 s). This conclusion was further supported by the next two analyses (e,f)—by including a separate benefit regressor in Epoch 2, while maintaining the original duration of Epoch 2, the RSV for EXPECTED REWARD decreased compared to the original analysis (a).

The single-epoch results (d) further justify computing the sRAs within time-limited epochs, in addition to our *a priori* rationale (see Methods) and relevant periods of stability in the dRA analysis (Figure 5). Nonetheless, even with minimal assumptions (i.e., computing all sRAs in a single, trial-spanning epoch), oTDR discovered statistically sensitive and specific representations of the task-relevant variables.

Thus far, we have specified oTDR to assume that each task-relevant variable was represented in one and only one temporal epoch. However, in the general form of the objective function (Methods, Equation (6)), one may assume that any arbitrary set of variables was encoded in one or more of any arbitrary number of temporal epochs. Moreover, one may assume that any arbitrary subset of variable-by-epoch

representations were orthogonal with one another, while making no orthogonality assumptions about the remaining representations. In the final two implementations, we demonstrated this flexibility of oTDR as applied to the current dataset.

As discussed, benefit was encoded by at least two distinct representations: during the offer (0 – 0.5 s) and after the offer during the work period (> 0.5 s) (Figure 5c-f, left panels, colored brackets). Here we use oTDR to explore this latter representation for two purposes.

First, one may want to control for the overlap between benefit and expected reward encoding during Epoch 2 (as discussed above and shown in Supplementary Figure 24a,b, middle panels). In this case, one is *not* interested in the read-out of the mid-trial benefit signal, only controlling for its impact. In oTDR, we assume that sRAs are orthogonal because this guarantees that the readout (both for the experimenter and downstream circuit) is independent between sRAs. However, in the case of only controlling for the mid-trial benefit signal, we do not require a readout for mid-trial benefit and thus do not orthogonalize its representation. In **(e)**, we show the results of a model that included a non-orthogonalized regressor for benefit in Epoch 2, in addition to the orthogonalized regressors that gave rise to the sRAs from the main text (i.e., BENEFIT in Epoch 1 and CHOICE and EXPECTED REWARD in Epoch 2). (Note that because coefficients from the mid-trial benefit regressor were not orthogonalized with respect to the sRAs, any variance-related metrics, i.e., RSV and ISV, for mid-trial benefit would not be independent of the sRAs and thus were not computed or plotted.) We found that including the mid-trial benefit regressor had virtually no impact on the BENEFIT and CHOICE sRAs, but reduced the explanatory power and statistical significance of EXPECTED REWARD, particularly late in the trial for monkey N and throughout the trial for monkey K. As discussed above, this was likely due to the similarity of benefit and expected reward representations late in the trial (Supplementary Figure 24a,b). By removing the orthogonality constraint, the mid-trial benefit representation would have an advantage over the EXPECTED REWARD sRA, which had to remain orthogonal with the other sRAs, in competing for shared variance. Thus, this implementation of oTDR was the most conservative in addressing the specific concern of misattribution of variance related to benefit late in the trial.

Second, one may have a functional interest in the mid-trial benefit representation, such as considering how the early and mid-trial benefit representations maybe readout independently by downstream circuits. To test this hypothesis, we implemented oTDR using the same regressors as in (e), only now we assumed that the mid-trial benefit representation *was* orthogonal to the other sRAs (and likewise discovered a dedicated MID-TRIAL BENEFIT sRA). As shown in **(f)**, this model discovered sRAs that were virtually identical to those from the previous non-orthogonalized model (e), except that now the RSV for EXPECTED REWARD in monkey K was both larger and significant for the first 3.5 s of the work period. This rescue of EXPECTED REWARD was due to the fact that the competing mid-trial benefit regressor now had to abide the orthogonality constraint along with the other sRAs. Note that this did not confer any special advantage for EXPECTED REWARD to explain this variance; that is, if the mid-trial variance were more related to offer size, then it would be explained by the MID-TRIAL BENEFIT sRA, leaving EXPECTED REWARD to explain little to no variance. Finally, we now also observe the MID-TRIAL BENEFIT sRA (f, *thick* green curve), which explained a significant portion of benefit information from just after the offer (0.5 s) to the middle (monkey K) or end (monkey N) of the trial, as anticipated from the dRA analysis (Figure 5c-f, left panels). The original BENEFIT sRA (computed during 0 – 0.5 s) is now shown by the *thin* green curve.

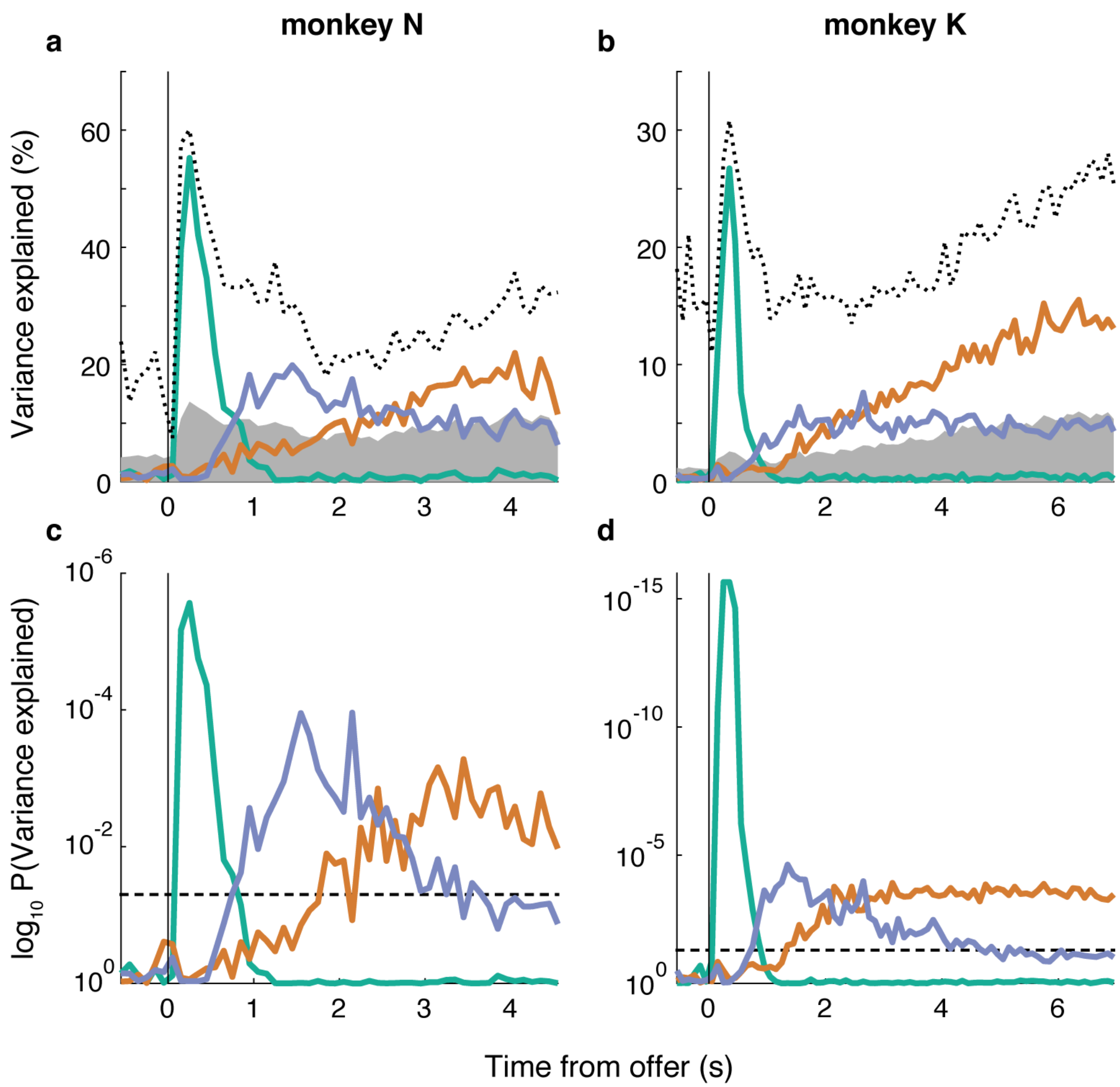

Supplementary Figure 13. Variance explained by low-dimensional representations.
Figure 4c,d plotted the extent of relevant and irrelevant signal variance, which were functions of the overall variance explained, now shown here for completeness. **(a,b)** The percentage of variance explained (i.e., cross-condition variance of the projection onto a given sRA normalized by the cross-condition variance across all dimensions) by the sRAs of BENEFIT (green curve), CHOICE (orange curve), and EXPECTED REWARD (blue curve) is plotted as a function of time from the onset of the offer period (black vertical line) for monkeys N (a) and K (b). The area of gray shading represents the variance explained by 95% of random vectors reflecting the dimensionality of the data. To estimate the upper-bound for variance explained any single, time-varying dimension (note that the sRAs were static, in contrast), we performed principal component analysis independently at each time bin and plotted the variance explained by the top component (black dotted curve). **(c,d)** We computed the one-sided, uncorrected probability, P(V), of observing the percent variance explained by chance based on the distribution of variance explained by the set of random vectors. We plotted $\log_{10}$ P(V) for each sRA (colors as in a,b) as a function of time from the onset of the offer period (black vertical line) for monkeys N (c) and K (d). For monkey K, P(V) at times 0.25 and 0.35 s was less than machine precision, and the values were replaced with the precision floor value. The threshold P = 0.05 is plotted (horizontal dashed line) for reference.

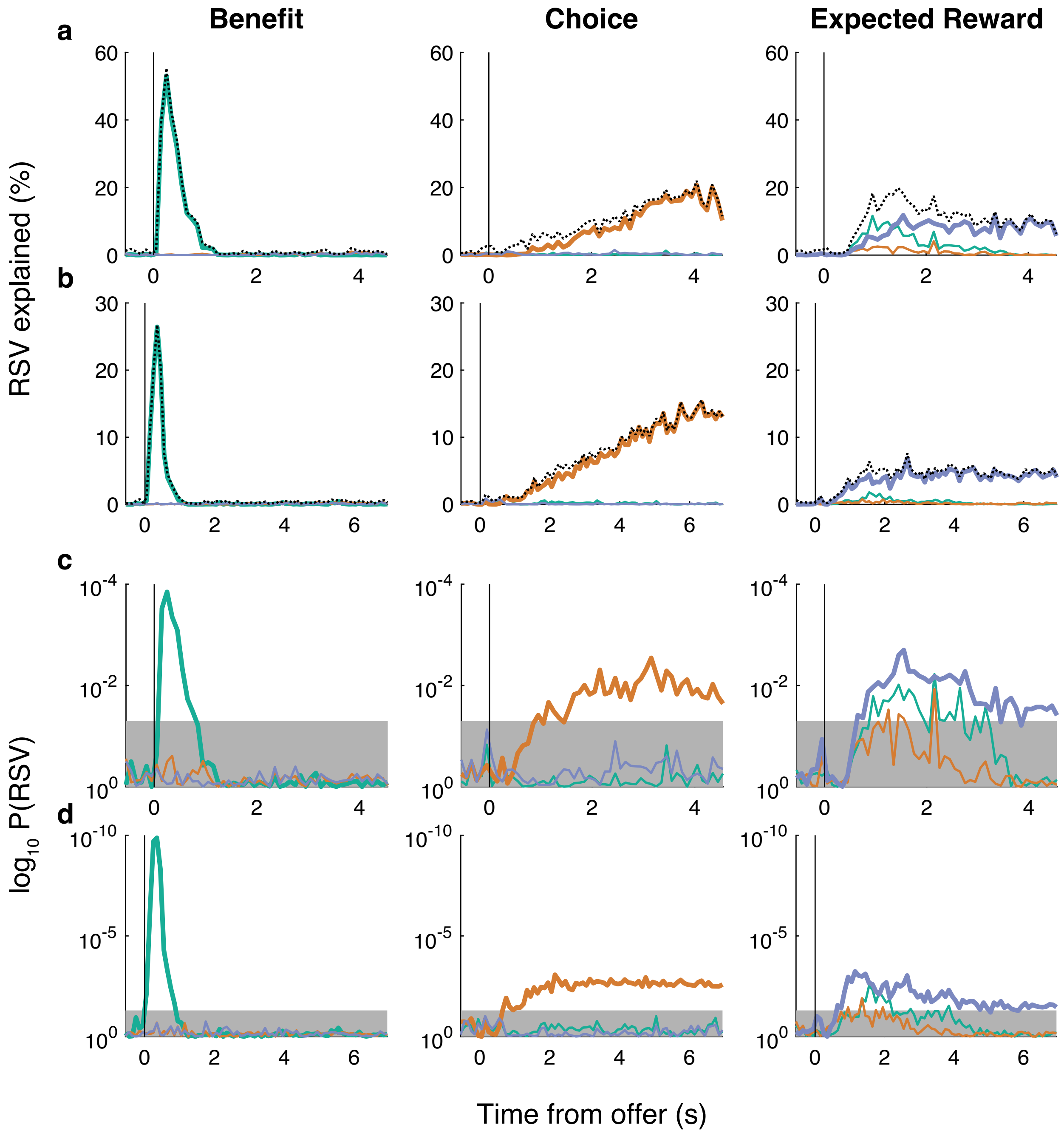

Supplementary Figure 14. Specificity of low-dimensional representations.
We were interested in the specificity of the low-dimensional representations, that is, the extent to which the sRAs explained variance related to the targeted variable of interest and not related to off-target variables. For each sRA, we computed the portion of variance explained that was related to *each* of the three variables (see Methods, on- and off-target RSV), thereby generating three RSV values for each sRA at each time bin. **(a,b)** The RSV explained by the sRAs of BENEFIT (left panel), CHOICE (middle panel), and EXPECTED REWARD (right panel) is plotted (solid colored curves) as a function of time from the onset of the offer period (vertical black line) for monkeys N (a) and K (b). Colors refer to the variable with respect to which the RSV was computed: benefit (green), choice (orange), and expected reward (blue). The on-target RSV is shown in thick curves (e.g., the variance explained by BENEFIT that was related to the benefit variable is shown in left panel in thick green curve) and recapitulates Figure 4c,d. Off-target RSV is shown in thin curves.

The overall variance explained (V; dotted black curve) recapitulates the curves in Supplementary Figure 13a,b and served as an upper-bound for RSV explained. When the difference between V and RSV was zero, the sRA was perfectly specific to the on-target variable. Any lack of specificity of the sRA (i.e., irrelevant signal variance; Figure 4c,d, dashed curves) must have arisen within the difference between V and RSV. The RSV with respect to the off-target variables depended on the correlations between the variables themselves, which ranged as high as Pearson's r = 0.86 for expected reward and benefit for monkey K. For an intuition, if the correlation between variables A and B were r = 1, then the RSV with respect to A and B would be equal. To address the correlation between variables, we used semi-partial correlation to compute the off-target RSV with respect to off-target variable *q* that would be expected given the correlation r_{kq} between *q* and on-target variable *k* (see Methods). The off-target RSV (thin colored curves) is plotted in (a,b), where the color indicates the off-target variable *q* with respect to which the RSV was computed.

(c,d) The probability (one-sided, uncorrected) of observing on- and off-target RSV (thick and thin curves, respectively) by chance is plotted for monkeys N (c) and K (d), where panels and colors are as in (a,b). Probabilities were derived from empirical null distributions of on- and off-target RSV (see Methods). The area of gray shading represents P(RSV) > 0.05.

In general, on-target RSV closely approached the upper-bound V, indicating high-specificity of the sRAs. In addition, the probability of observing the on-target RSV by chance was less than 0.05 during key task-relevant periods (discussed in main text). The off-target RSV was generally very small and below chance levels. However, for both animals, EXPECTED REWARD explained significant variance related to benefit from 1 ~2 s after the offer (thin green curve, right panels). This leak of variance related to benefit onto EXPECTED REWARD suggested that the transformation from encoding of pure benefit to encoding of benefit conditioned on choice (i.e., expected reward) was not instantaneous, but rather evolved gradually during and just after the period when the animals rendered most of their choices, as can be observed in the trial-average responses projected onto EXPECTED REWARD (Figure 4a,b, bottom panels), which separate rapidly by offer size in the first second after the offer and prior to when they additionally separate by choice. Of note, the lack of benefit information explained by the BENEFIT sRA during this time (thick green curve, left panels) indicated that the residual benefit information during the transformation was in a direction orthogonal to BENEFIT, consistent with the reduced sensitivity of BENEFIT during this time (see Supplementary Figure 15). The cross-talk between benefit and expected reward information was distinct from the leak of benefit information onto CHOICE mid-trial, as also observed in Figure 4a,b (middle panels) and discussed in the main text.

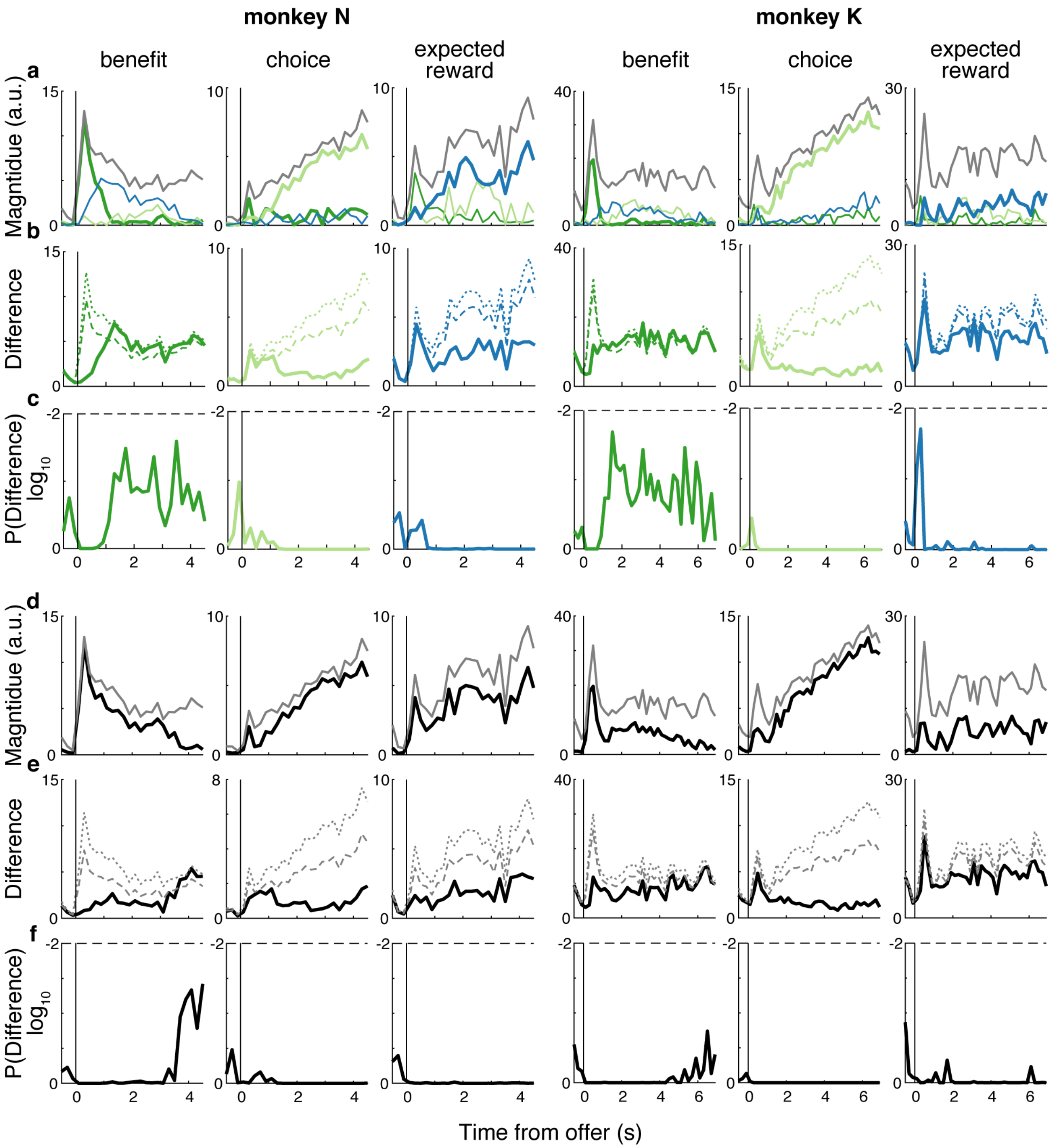

Supplementary Figure 15. Sensitivity of low-dimensional representations.

We were interested in the sensitivity of the static regression axes (sRAs) to the encoding of the task-relevant variables at the individual-unit level. That is, how much of the encoded information available in the population at large was captured by the low-dimensional, static space spanned by the sRAs? We reasoned that the dynamic regression axes (dRAs) discovered the best linear representation of the variables at each time bin, and thus could be used to quantify the available momentary information. However, the approach most analogous to our prior analyses (i.e., projecting the neural data onto the RAs and measuring the variance explained) was not possible for the dRAs because the axes were non-orthogongal, and thus the projection onto a given dRA would necessarily contain off-target variance related to the other variables. Instead, we relied on the fact that the vector magnitude $||dRA_k(t)||$, as defined in the high-dimensional space and prior to vector normalization, was proportional to the degree of

neural representation of variable *k* at time *t* relative to the other variables and time bins (recall that predictors were scaled and centered and neural responses were mean-centered). In addition, we reasoned that the extent to which $dRA_k(t)$ aligned with each sRA, or with the low-dimensional space spanned by all three sRAs, was proportional to the sensitivity of the sRA(s) for variable *k* at time *t*. Therefore, we projected $dRA_k(t)$ onto each sRA_i ($i,k \in$ {benefit, choice, expected reward}) and measured the resulting magnitude, $z_{k,i}(t)$. When $z_{k,i}(t)$ approached $||dRA_k(t)||$, then sRA_i captured a high proportion of the available information about variable *i* at time *t*, i.e., it was sensitive to variable *i*. We measured $z_{k,i}(t)$ both for the on-target variable (i.e., $i = k$), as well as for the off-target variables (i.e., $i \neq k$), which indicated the extent to which sRA_i was sensitive to the other variables.

(a) We plotted $z_{k,i}(t)$ as a function of time *t* from the onset of the offer period (vertical black line) for each $dRA_k(t)$. Within the left (monkey N) or right (monkey K) set of three columns, the left, middle, and right panels correspond to *k* = benefit, choice, and expected reward, respectively. Line color corresponds to the sRA_i onto which the dRA was projected (sRAs of BENEFIT, CHOICE, and EXPECTED REWARD plotted in green, orange, and blue, respectively). Thick or thin colored lines indicate the on- or off-target variable (i.e., $k = i$ or $i \neq k$), respectively. For reference, the magnitude of $dRA_k(t)$ in the high dimensional space—an upper-bound for $z_{k,i}(t)$—is shown in gray curves.

Qualitatively, it appeared that the sRAs captured a high proportion of available information about their targeted variable (i.e., thick colored curves approached gray curves), at least for portions of the trial. However, we sought to quantify the extent to which the sRAs did *not* capture the available information and to compare this extent to that expected for an arbitrary trio of static dimensions. As such we computed the difference $d_{k,i}(t) = ||dRA_k(t)|| - z_{k,i}(t)$, or the population encoding of variable *k* *un*detected by sRA_i. We developed a statistical null model for $d_{k,i}(t)$ by generating *S* random sets of three orthogonal vectors (i.e., null sRAs) biased to the dimensionality of the data using the same biased sampling method as for single vectors (Methods). We then projected $dRA_k(t)$ onto each null sRA from a given random set, computed the null magnitude $\hat{z}_{k,i}(t)$ and corresponding null difference $\hat{d}_{k,i}(t)$, and compiled across random sets so as to ultimately generate a null distribution $\hat{\mathbf{d}}_{k,i}(t)$ of undetected information. Because the null sRAs were generated without regards to the variables of interest, there was no *a priori* pairing between $dRA_k(t)$ and a given null sRA. Therefore, we designated the null sRA most aligned to the dimension of greatest variance in the data as the "on-target" null sRA and used this null sRA to compute $\hat{d}_{k,i}(t)$ for $i = k$. (This designation was the most conservative approach, i.e., produced the smallest null differences $\hat{d}$ and thus was most likely to identify a given observed difference d as statistically large). The undetected information about off-target variables (i.e., $\hat{d}_{k,i}(t)$ for $i \neq k$) was of less interest, and so the remaining two null sRAs were assigned arbitrarily to the remaining two "off-target" variables. (However, below we summed $\hat{d}_{k,i}(t)$ across all null sRAs *i* for computing $\hat{D}_{k,i}(t)$.)

(b) The difference $d_{k,i=k}(t)$ is shown for the on-target variables in thick curves following the same color and column conventions as in (a). The 50th and 99th percentiles of the corresponding null distribution $\hat{\mathbf{d}}_{k,i=k}(t)$ are provided for reference (dashed and dotted curves, respectively).

Finally, we computed the probability $p_{k,i}(t)$ of obtaining the observed $d_{k,i}(t)$ or greater by chance as the upper-tail of $\hat{\mathbf{d}}_{k,i}(t)$ from $d_{k,i}(t)$ to ∞. (Note that we integrated $\hat{\mathbf{d}}_{k,i}(t)$ numerically, and thus p was lower-bounded at $1/S$.) When $p_{k,i}(t)$ was sufficiently large, we concluded that the amount of encoded information undetected by sRA_i was no more than expected by chance.

(c) We plotted $\log_{10} p_{k,i=k}(t)$ for the on-target variable (one-sided, uncorrected, derived empirically in comparison to null distribution $\hat{\mathbf{d}}_{k,i}(t)$, described above) using the same color and column conventions as in (a) and labeled the threshold $p = 0.01$ (horizontal dashed line) for reference.

We observed that CHOICE and EXPECTED REWARD generally detected a consistent proportion of available population encoding (i.e., consistent spacing between thick colored and gray curves, (a)), suggesting that the observed dynamics of RSV(*t*) or V(*t*) (see Figure 4c,d or Supplementary Figure 13a, respectively) were due to changes in the magnitude of the dynamic representation (i.e., $||dRA_k(t)||$), not due to changes in the direction of representation. Conversely, for BENEFIT, we observed both a decrease in the dynamic representation magnitude with time (i.e., decrease in gray curve, (a)) *and* a decrease in the proportion of detected population representation (i.e., increase in difference $d_{k,i}(t)$, (b)), suggesting that the representation of benefit was dynamic both in magnitude and direction (i.e., contributions of individual units). For all sRAs at all times, the magnitude of *un*detected encoding of the on-target variable

(i.e., $d_{k,i=k}(t)$) was less than expected by 99% of random sRAs (c). In other words, the high sensitivity of a given sRA at key task-relevant times (e.g., early for BENEFIT, late for CHOICE) did not compromise the ability of the sRA to detect the encoding of that variable at other times in the trial, as compared to any arbitrary trio of static dimensions.

(d) We were also interested in how well the entire static three-dimensional (3-D) subspace spanned by the sRAs captured the dynamic representations. As such, we measured the magnitude $Z_k(t)$ of the projection of $dRA_k(t)$ into the 3-D space, taken as the vector norm $\sqrt{\sum_i z_{k,i}(t)^2}$ across sRAs *i* (recall that the sRAs were orthogonal). We plotted $Z_k(t)$ (black curve) as a function of time and included the magnitude of $dRA_k(t)$ in the high-dimensional space (gray curve) as an upper-bound for $Z_k(t)$.

Like for the individual sRAs, we measured the extent of information *un*captured by the 3-D space as the difference, $D_k(t) = ||dRA_k(t)|| - Z_k(t)$, between the magnitudes of the dRA in the high-D and 3-D spaces. Likewise, to generate a null model, we computed the difference, $\hat{D}_k(t)$, between $||dRA_k(t)||$ and the magnitude $\hat{Z}_k(t)$ of $dRA_k(t)$ projected into the subspace of three orthogonal random vectors

(e) The difference $D_k(t)$ (thick solid black curves) and 50th and 99th percentiles of $\widehat{\mathbf{D}}_k(t)$ (dashed and dotted gray curves, respectively) are plotted. As above, we computed the probability $P_k(t)$ of obtaining $D_k(t)$ or greater by chance as the upper-tail of $\widehat{\mathbf{D}}_k(t)$ from $D_k(t)$ to ∞.

(f) We plotted $\log_{10} P_k(t)$ (one-sided, uncorrected, derived empirically in comparison to null distribution $\widehat{\mathbf{D}}_k(t)$, described above) and labeled the threshold P = 0.01 (horizontal dashed line).

The static, 3-D space captured a high proportion of the population encoding of the task-relevant variables (black curves are close to gray curves, (d)). Exceptions tended to occur during periods of lower absolute representation (i.e., smaller $||dRA_k(t)||$), such as the representation of benefit or choice late or early in the trial, respectively. However, we noted that the proportion of available expected reward representation captured by the low-dimensional space was considerably lower than for the other task-relevant variables, including during periods of relatively large dynamic representation. Statistically, for all variables at all times, the magnitude of *un*detected encoding (i.e., $D_k(t)$) was less than expected by 99% of random sets of sRAs (f). In other words, the static low-dimensional space, in addition to representing the variables of interest at key times, was no worse than chance at capturing the available population encoding of the variables across all times in the trial.

Though not its intention, the present analysis afforded an alternative measure of specificity to complement Supplementary Figure 14. The extent of information about a given variable captured by the low-dimensional space was generally captured by the sRA specific to that variable (i.e., thick curves greater than thin curves, (a)). An exception was the dynamic representation of benefit (a, left panels), which was captured by BENEFIT early in the trial but by CHOICE and EXPECTED REWARD in the mid-to-late trial. This cross-talk reflected the co-alignment between benefit and choice or expected reward representations mid-trial (see Supplementary Figure 24 for alignment between dRAs).

Principal component analysis

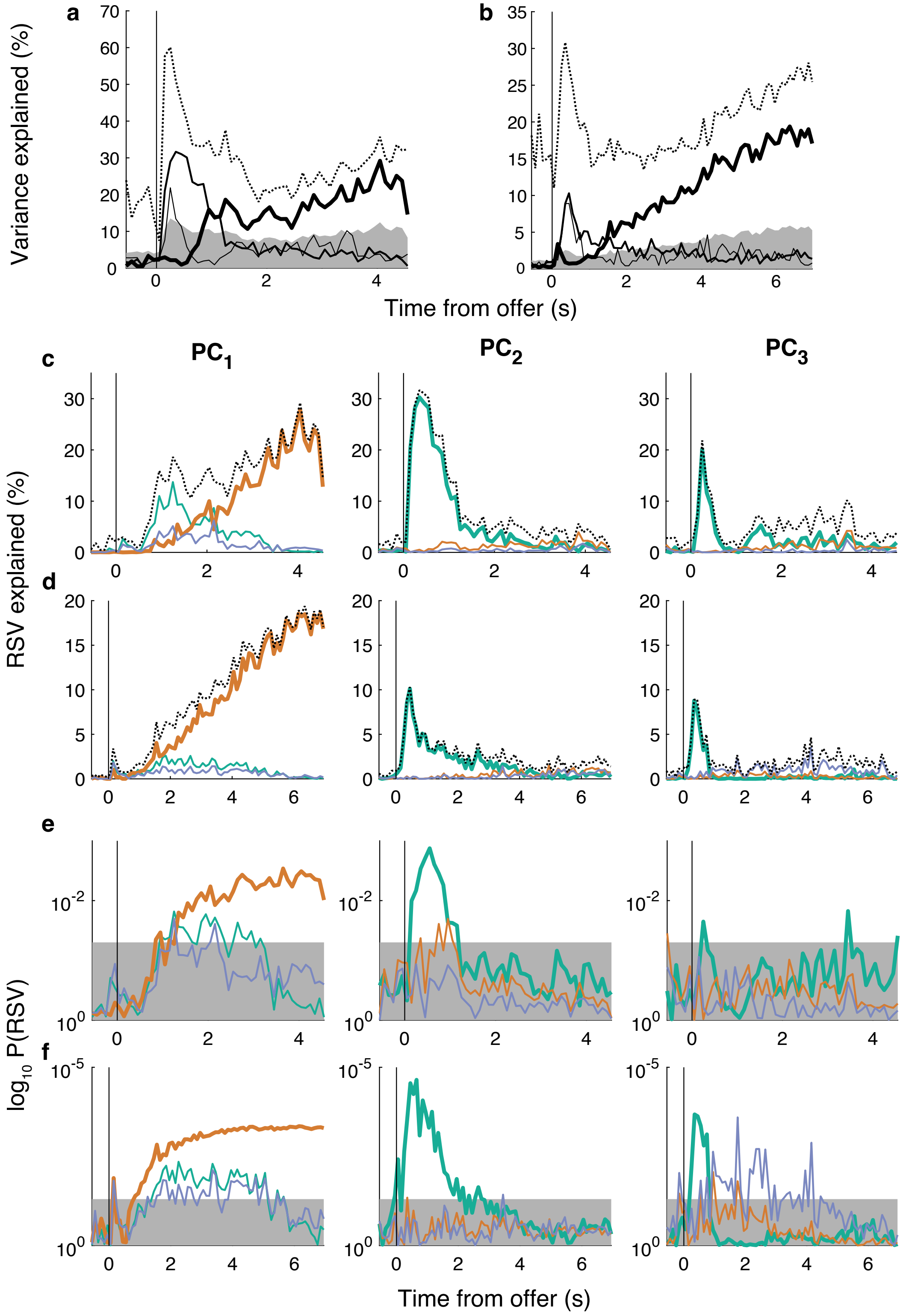

Supplementary Figure 16. Variance explained by principal components.
Principal components analysis (PCA) provided an alternative, traditional approach to dimensionality reduction. We compared the low-dimensional space spanned by the sRAs to that spanned by the top

three principal components (PCs), as discovered by PCA on variance across time and conditions in the high-dimensional space. As with oTDR, the analysis was performed on z-transformed, common condition-subtracted responses, $\bar{\mathbf{R}}$, which were reshaped to matrix form with rows (dimensions) corresponding to units and columns (observations) corresponding to concatenation of time bins and conditions.

(a,b) Variance explained by PC_1 (thick solid curve), PC_2 (medium solid curve), and PC_3 (thin solid curve) is shown as a function of time from the onset of the offer period (black vertical line) for monkeys N (a) and K (b). The area of gray shading represents the variance explained by 95% of random vectors (see Methods), and the black dotted curve represents the variance explained by the top PC from a separate analysis in which we performed PCA independently at each time bin to estimate the upper-bound for the variance explained by any single, time-varying dimension. Compared to the sRAs (Supplementary Figure 13), the PCs explained less peak variance early in the trial and moderately greater variance toward the end of the trial.

However, the variance explained by the top PCs was not necessarily related to the variables of interest. To address the specificity of the PCs, we computed the relevant signal variance (RSV) explained by each PC with respect to the variables of interest. Because the PCs were not targeted to specific variables *a priori*, we designated as "on-target" the variable for which the cumulative RSV (summed across time bins) was greatest: choice for PC_1 and benefit for PC_2 and PC_3

(c,d) The on- and off-target RSV (thick and thin solid curves, respectively) explained by PC_1 (left panel), PC_2 (middle panel), and PC_3 (right panel) is plotted as a function of time from the onset of the offer period (vertical black line) for monkeys N (c) and K (d). Colors refer to the task-relevant variables with respect to which RSV was computed: benefit (green), choice (orange), and expected reward (blue). The overall variance (V) explained by each PC (thin black dotted curve) recapitulates the solid curves in (a,b) and served as an upper-bound for RSV. Any lack of specificity of a given PC for a given variable arose within the difference between V and on-target RSV. We quantified the portion of variance related to the "off-target" variables (i.e., the two variables not designated as on-target) as the off-target RSV, the computation of which controlled for the correlation between on- and off-target variables (see Methods and Supplementary Figure 14).

(e,f) The $\log_{10}$ probability (one-sided, uncorrected) of observing on- and off-target RSV (thick and thin curves, respectively) by chance for monkeys N (c) and K (d) were derived from empirical null distributions of on- and off-target RSV (see Methods), where panels and colors are as in (c,d). The area of gray shading represents $p > 0.05$.

Compared to the sRAs (Supplementary Figure 14), the top PCs were less specific to any given variable of interest (i.e., greater difference between V and on-target RSV, (c,d)). Moreover, the variance explained by the PCs that was *unrelated* to the on-target variable was not merely irrelevant to the task, but rather was significantly related to the off-target variables (e,f), for example: PC_1 explained significant RSV with respect to choice (primarily) but also benefit and expected reward (left panels, thin green and blue curves), PC_2 explained significant RSV with respect to benefit (primarily) but also choice for monkey N (middle panel, thin orange curves), and PC_3 explained significant RSV with respect to benefit (primarily) but also expected reward for monkey K (right panel, thin blue curves). Finally, the sensitivity for a given variable of interest was spread between PCs. In particular, benefit was represented significantly and most greatly by PC_2 and PC_3, rather than consolidated onto a single dimension, as by the BENEFIT sRA. In summary, compared to the sRAs, any given PC was less sensitive and specific to a particular variable of interest, and thus, as a whole, the set of PCs less well separated the task-relevant signals into low-dimensional representations specific to each variable.

Common condition response

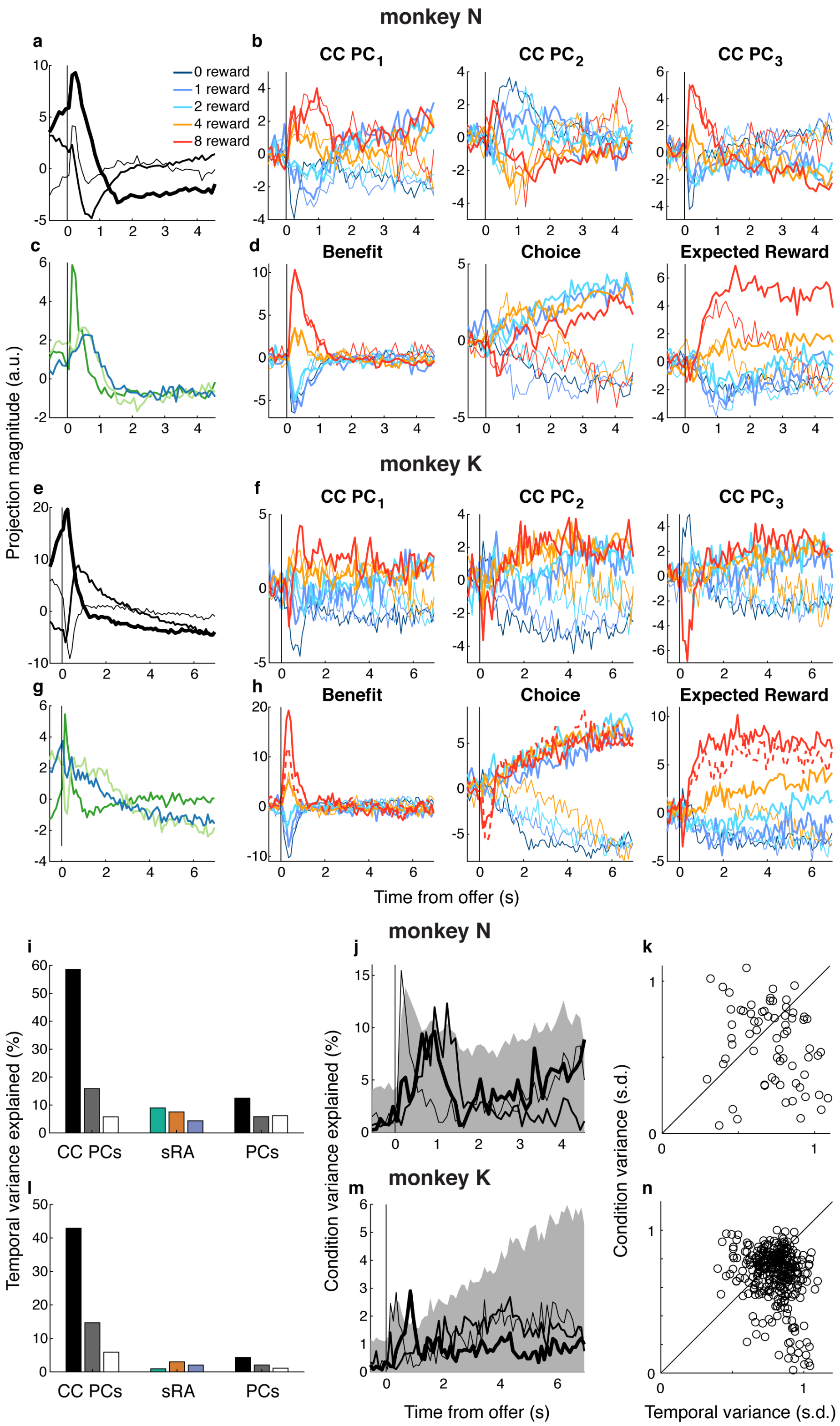

Supplementary Figure 17. Common condition response.
For monkeys N (a-d, i-k) and K (e-h, l-n), we examined the common-condition response (CC), i.e., the mean response across conditions taken at each time bin, and compared the CC to the condition-specific,

mean-subtracted response $\bar{\mathbf{R}}$. In particular, we were interested in identifying the subspace that captured the CC response and in measuring its overlap with the subspace that captured the task-relevant variables, particularly since the CC response is often a primary source of variance, which a downstream readout must distinguish in order to decode the task-relevant signals. We performed principal component analysis (PCA) on the CC (units x time bins), thus discovering the top three principal components (CC PCs) that captured the greatest temporal variance (i.e., variance across time bins). The projection of the CC onto a given dimension reflected the *activity* of that dimension (as in Figure 4a,b).

(a,e) The CC activity of the common-condition PCs—CC PC_1 (thick curve), CC PC_2 (medium curve) and CC PC_3 (thin curve)—and **(c,g)** CC activity of the sRAs—BENEFIT (green curve), CHOICE (orange curve), and EXPECTED REWARD (blue curve)—are shown as a function of time from the onset of the offer period (vertical line). Likewise, we projected the condition-*specific* response $\bar{\mathbf{R}}$ onto **(b,f)** CC PCs 1, 2 or 3 and **(d,h)** sRAs BENEFIT, CHOICE, or EXPECTED REWARD (left, middle, or right panels, respectively), plotted for each combination of offer size (colors, see legend) and choice (accept or reject choices in thick or thin curves, respectively).The magnitudes of the projections are in arbitrary units related to the *z*-transformed firing rates but are comparable between dimensions (i.e., CC PCs vs. sRAs) and animals. The projections of $\bar{\mathbf{R}}$ onto the sRAs (d,h) are identical to those in Figure 4a,b, and are recapitulated here to facilitate comparison to projections onto the CC PCs (b,f).

We examined the extent to which the various low-dimensional subspaces captured temporal variance. Qualitatively, the range of the CC response over time was greater when projected onto the CC PCs than onto the sRAs (a vs. c and e vs. g; note different scale of ordinates), suggesting the CC PCs captured greater temporal variance than the sRAs. **(i,l)** To test this explicitly, we measured the percent of temporal variance (bar height) explained by each of the CC PCs, sRAs, and, for completeness, the condition-sensitive principal components (PCs; discovered in matrix $\bar{\mathbf{R}}^{TC}$ and described in Supplementary Figure 16), where black, gray, and white bars refer to the first, second and third PC or CC PC, and green, orange and blue bars refer to the sRAs of BENEFIT, CHOICE, and EXPECTED REWARD, respectively. Indeed, the CC PCs explained markedly greater temporal variance than the PCs or sRAs.

In contrast, when projecting the condition-specific neural response (i.e., $\bar{\mathbf{R}}$) onto the various dimensions, the range of magnitudes for the CC PCs (b,f) was less than for the sRAs (d,h), suggesting the CC PCs captured less cross-condition variance (i.e., variance across conditions at each time bin) than the sRAs. In addition, the extent to which the CC PCs discriminated the variables of interest (i.e., orderly separation of projections by offer size and/or choice) was qualitatively less than for the sRAs. **(j,m)** Indeed, the cross-condition variance explained by CC PC_1 (thick curve), CC PC_2 (medium curve) and CC PC_3 (thin curve) was less than expected by 95% of random vectors reflecting the dimensionality of the data (gray shading) and less than explained by the sRAs (compare to Supplementary Figure 13a,b). (The present figure compares variance explained for individual dimensions across the three static subspaces; see Supplementary Figure 18 for comparison of the subspaces as a whole.)

Finally, we were interested in directly comparing the magnitudes of temporal and cross-condition variance in OFC. That is, were the condition-specific responses dwarfed by the time-varying, common-condition response, or were these two sources of variance comparable? Qualitatively, the range of magnitudes of the projections of the CC onto the CC PCs (a,e) was comparable to that of $\bar{\mathbf{R}}$ onto the sRAs (d,h), suggesting that condition-independent and condition-specific responses were of similar magnitude in OFC. To test this hypothesis more rigorously, for each unit *n*, we computed the temporal variance for each condition *c* from the *z*-transformed, trial-average responses $\bar{\bar{R}}_n(c,t)$, which contained the CC, and took the median variance across conditions. Separately, for each unit, we computed the cross-condition variance of $\bar{\bar{R}}_n(c,t)$ at each time bin and computed the median variance across time bins. **(k,n)** We plotted the median temporal (abscissa) and cross-condition (ordinate) variance for each unit (circles) in terms of standard deviations from the unit's mean response across all times and conditions. Points below the unity line (black diagonal) indicated greater temporal than cross-condition variance. The pairwise difference of temporal minus cross-condition variance was significantly greater than zero for the population (median = 0.10 or 0.07 and p = 0.0075 or $2.5 * 10^{-15}$ via two-sided Wilcoxon signed-rank test, monkey N or K, respectively), indicating greater temporal than condition variance. However, the extent of this difference was small: less than 0.1 s.d. on average and always less than 1 s.d. Thus, we concluded that OFC responses exhibited marginally but significantly greater temporal than condition variance.

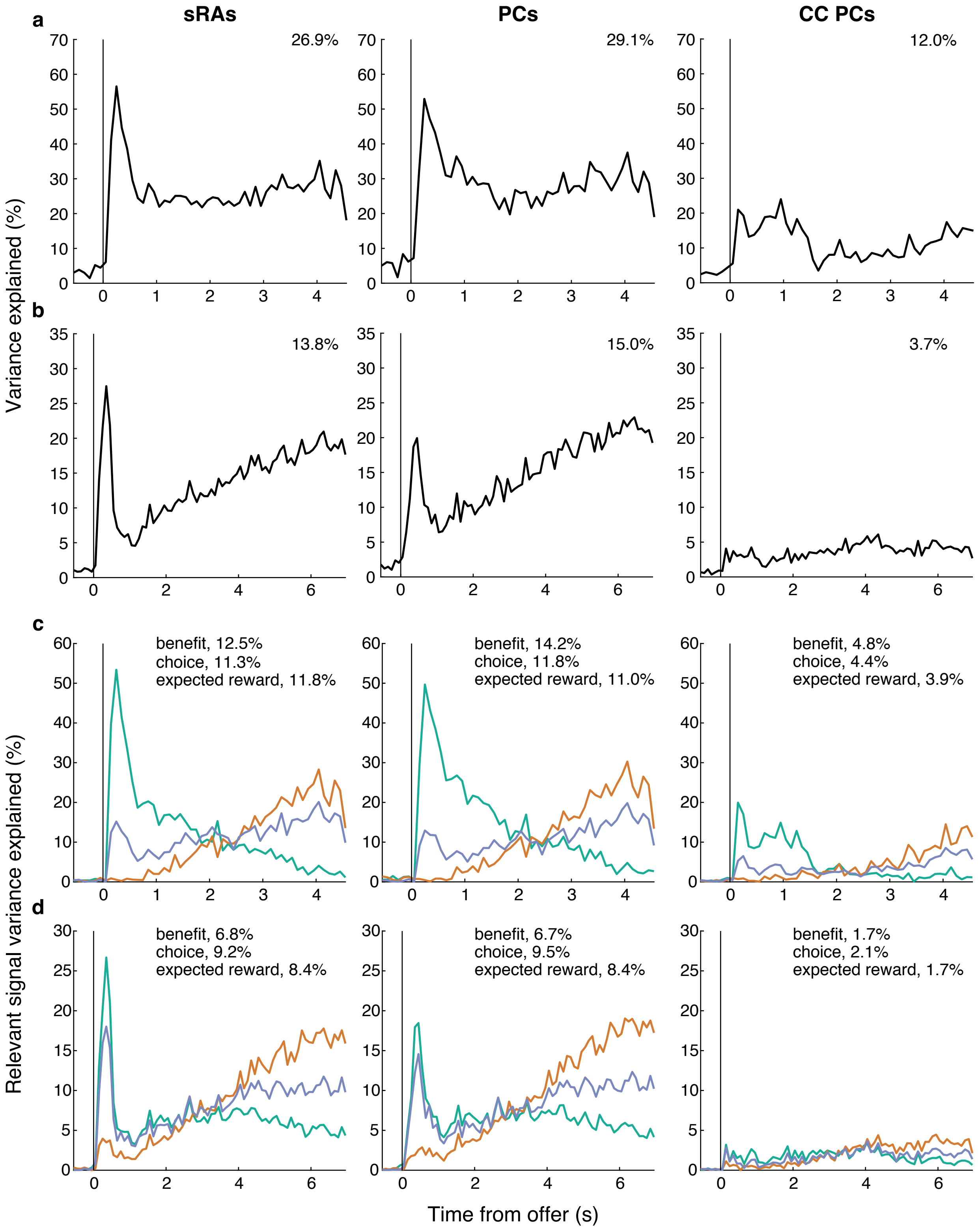

Supplementary Figure 18. Variance explained by 3-D subspaces.
We were interested in comparing the explanatory power of the previously discussed 3-dimensional subspaces—static regression axes (sRAs; left panels; see Figure 4 and Supplementary Figure 13), principal components (PCs; middle panels; see Supplementary Figure 16), and common-condition

principal components (CC PCs; right panels; see Supplementary Figure 17)—as a whole, instead of just their component dimensions examined separately.

(a,b) Cross-condition variance or **(c,d)** relevant signal variance (RSV) explained by a given 3-D subspace (as summed over the three contributing orthogonal dimensions) is plotted as a function of time from the onset of the offer period (vertical black line) for monkeys N (a,c) and K (b,d). The mean variance or RSV across time bins is indicated in the top right of each panel. For RSV, line color refers to the task-relevant variable with respect to which RSV was computed: benefit (green), choice (orange), and expected reward (blue).

In terms of variance (a,b), the PCs explained the greatest mean variance, as expected by the design of principal components analysis. However, the variance explained by the sRAs was nearly as great on average and greater at peak time bins, consistent with a high proportion of variance in the neural population as a whole being related to the task-relevant variables. In contrast, the subspace capturing temporal variance, as defined by the CC PCs, explained much less of the cross-condition variance, consistent with the temporal and cross-condition variances occupying largely non-overlapping subspaces (see Supplementary Figure 17).

In terms of RSV (c,d), the comparison between the sRAs and PCs was similar as for variance explained. However, the mean RSV explained by the sRAs was even closer to and at times exceeded the mean RSV explained by the PCs, consistent with the greater sensitivity of the sRAs to the task-relevant variables, despite the marginally greater overall variance explained by the PCs.

Note that we summed RSV across sRAs without controlling for correlations between the variables, in contrast to our prior analyses that used partial correlation to compute RSV for off-target variables (Supplementary Figure 14). As a result, at a given time, the total RSV across variables explained by the 3-D subspace (i.e., sum of the three colored lines in (c,d)) could exceed the variance explained by the same subspace (a,b). We emphasize that, for a given dimension, RSV was always bounded by variance explained, and likewise the sum of RSV for a given variable across dimensions was bounded by variance explained summed across those same dimensions.

In addition to comparing the variance explained by the various subspaces, we compared their overlap directly as measured by the alignment index (Methods), which ranged from 0 to 1. The overlap between the sRA and PC subspaces was high (alignment index = 0.84 or 0.65, monkey N or K, respectively), consistent with the OFC population being primarily modulated by the task-relevant variables. In contrast, the overlap between the sRA and CC PC subspaces was small (alignment index = 0.23 or 0.11, monkey N or K, respectively), consistent with the relative specificity of the CC PCs or sRAs for temporal or cross-condition variance, respectively.

Stability of dynamic representations

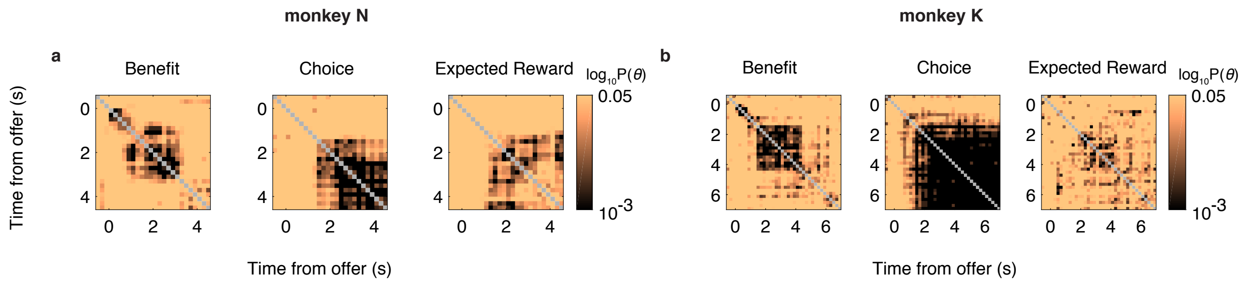

Supplementary Figure 19. Probability of pairwise similarity between dynamic representations. (a,b) The $\log_{10}$ probability of observing by chance the similarity θ between each pair of dynamic low-dimensional representations (dRAs; see Figure 5) is given by the pixel color (referencing right-sided color scale), and the times of the dRA pair (relative to the onset of the offer period) are given by the pixel's row and column positions for task-relevant variables of benefit (left column), choice (middle column), and expected reward (right column) for monkeys N (a) and K (b). The diagonal compares identical dRAs ($\theta = 0$, $p(\theta) = 1$) and is colored gray.

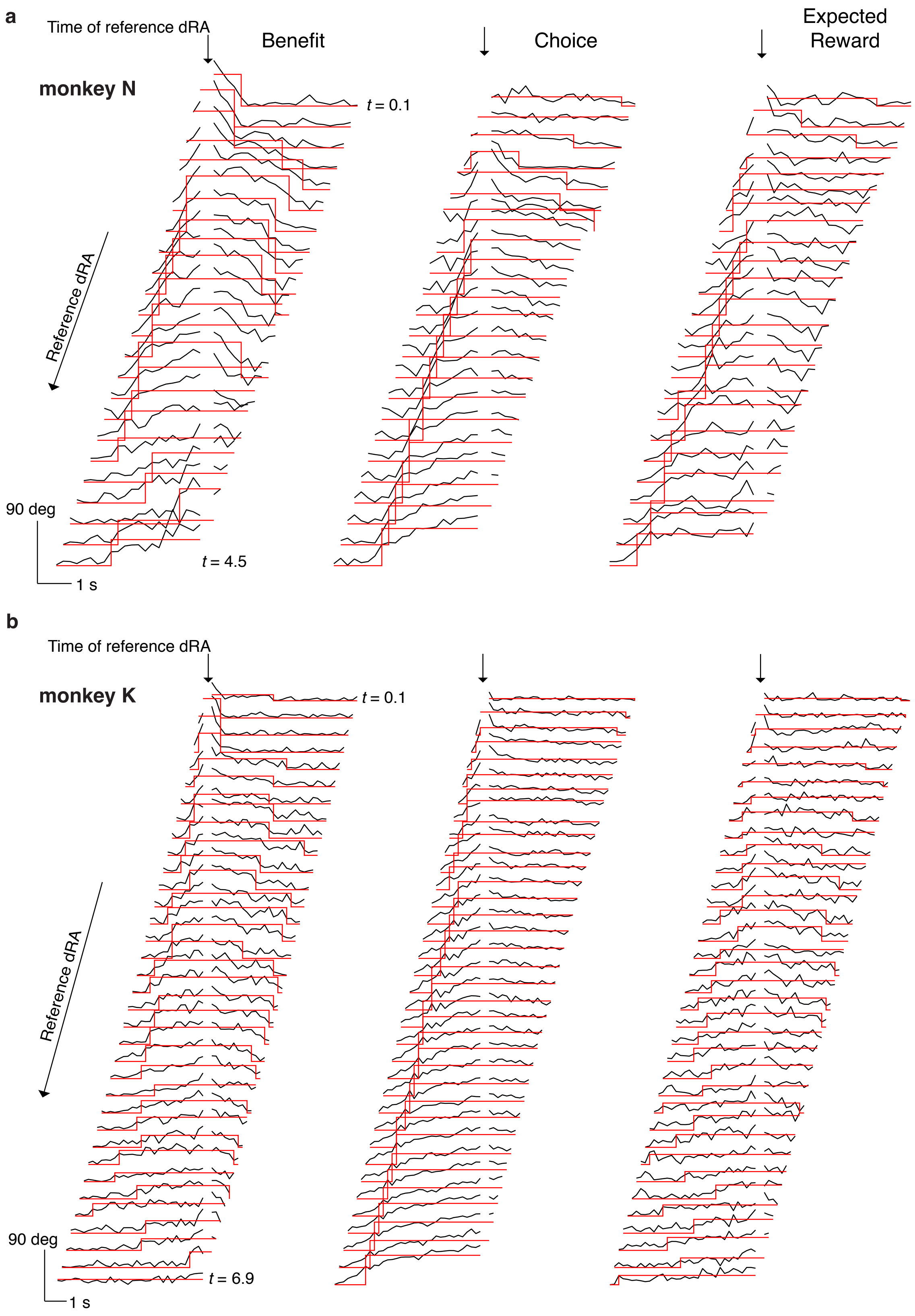

Supplementary Figure 20. Boxcar functions fit to angle between dynamic representations.
(a,b) The time-varying angle θ between pairs of dynamic low-dimensional representations (dRAs) for the task-relevant variables of benefit (left column), choice (middle column), and expected reward (right

column) are shown for monkeys N (a) and K (b). Each black trace indicates $90° - \theta$ (such that higher values correspond to greater similarity) between reference dRA(*t*), or dRA*, taken at time *t* and all other dRA(*t*+*dt*) taken at temporal offset *dt* relative to dRA*. The absolute trial time of dRA* is given by the row position of the black trace from early (top) to late (bottom) in the trial (center of time bin for first and last dRA* is labeled) and corresponds to the rows in Figure 5c,d. The spacing between black traces is arbitrary and for display purposes only. Black traces are aligned horizontally to the time of dRA* (downward arrow) and are redacted at this time (white space) since the comparison of dRA* with itself was necessary $90° - \theta = 90°$. Boxcar functions (red traces) were least-squares fit to the black traces, excluding the time of dRA* and times before the offer (i.e., excluding $t < 0$). The width of the boxcar function indicated the putative period of stability and the boxcar height indicated the magnitude of similarity, as depicted in Figure 5e,f. Horizontal and vertical scale bars indicate trial time and dRA similarity (i.e., $90° - \theta$), respectively.

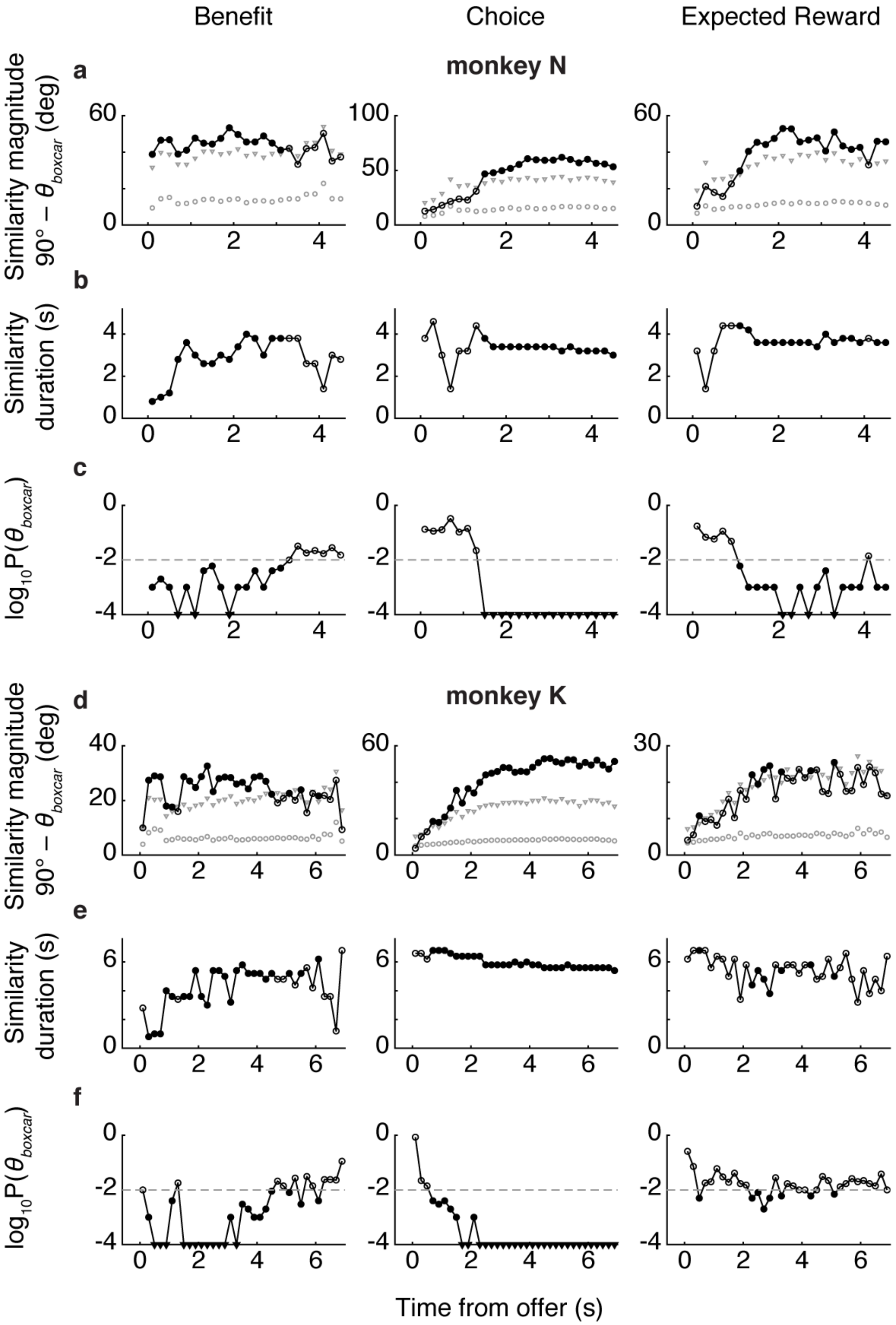

Supplementary Figure 21. Boxcar parameters and statistics.
The parameters and statistics of the boxcar functions fit to the angle between dynamic low-dimensional representations (dRAs) for task-relevant variables of benefit (left column), choice (middle column), and

expected reward (right column) are shown for monkeys N (a,b,c) and K (d,e,f). Boxcar fits were used to discover putative periods of stability with respect to a reference dRA* and adjacent dRAs (Supplementary Figure 20) and the associated parameters are shown as a function of the time of the reference dRA* relative to the onset of the offer period. **(a,d)** The boxcar height θ_{boxcar}, i.e., magnitude of similarity, is plotted (open or filled circles) as 90° – θ_{boxcar} (i.e., larger values indicate greater similarity). In all panels (a-f), filled circles indicate that the probability p of observing θ_{boxcar} by chance was $p(\theta_{boxcar}) < 0.01$, as computed from the distribution of chance angles $\hat{\theta}_{boxcar}$ between surrogate dRAs (Methods). The median (gray circles) and 99th-percentile (gray triangles) of the null distribution $\hat{\theta}_{boxcar}$ is shown for each time bin. **(b,e)** The boxcar width, i.e., duration of similarity, is plotted. **(c,f)** The $\log_{10}$ probability $p(\theta_{boxcar})$ (one-sided, uncorrected) was derived empirically in comparison to the distribution of chance angles $\hat{\theta}_{boxcar}$ (see above and Methods) and is plotted here (also shown by color of vertical bands to left of the heat maps in Figure 5e,f). Filled triangles indicate $p < 10^{-4}$, which occurred when θ_{boxcar} was less than all 10,000 values of $\hat{\theta}_{boxcar}$. The arbitrary threshold of p = 0.01 (dashed horizontal line) appeared to discriminate categorically distinct ranges of $p(\theta_{boxcar})$, justifying use of this threshold for display purposes (e.g., Figure 5e,f). However, the periods of putative stability were not consistently below this threshold. In particular, $p(\theta_{boxcar})$ for monkey K expected reward ((f), right panel) hovered around the significance threshold. In addition, the stability magnitude during these periods ((d), right panel, note smaller ordinate scale) was likewise smaller than for other variables.

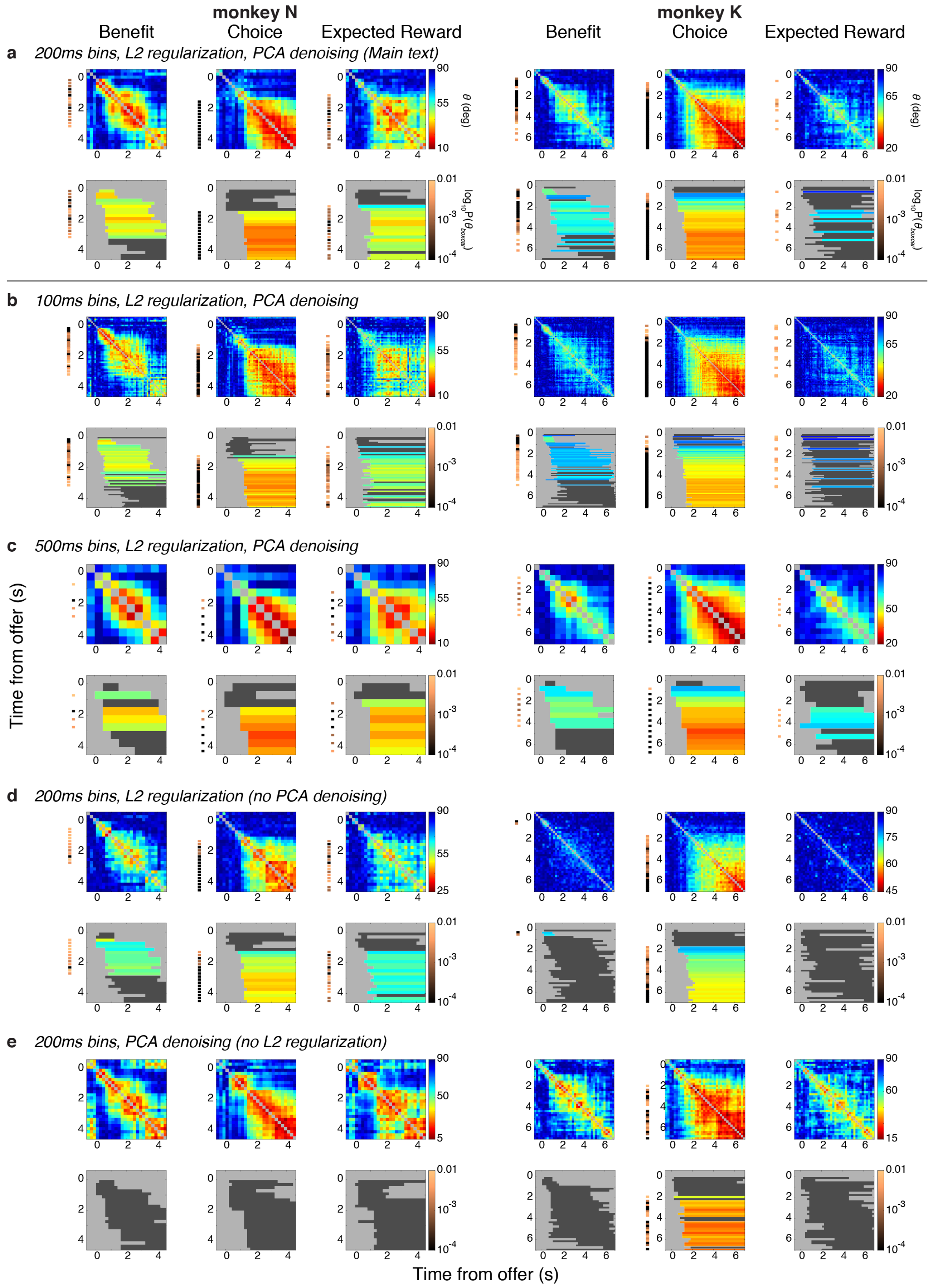

Supplementary Figure 22. Effect of varying model parameters on stability analysis.
(a-e) For monkeys N (left 3 columns) and K (right 3 columns), the stability of the dynamic low-dimensional representations (dRAs) of benefit, choice, and expected reward is shown (left, middle, and right columns,

respectively, for a given animal) for separate implementations of the dRA analysis. Each pair of rows corresponds to a separate analysis using distinct parameters (italicized headings). Conventions are as in Figure 5. In brief, for a given pair of rows, the top row (heatmaps) shows the angle θ in degrees between pairs of dRAs for the same variable as given by the pixel color (referencing right-hand color scale within row), while the pixel's grid position gives the times of the dRA pair relative to the onset of the offer. Smaller angles (warmer colors) correspond to greater similarity between representations. The bottom row shows the boxcar fits to the above heatmaps. Periods of significant similarity (i.e., $p(\theta_{boxcar}) < 0.01$) are colored, with bar color indicating boxcar height (i.e., extent of similarity during period spanned by the boxcar) and referencing right-hand color scale in above row. Non-significant boxcars ($p(\theta_{boxcar}) \geq 0.01$) are shaded dark gray. The thin vertical bands to the left of each panel in (c-f) show the $\log_{10}$ probability of observing θ_{boxcar} by chance for the corresponding time, with colors referencing the right-hand color scale in the lower of each pair of rows (no color is shown when $p(\theta_{boxcar}) \geq 0.01$).

(a) The analysis from the main text is recapitulated in (a). Alternative implementations are discussed below. In general, both the profile of angle similarity (heatmaps) and significance of boxcar fits did not depend qualitatively on the duration of dRA time bins (100, 200, or 500ms) or application of PCA-based denoising or regularization.

(b) Shortening the time bin from 200ms, as used in main text, to 100ms reduced the precision in estimating the dRA in any given time bin (indeed the dRA magnitudes, not shown, and angles were more variable across time), but did not change the fundamental stability conclusions: benefit was stable early (0 – 0.5 s), then changed to a new representation that was stable during the middle of the trial (0.5 s until about 1 s before the reward); choice was stable from about 1 s onward; and expected reward was stable from about 1 s onward for monkey N, but was equivocal for monkey K.

(c) Similarly, lengthening the time bin to 500 ms had little effect on the same fundamental conclusions with a single exception: no early period of stability (0 – 0.5 s) was found for benefit. This was easily explained by the fact that this entire period was subtended by a single 500 ms time bin, which by its singular nature could not be "stable" as there was no other time bin (within the period in question) with which to compare it!

(d) The effect of omitting PCA-based denoising is shown (regularization and 200 ms time bins were maintained). Compared to the main analysis (a), the absolute magnitude of similarity was reduced (note larger minimum angles in red-to-blue color bars), as expected given that the dRAs could now span a higher dimensional space, thus permitting higher extents of dissimilarity. However, this pressure toward dissimilarity was also present for the random surrogate data. Thus the similarity between the veridical dRAs was much greater than between the surrogate dRAs and, consequently, the estimated probability of observing the veridical dRAs by chance remained small (i.e., statistically significant), as observed by qualitatively similar boxcar plots (bottom row in (d)). Monkey K was an exception in that the absolute magnitudes of similarity (not shown) for benefit (during the middle period) and expected reward were so markedly reduced by the lack of denoising as to be indistinguishable from chance. Monkey K may have been particularly susceptible to the lack of denoising because of the markedly higher number of dimensions in its full dataset (342 units compared to monkey N's 68), making dissimilarity much more common. In addition, even in the main text analysis (a), the similarity during these periods was much lower for monkey K than N.

(e) The effect of omitting L2-regularization is shown (PCA-based denoising and 200 ms time bins were maintained). Recall that L2-regularization penalizes extreme values across the set of beta coefficients for the task-relevant variables for a given neuron within a given time bin. That is, the regularization assumes that a given neuron contributes at least partially to the representation of multiple variables (consistent with mixed selectivity). When the representations are robust within the population (i.e., high dRA magnitude), the impact of omitting regularization is minimal. However, when a significant degree of noise is present (i.e., firing rates are poorly explained by the task-relevant variables), unregularized dRAs will reflect these small, random changes in firing rate (i.e., overfitting) and vary accordingly over time. The stability heatmaps (e, top row) are consistent with these predictions: during the trial, when encoding of the task-relevant variables is strong, the similarity of dRAs is comparable to the main text analysis (a), albeit with slightly greater extremes of similarity (note smaller minimum angles in red-to-blue color bars). However, during periods of low or even absent encoding (e.g., prior to the offer), the unregularized dRAs exhibit some similarity. (Note: this similarity is not reflected in the boxcar plots because boxcars fits were limited to the period from offer onset to the end of the trial.) The putative similarity during this pre-offer time must be spurious (i.e., resulting from dRAs overfitted to random

variation) because the external information needed to drive benefit or expected reward signals did not exist prior to the offer. Applying regularization, as done in the main analysis (a), mitigates this overfitting.

Finally, the absence of regularization all but eliminated the statistical significance of the boxcars (e, lower row). We confirmed this was not due to a reduction in the magnitude of similarity (i.e., boxcar height) in the veridical data (not shown), which was nearly identical to that in the main analysis. Rather, the lack of significance was due to a massive increase in the height of the boxcars fit to the surrogate data. To provide an intuition for this phenomenon, consider computing the dRAs within a single surrogate dataset. Say, by chance, the average firing rate in a given time bin was highly correlated with benefit, but not so much with the other task-relevant variables. Because this correlation resulted from random variation, it did not generalize well when measured within subsets of trials (i.e., poor cross-validation) and thus would be suppressed by regularization. However, without regularization, the exclusive correlation with benefit and the lack of generalization were tolerated by the analysis, resulting in an unusually large beta coefficient for benefit and negligible coefficients for the other variables. In the adjacent time bins, the fact that temporal smoothness was preserved in the surrogate data caused the same random correlation with benefit to be maintained across several time bins, resulting in spuriously high similarity for that surrogate dataset. Of course, this spurious benefit signal would be absent in a different surrogate dataset, though a similar pattern of spurious similarity would be observed for a different variable. Across datasets, this process inflated the similarity within the surrogate data and thus increased our estimates of observing a given level of similarity by chance. Though some surrogate datasets lacked spurious levels of similarity for any variable, these datasets could not offset the spuriously high levels in other datasets because of a floor effect: similarity could not be less than zero. By applying regularization, we mitigated the tendency to overfit the dRAs and reduced the apparent similarity of dRAs explaining random variation in firing rate that did not generalize across trials. Ultimately, use of regularization in the main analysis more accurately estimated chance levels of similarity.

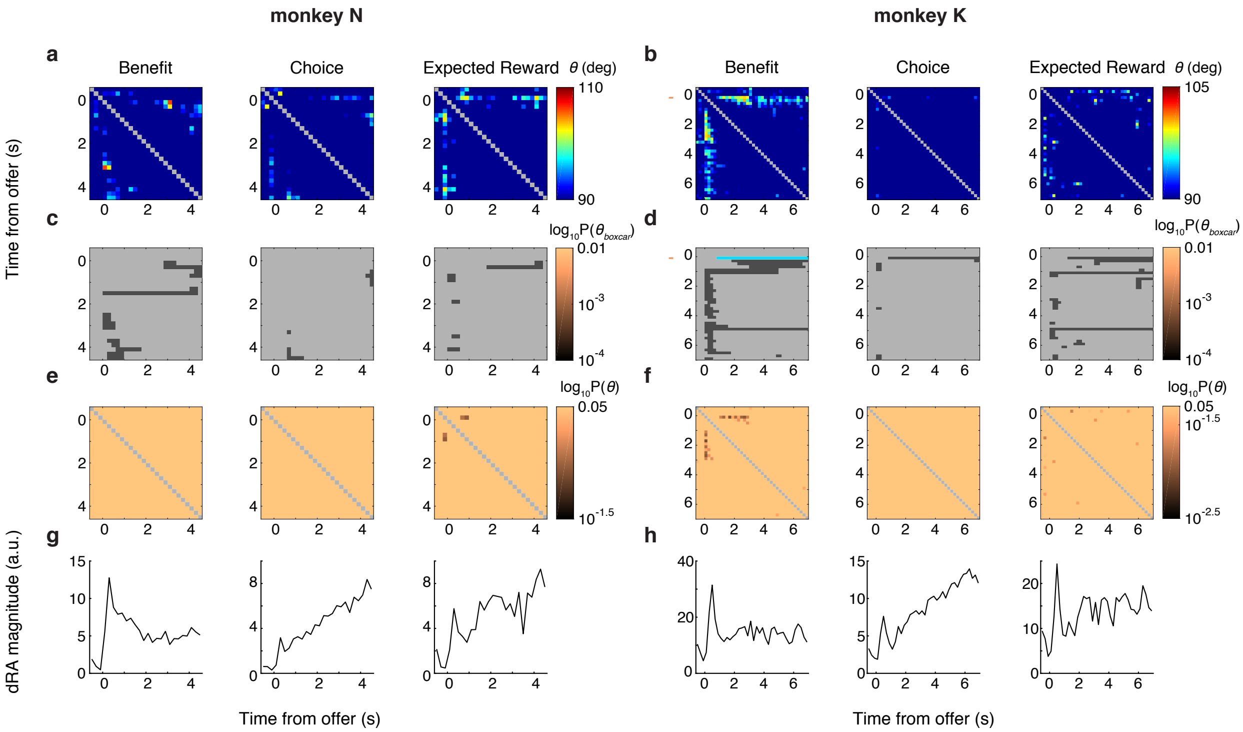

Supplementary Figure 23. Sign reversals in dynamic low-dimensional representations
The preceding similarity analyses examined the folded angle θ (Equation (15)), which measured the absolute similarity between dynamic low-dimensional representations (dRAs) by treating angles equidistant from 90° as equivalent. However, the folded angles did not capture reversals in the sign of representation, i.e., when the absolute magnitude of encoding remained consistent over the course of the trial, but the sign reversed (e.g., Figure 2d). To detect reversals, we computed the unfolded angle θ' (Equation (16)), which was limited to the range [90, 180°]. The present figure examines sign reversals for monkeys N (left 3 columns) and K (right 3 columns) based on θ' between the dynamic representations (dRAs) for benefit, choice, and expected reward (left, middle, and right columns, respectively, for a given animal).

(a,b) Angle θ' in degrees between a pair of dRAs of the same variable is given by the color of each pixel (referencing right-hand color scale in (a,b)), and the times of the dRA pair (relative to the onset of the offer period) are given by the pixel's row and column positions. Larger angles (warmer colors) correspond to greater similarity between representations but with opposite sign of representation. Values of $\theta' < 90°$ were excluded from statistical analysis and were coded as 90° for display purposes only. The diagonal compares identical dRAs and is colored gray and was excluded from analysis. **(c,d)** Boxcar function with baseline of 90° was fit to each row of angles in (a,b), and the period of non-zero boxcar height is indicated by a colored or dark gray horizontal bar in the corresponding row of (c,d). Periods of significant similarity (i.e., $p(\theta'_{\text{boxcar}}) < 0.01$) are colored, where bar color represents the boxcar height θ'_{boxcar}, i.e., magnitude of similarity, measured in degrees and referencing the right-hand color scale in (a,b). When $p(\theta'_{\text{boxcar}}) < 0.01$, the value of p is indicated by the color of the vertical band to the left of the corresponding row in (a-d) and references the right-hand color scale in (c,d). (In effect, only a single row for a single variable was significant; (b,d), right panel). Boxcars for which $p(\theta'_{\text{boxcar}}) \geq 0.01$ are shaded dark gray in (c,d). Periods where $\theta_{\text{boxcar}} \leq 90°$ are shown in light gray. **(e,f)** The $\log_{10}$ probability of observing by chance the pairwise similarity θ' (a,b)—not the period of contiguous similarity, θ'_{boxcar}—is given by the color of the corresponding pixel and references the right-hand color scale in (e,f). The diagonal compares identical dRAs and is colored gray. **(g,h)** Recapitulated from Figure 5, the vector magnitude of each dRA is shown as a function of time from the onset of the offer period and provides a reference as to the magnitude of population encoding available for representation at a given time.

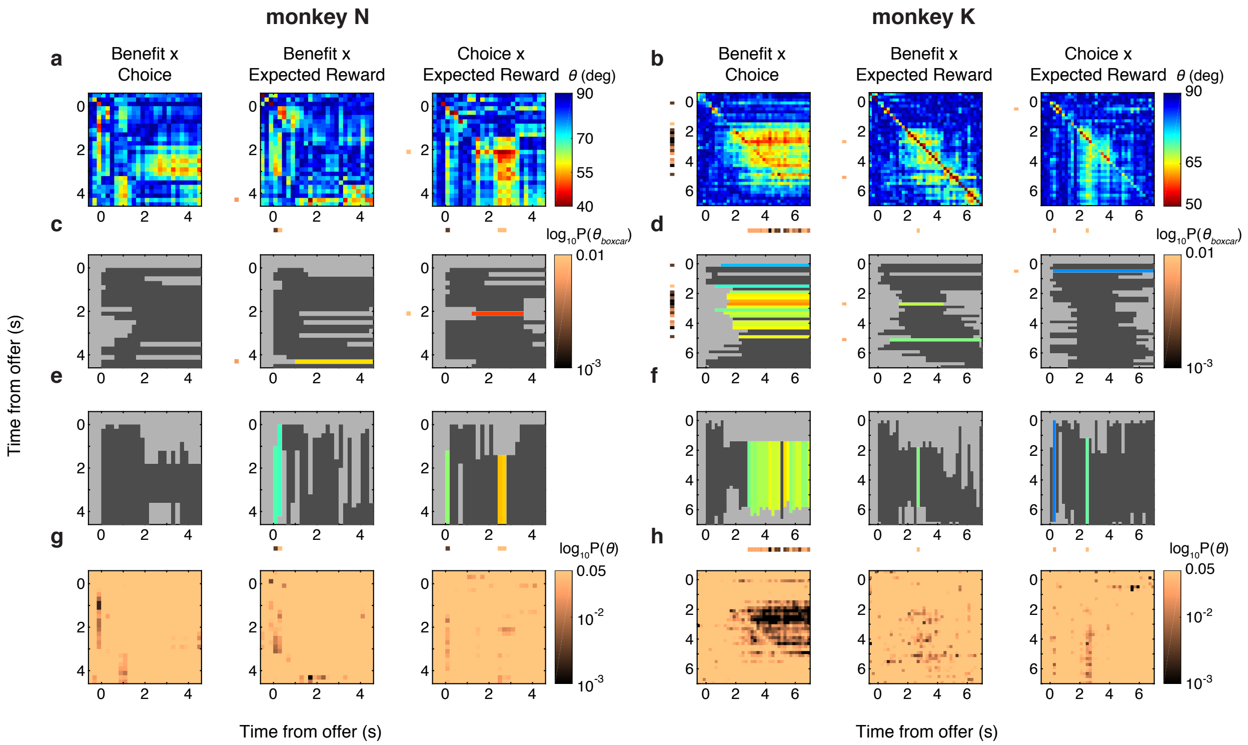

Supplementary Figure 24. Similarity between representations of different variables.
The preceding similarity analyses compared dynamic low-dimensional representations (dRAs) of the same task-relevant variable. Here we examined the similarity, or "cross-talk", between dRAs of different variables for monkeys N (left 3 columns) and K (right 3 columns). For a given animal, the dRAs between variables A and B (A x B) were compared: benefit x choice (left column), benefit x expected reward (middle column), and choice x expected reward (right column). **(a,b)** Angle θ_{ij} in degrees between pair of dRAs for variable A at time *i* (ordinate) and variable B at time *j* (abscissa) is given by the color of pixel *ij* (referencing right-hand color scale in (a,b)), and the times of the respective dRAs relative to offer onset are given by the pixel's row and column position. Smaller angles (warmer colors) correspond to greater similarity between representations. The colored bands to the left of or below each panel are described below. **(c-f)** Unlike comparing dRAs of the same variable (e.g., Figure 5), the present matrix of angles θ was not symmetrical. Thus, a boxcar function was fit separately to each row or column of angles in (a,b) and the period of non-zero boxcar height is indicated by the horizontal or vertical bar in the corresponding row or column of (c,d) or (e,f), respectively. Periods of significant similarity ($p(\theta_{boxcar}) < 0.01$) are colored, where bar color represents the boxcar height θ_{boxcar}, i.e., magnitude of similarity, measured in degrees and referencing the right-hand color scale in (a,b). The $\log_{10}$ probability p of observing θ_{boxcar} is indicated by the color of the vertical or horizontal band to the left of or below the corresponding row or column in (a-f), respectively, and the colors reference the right-hand color scale in (c,d). Boxcars for which $p(\theta_{boxcar}) \geq 0.01$ are shaded dark gray and the corresponding entries within the colored bands are omitted. Periods where $\theta_{boxcar} = 0$ are shown in light gray. **(g,h)** The $\log_{10}$ probability of observing by chance the pairwise angle θ_{ij} in (a,b) is given by the color of the corresponding pixel and references the right-hand color scale in (g,h).

We observed that the representations of different variables overlapped during periods of the trial. For instance, the mid-trial dRAs for benefit were similar to the mid-to-late trial dRAs for choice (a,b, left panels), though the similarity was only statistically significant for monkey K. This overlap suggested a means to compute expected reward, as a readout tuned to this mid-trial shared representation would intrinsically integrate offer and choice information. However, though compelling conceptually, the timing of the representations in the present study was inconsistent with this mechanism: the EXPECTED REWARD sRA detected significant RSV prior to when the benefit and choice dRAs overlapped (Figure 4c-f). Future studies with simultaneously recorded units may examine these interactions of representations over time to understand how the network computes a given variable.

Previous-trial low-dimensional representations

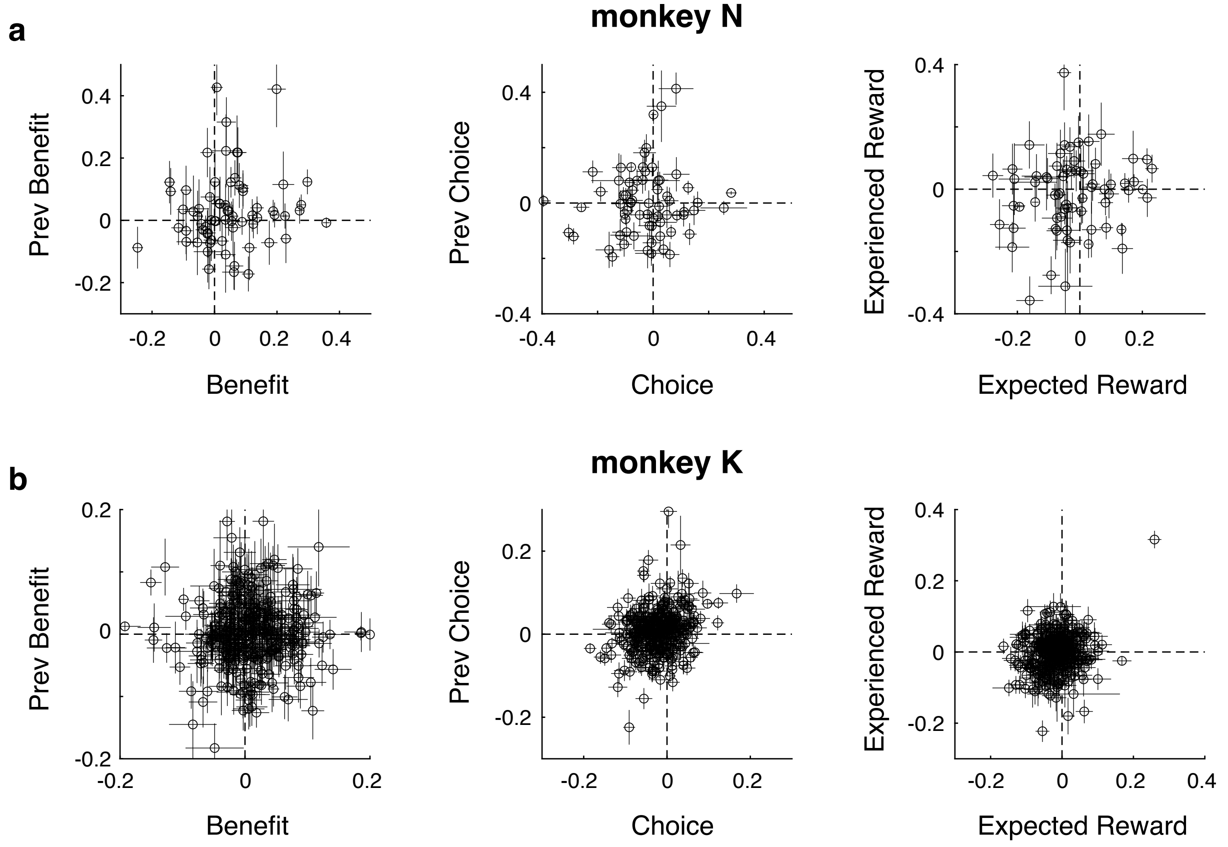

Supplementary Figure 25. Relationship between present- and previous-trial representations. **(a,b)** Regression coefficients for the present- (abscissa) and previous-trial (ordinate) representations (i.e., sRAs) of the same variable are shown for individual units (open circles; point estimates derived from all trials) with associated standard deviations (horizontal and vertical error bars; derived from N=700 resampled datasets per unit, see Methods) for monkeys N (a) and K (b). The horizontal and vertical meridians (dashed lines) indicate the location of hypothetical units representing the given variable for only the present or previous trial (but not both). Most units did not intersect these meridians, consistent with mixed representations of present- and previous-trial variables at the level of individual units. See Supplementary Table 3 for correlation coefficients and angles between present- and previous-trial sRAs. For current figure, sRAs were derived without orthogonalizing regression axes.

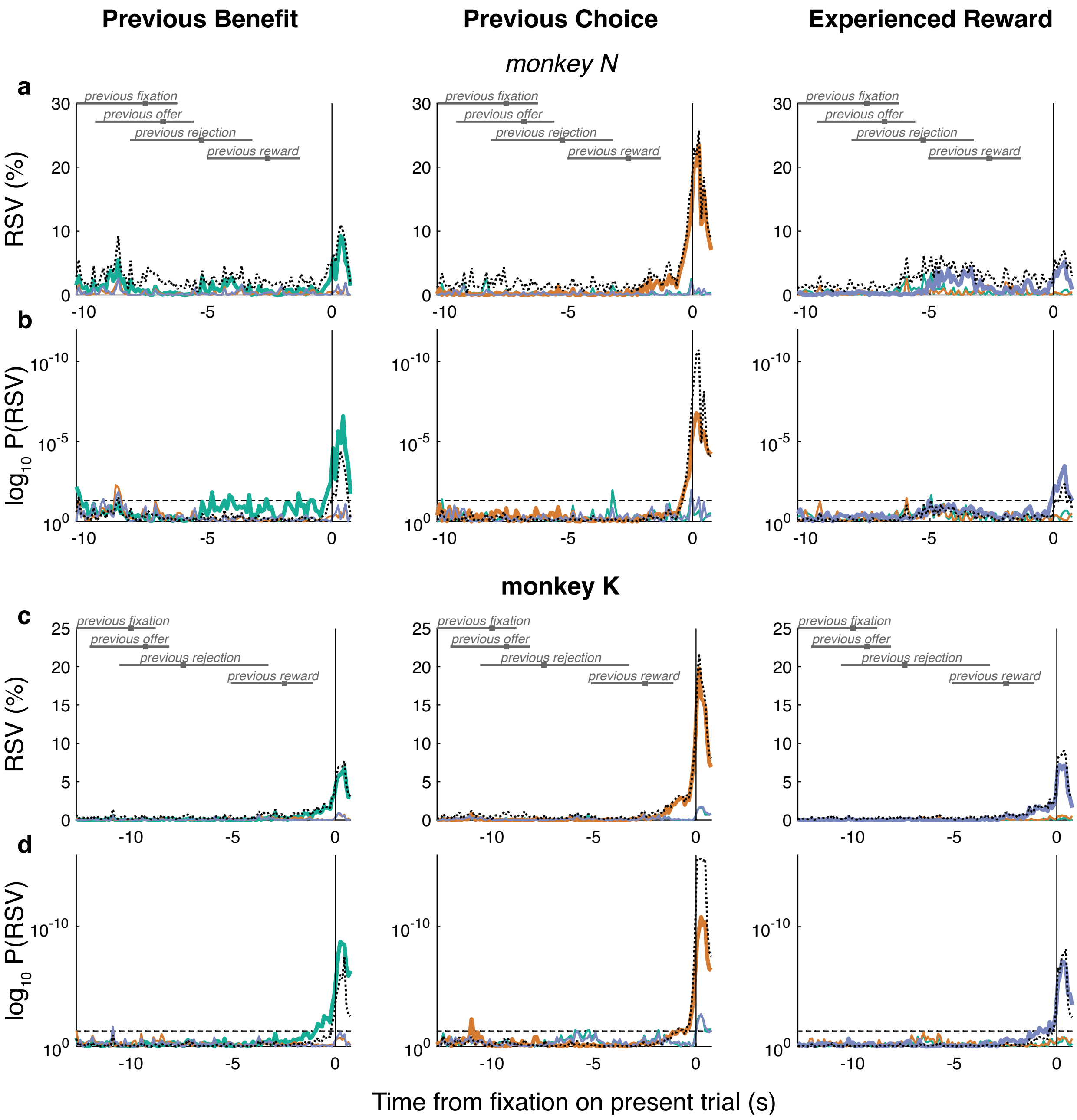

Supplementary Figure 26. Specificity of previous-trial low-dimensional representations.
(a-d) For detailed explanation of plotting conventions, refer to the analogous Supplementary Figure 14. Both figures demonstrate the specificity of a set of low-dimensional representations (i.e., sRAs) to their targeted variables of interest. Whereas the prior figure concerned representations of present-trial variables during the present offer and work periods, the current figure concerns representations of variables from the previous trial—previous benefit (green), previous choice (orange), and experienced reward (blue)—during a period aligned to the present fixation and extending retrospectively to the previous fixation. The on-target relevant signal variance (RSV; thick curves), off-target partial RSV (thin curves), and overall variance explained (dotted black curves) for the previous-trial sRAs (columns, as labeled) are shown in (a,c) with associated $\log_{10}$ probability shown in (b,d) for monkeys N (a,b) and K (c,d). Horizontal gray bars and solid gray squares indicate the 2.5-to-97.5 percentile range and median, respectively, of previous trial events, as labeled.

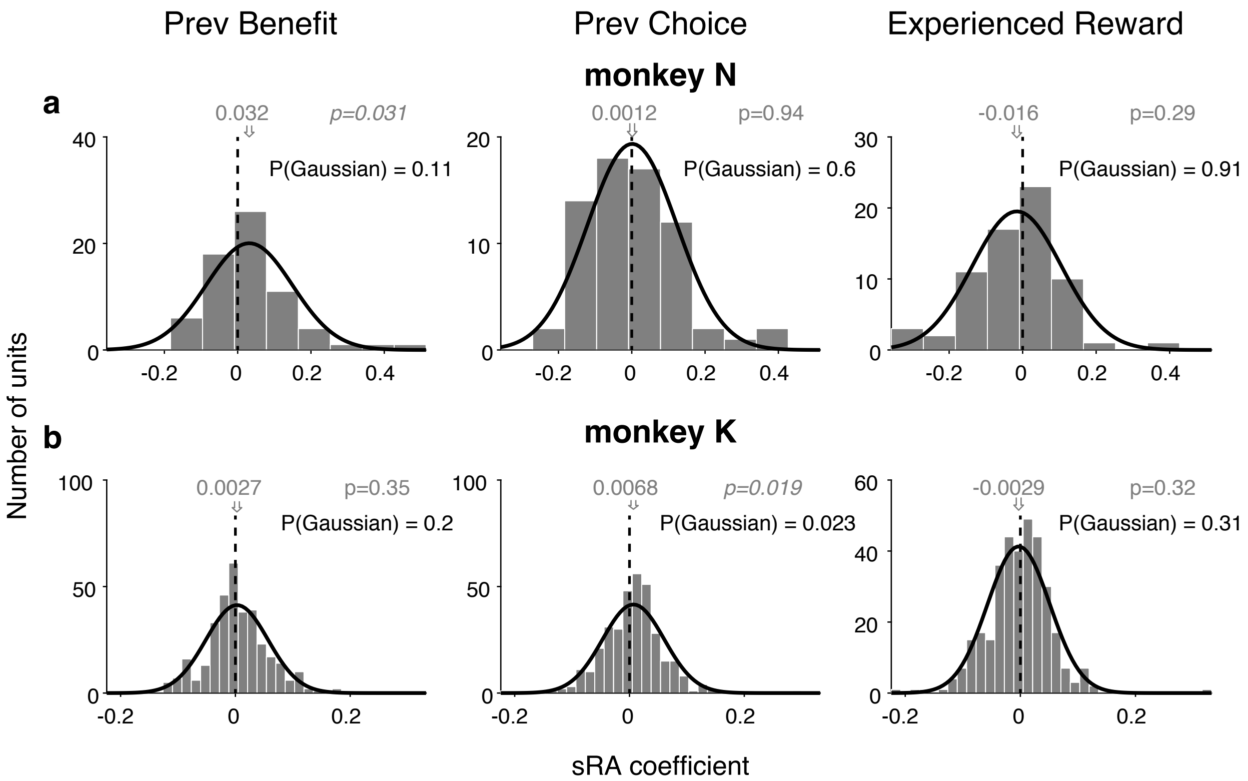

Supplementary Figure 27. Contribution of individual units to previous-trial representations. **(a,b)** Histogram of regression coefficients specifying each unit's contribution to the static low-dimensional representations (sRAs) of the previous-trial variables: PREVIOUS BENEFIT (left panels), PREVIOUS CHOICE (middle panels), and EXPERIENCED REWARD (right panels) for monkeys N (a) and K (b). Distribution mean (gray arrow and text) and p-value (gray text; via two-sided *t*-test) of null hypothesis that mean = 0 (vertical dashed line) are shown. Gaussian functions were fit to each distribution (black curves). Probability (black text) of falsely rejecting the null hypothesis that observed distribution was Gaussian was computed via two-tailed Kolmogorov–Smirnov test. No distribution except one differed significantly from Gaussian. For present figure, sRAs were derived without orthogonalizing regression axes.

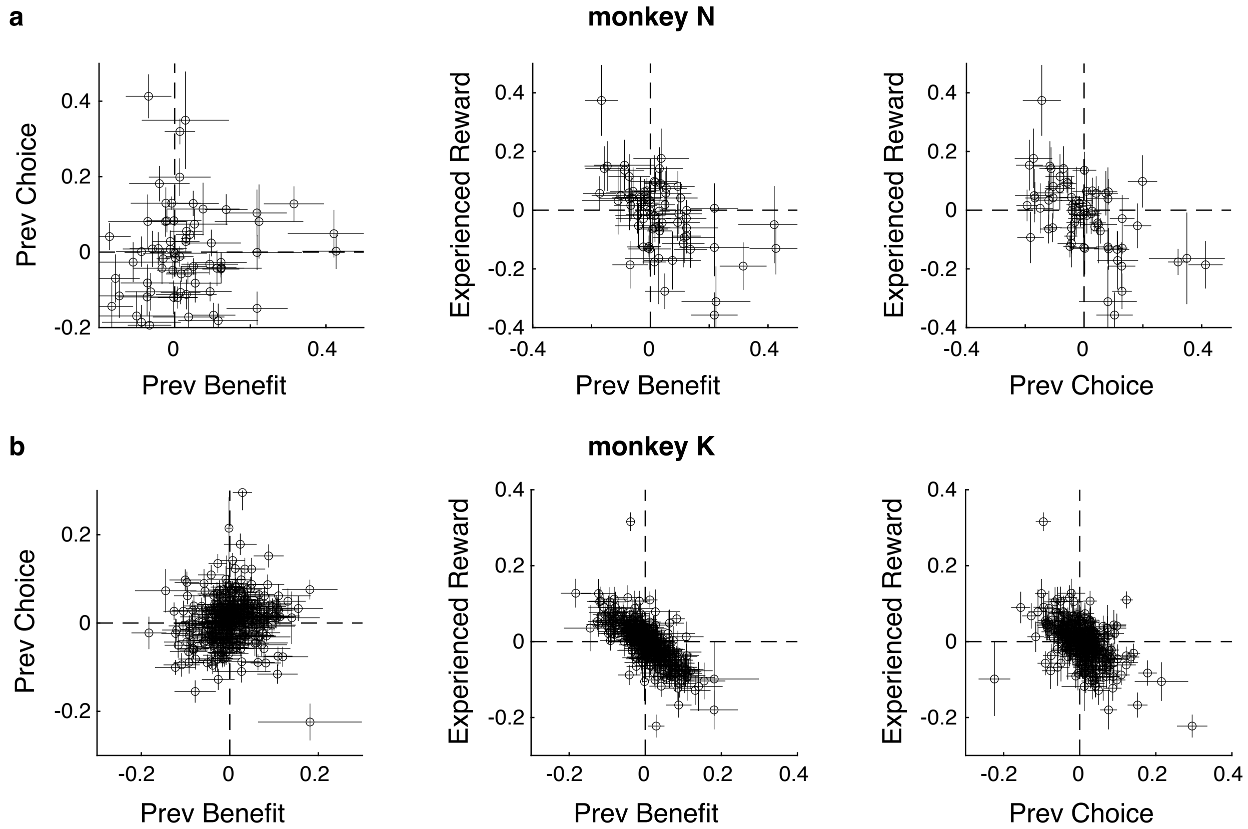

Supplementary Figure 28. Relationship between previous-trial representations.
(a,b) As for the present-trial representations in Figure 3a,c, the pairwise relationship between regression coefficients for the previous-trial representations (i.e., sRAs; abscissa and ordinate labels) are shown for individual units (open circles; point estimates derived from all trials) with associated standard deviations (horizontal and vertical error bars; derived from N=700 resampled datasets per unit, see Methods) for monkeys N (a) and K (b). The horizontal and vertical meridians (dashed lines) indicate the location of hypothetical units representing a solitary variable. Most units did not intersect these meridians, consistent with mixed representations at the level of individual units. See Supplementary Table 4 for correlation coefficients and angles between previous-trial sRAs. For present figure, sRAs were derived without orthogonalizing regression axes.

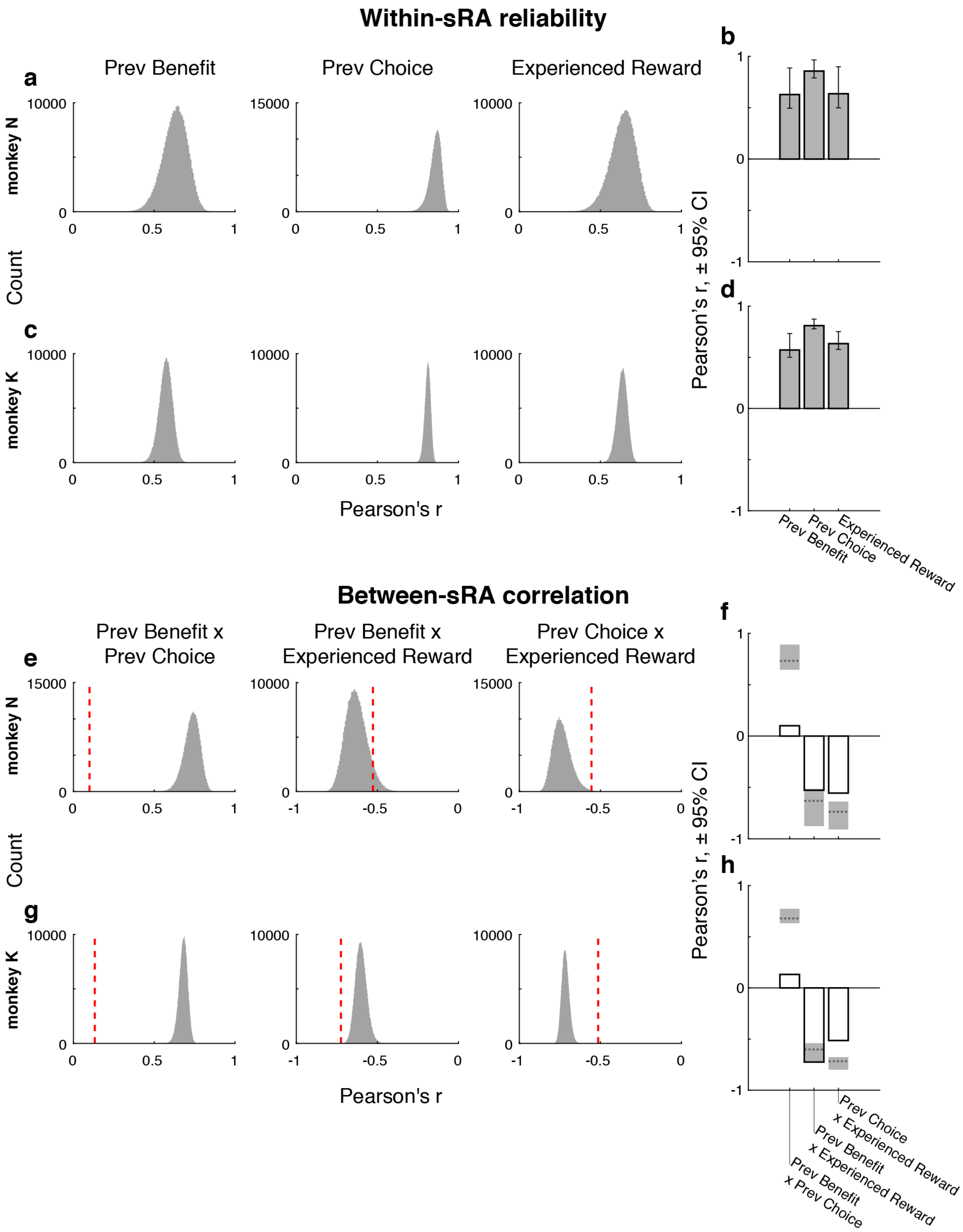

Supplementary Figure 29. Reliability and separability of previous-trial representations.
Figure refers to previous-trial representations (i.e., sRAs). For detailed description of analogous procedures on present-trial representations, see Supplementary Figure 7 for (a,c,e,g) and Figure 3b,d for (b,d,f,h). **(a,c)** Histograms of within-variable correlation coefficient $\tilde{r}_{ii}$ across resampled datasets are

shown for monkeys N (a) and K (c), revealing high reliability of the sRAs. **(b,d)** The mean and 95% CI of the distributions of $\tilde{r}_{ii}$ (as shown in (a,c)) are summarized by bar height and error bars, respectively, for monkeys N (b) and K (d). **(e,g)** Histograms of the null model of between-variable correlation coefficients $\tilde{r}_{ij}$ are shown for representations of each pair of variables *i* and *j* and compared to the observed correlation r_{ij} (vertical dashed red line) for monkeys N (e) and K (g). Null model refers to the hypothetical correlation between two perfectly correlated representations corrupted by independent noise (i.e., low reliability of the coefficients), as measured across resampled datasets. **(f,h)** The observed and hypothetical between-variable correlations are summarized for monkeys N (f) and K (h). Bar height corresponds to observed correlation r_{ij} (i.e., red vertical dashed line in (e,g)). Gray dashed horizontal line and shading indicate the mean and 95% confidence interval (CI), respectively, of the hypothetical correlation $\tilde{r}_{ij}$ (i.e., histograms in (e,g)). In all but one case, observed correlations were closer to zero (i.e., less correlated) than 95% of the null hypothetical correlations, defining the pairs of representations as separable. The resampled data included 700 resampled datasets generating N=244,650 pairwise correlations. See Supplementary Table 4 for summary statistics. For present figure, sRAs were derived without orthogonalizing regression axes.

Computing low-dimensional representations exclusively in single units

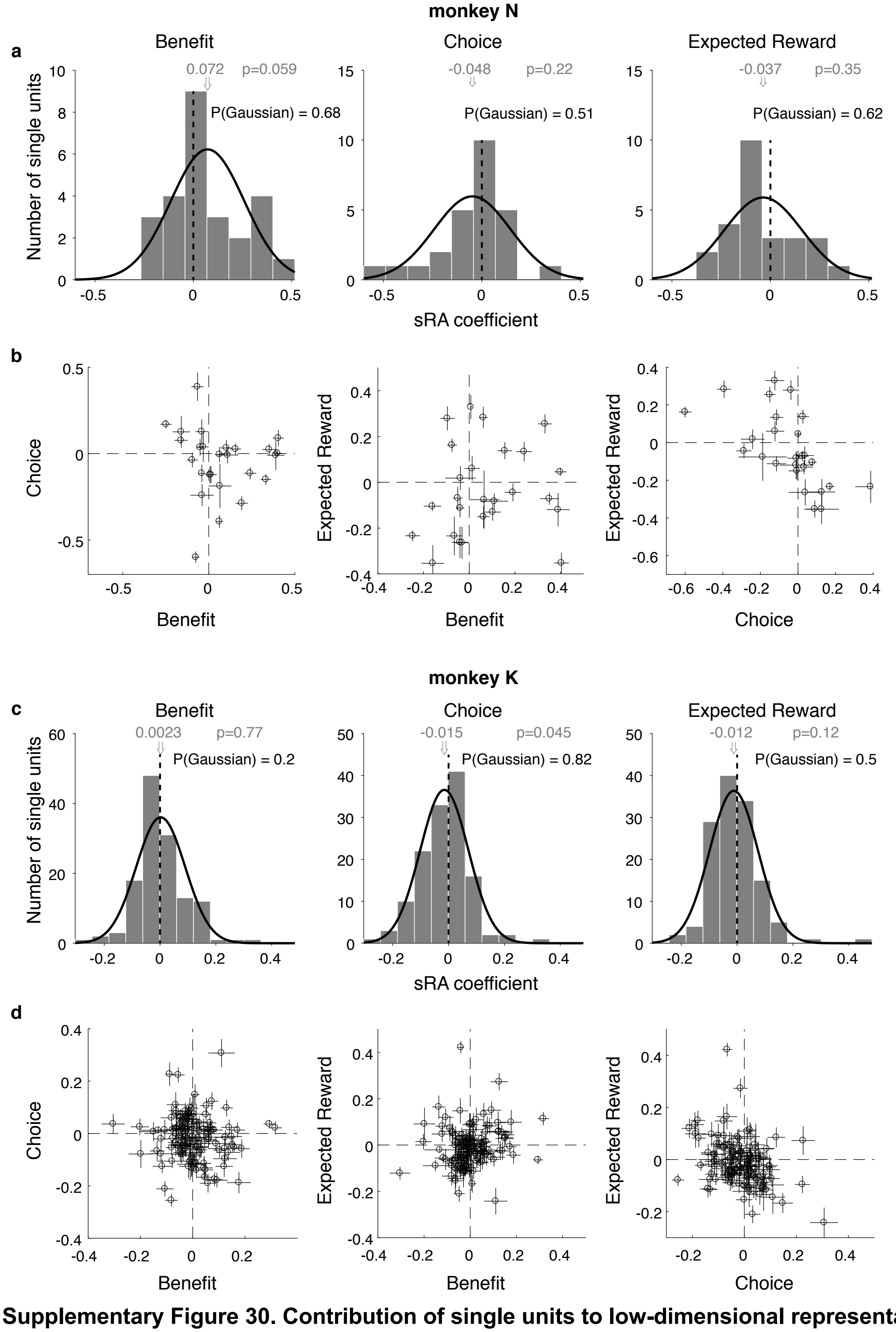

Supplementary Figure 30. Contribution of single units to low-dimensional representations. We separately discovered the low-dimensional representations (sRAs) in a subset of data limited to single units. **(a,c)** As in Supplementary Figure 5a,b, histograms of regression coefficients that determined the

contribution each single unit to the sRAs of BENEFIT (left panels), CHOICE (middle panels), and EXPECTED REWARD (right panels) are shown for monkeys N (a) and K (c). Distribution mean (gray arrow and text) and p-value (gray text; via two-sided *t*-test) of null hypothesis that mean = 0 (black vertical line) are shown. Probability (black text) of falsely rejecting null hypothesis that observed distribution was Gaussian was computed via two-tailed Kolmogorov–Smirnov test. No distribution differed significantly from Gaussian. **(b,d)** As in Figure 3a,c, the pairwise relationship between regression coefficients for task-relevant variables (abscissa and ordinate labels) are shown for single units (open circles; point estimates derived from all trials) with associated standard deviations (horizontal and vertical error bars; derived from N=700 resampled datasets per unit, see Methods) for monkeys N (b) and K (d). The horizontal and vertical meridians (dashed lines) indicate the location of hypothetical units representing a solitary variable. Most units did not intersect these meridians, consistent with mixed representations at the level of single units. See Supplementary Table 2 for correlation coefficients and angles between single-unit sRAs. For present figure, all regression coefficients were found without orthogonalizing the sRAs.

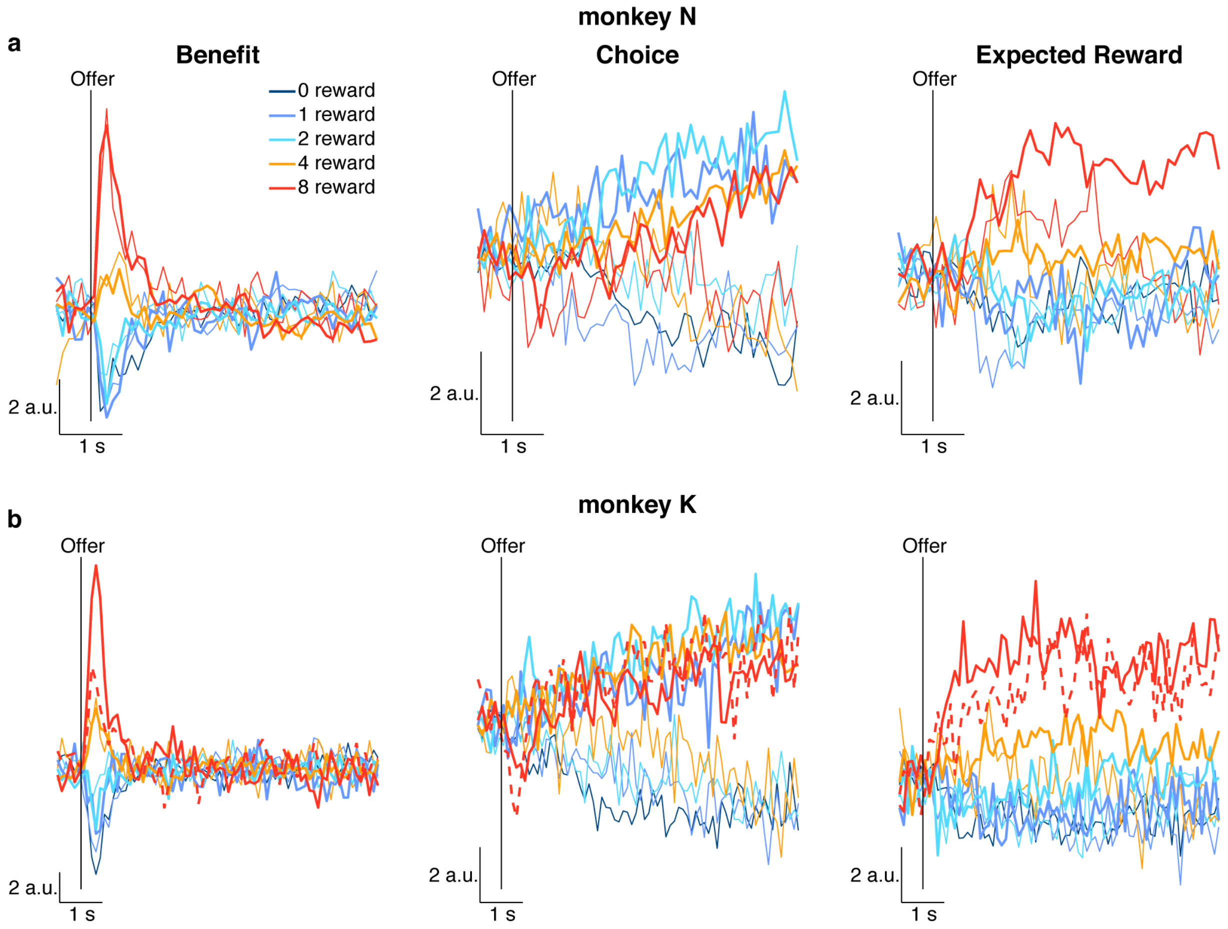

Supplementary Figure 31. Activity of single unit-based low-dimensional representations. We separately discovered the low-dimensional representations (sRAs) in a subset of data limited to single units and projected the population response for each combination of offer size (colors, see legend) and choice (accept or reject choices in thick or thin curves, respectively) onto the sRAs of BENEFIT (left panels), CHOICE (middle panels), and EXPECTED REWARD (left panels) as a function of time from the onset of the offer period (vertical black lines) for monkeys N (a) and K (b). The sensitivity of the sRAs for the task-relevant variables did not differ qualitatively between single units (shown here) and the entire population (Figure 4a,b). See Supplementary Figure 32 for additional metrics.

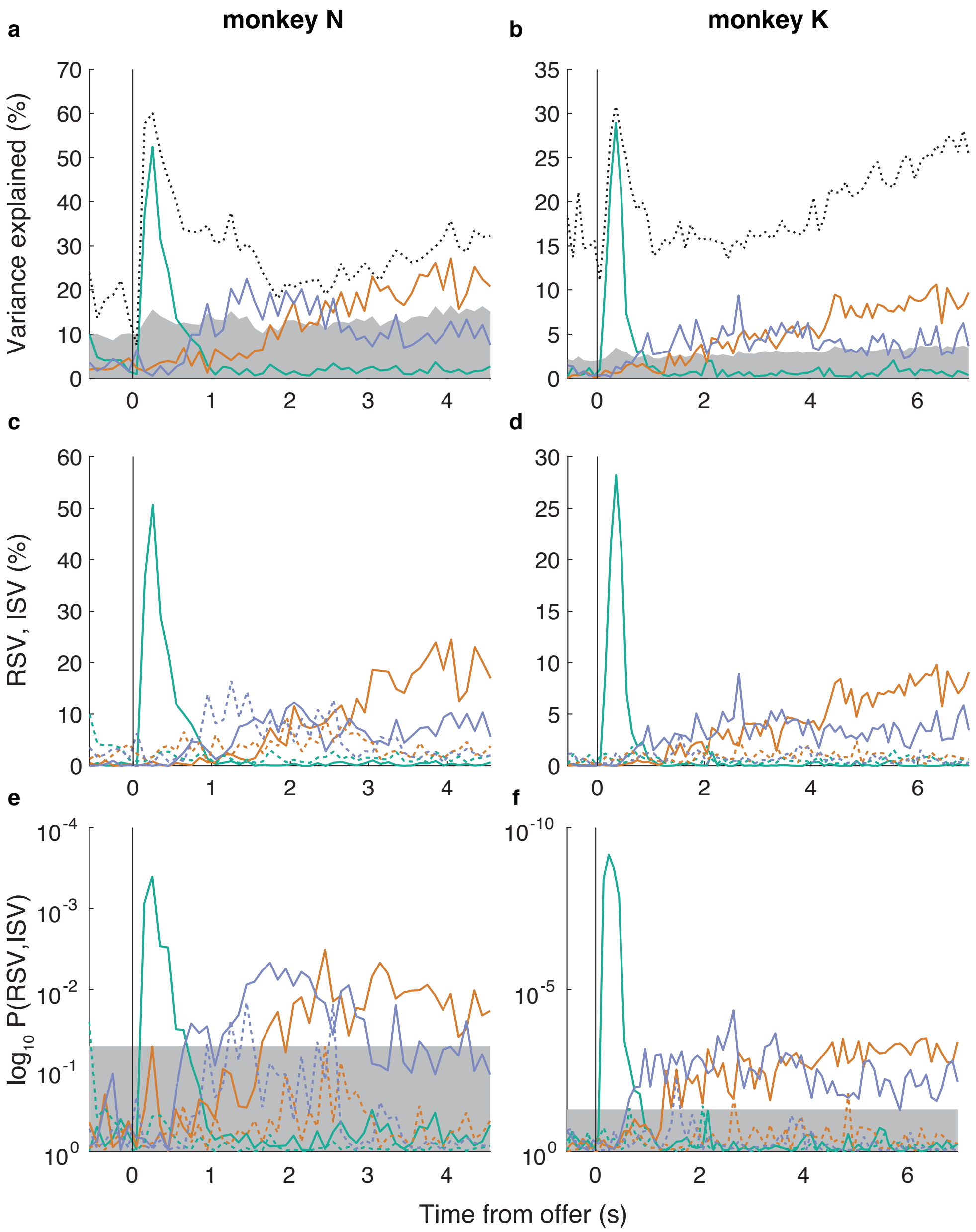

Supplementary Figure 32. Variance explained by single unit-based low-dimensional representations.

We separately discovered the low-dimensional representations (sRAs) in a subset of data limited to single units for monkeys N (a,c,e) and K (b,d,f). **(a,b)** As for the full population in Supplementary Figure 13a, the variance explained by these single-unit based sRAs of BENEFIT (green curve), CHOICE (orange curve), and EXPECTED REWARD (blue curve) is plotted as a function of time from the onset of the offer period (black vertical line). The area of gray shading represents the variance explained by 95% of random vectors reflecting the dimensionality of the data (redefined here for the population of single units). To estimate the upper-bound for variance explained, we performed principal components analysis independently at each time bin and plotted the variance explained by the top component (black dotted curve), which represented the maximal variance explained by any single, time-varying dimension. **(c,d)** As in Figure 4c,d, we plotted the relevant and irrelevant signal variance (RSV and ISV, solid and dashed lines, respectively) explained by the sRAs (colors as above) with respect to the targeted variable of interest (e.g., RSV explained by the BENEFIT sRA with respect to the benefit variable). **(e,f)** As in Figure 4e,f, we plotted the $\log_{10}$ probability (one-sided, uncorrected) of RSV and ISV, which was derived empirically in comparison to the null distribution of random vectors (see Methods). Area of gray shading represents $p > 0.05$.

Supplementary Tables

Supplementary Table 1. Significant encoding of task-relevant variables in individual units.

	Monkey N			Monkey K		
**Variable A**	Benefit	Choice	Expected Reward	Benefit	Choice	Expected Reward
1. Included	67*	67*	67*	340*	340*	340*
2. Significant	46 (69%)	33 (49%)	41 (61%)	138 (41%)	147 (43%)	107 (31%)
Single Units						
3. Included	25*	25*	25*	129*	129*	129*
4. Significant	17 (68%)	11 (44%)	19 (76%)	47 (36%)	48 (37%)	39 (30%)
**Overlap with Variable B**	Choice	Expected Reward	Benefit	Choice	Expected Reward	Benefit
5. Both sig.<br>obs. / *exp.*	25 (37%)<br>*22.7 (34%)*	20 (30%)<br>*20.2 (30%)*	32 (48%)<br>*28.1 (42%)*	66 (19%)<br>*59.7 (18%)*	59 (17%)<br>*46.3 (14%)*	56 (16%)<br>*43.4 (13%)*
6. Both non-sig.	13 (19%)<br>10.7 (16%)	13 (19%)<br>13.2 (20%)	12 (18%)<br>8.15 (12%)	121 (36%)<br>115 (34%)	145 (43%)<br>132 (39%)	151 (44%)<br>138 (41%)
7. A sig. only	21 (31%)<br>*23.3 (35%)*	13 (19%)<br>*12.8 (19%)*	9 (13%)<br>*12.9 (19%)*	72 (21%)<br>*78.3 (23%)*	88 (26%)<br>*101 (30%)*	51 (15%)<br>*63.6 (19%)*
8. B sig. only	8 (12%)<br>*10.3 (15%)*	21 (31%)<br>*20.8 (31%)*	14 (21%)<br>*17.9 (27%)*	81 (24%)<br>*87.3 (26%)*	48 (14%)<br>*60.7 (18%)*	82 (24%)<br>*94.6 (28%)*
9. $p(\chi^2,\text{df})$	*0.22* (1.5, 1)	*0.92* (0.01, 1)	*0.037* (4.3, 1)	*0.16* (2.0, 1)	*0.0027* (9.0, 1)	*0.0028* (8.9, 1)

Number (percentage) of total included units or units with significant regression coefficients ($p < 0.05$ based on the trial-level resampling procedure, see Methods) are listed in the first or second rows for all units, and in rows 3 or 4 for coefficients discovered separately in a subset of data limited to single units, respectively. In rows 5 to 8, the coincidence of significant coefficients observed for task-relevant variables A (top header) and B (second header) are reported as the number (percentage) of units for which both, neither, only variable A only, or only variable B coefficient(s) were significant, respectively. Below each observed (obs.) value, the expected (exp.) number (percentage) of units is reported in *italics* assuming independence between the neural representations. For instance, if X and Y were the percentage of significant units for variables A and B, respectively, then the expected percentage of units significant for both A and B was given by X*Y. We compared the observed and expected values via the χ^2 statistic: $\chi^2 = (obs - exp)^2/exp$. The χ^2 statistic and associated p-value (taken as the integral of the χ^2-distribution with df = 1 from χ^2 to Inf) are reported in row 9.

For all pairs of variables, we observed equal or more units that were jointly significant for both variables (or neither variable) than expected given independence between variables (compare observed and expected values in data rows 5 and 6). Likewise, we observed fewer than expected units that were significant for a single variable (rows 7 and 8). We concluded that significant encoding of the task-relevant variables was at least distributed independently across the variables—and in some cases encoding of multiple variables was enriched beyond that expected by independence (i.e., $p(\chi^2) < 0.05$)—thereby leading to mixed selectivity for multiple variables at the level of individual units.

*One or two (monkey N or K, respectively) units were excluded from the present analysis because, for at least one variable, the distribution of coefficients across resampled datasets was not well-fit by a normal distribution, thus precluding any conclusions about their significance. Note: these distributions are distinct from the distributions of coefficients *across units*, which were well-fit by a normal distribution (Supplementary Figure 5).

For present table, all regression coefficients were found without orthogonalizing the sRAs.

Supplementary Table 2. Reliability of and correlation between low-dimensional representations.

	Monkey N			Monkey K		
**Within-variable**	BENEFIT	CHOICE	EXPECTED REWARD	BENEFIT	CHOICE	EXPECTED REWARD
$\tilde{r}_{ii}$	0.96	0.90	0.92	0.85	0.81	0.72
$p(\tilde{r}_{ii} = 0)$	$< 10^{-16}$*	$< 10^{-16}$*	$< 10^{-16}$*	$< 10^{-16}$*	$< 10^{-16}$*	$< 10^{-16}$*
**Between-variable**	BENEFIT x CHOICE	BENEFIT x EXPECTED REWARD	CHOICE x EXPECTED REWARD	BENEFIT x CHOICE	BENEFIT x EXPECTED REWARD	CHOICE x EXPECTED REWARD
r_{ij}	-0.15	0.27	-0.49	-0.20	0.17	-0.28
$p(r_{ij} = 0)$	0.23	0.027	$2.4 * 10^{-5}$	$2.5 * 10^{-4}$	$2.2 * 10^{-3}$	$1.6 * 10^{-7}$
$mean(\tilde{r}_{ij})$	0.93	0.94	0.91	0.83	0.78	0.76
$p(\tilde{r}_{ij} = \|r_{ij}\|, \tilde{r}_{ij} > \|r_{ij}\|)$	$< 10^{-16}$*	$< 10^{-16}$*	$< 10^{-16}$*	$< 10^{-16}$*	$< 10^{-16}$*	$< 10^{-16}$*
θ_{ij}	77°	80°	65°	76°	84°	84°
$p(\hat{\theta} = \theta_{ij}, \hat{\theta} > \theta_{ij})$	0.3	0.4	0.03	0.03	0.4	0.4
$p(\hat{\theta} = \theta_{ij}, \hat{\theta} < \theta_{ij})$	0.8	0.6	0.97	0.97	0.6	0.6
Single units						
r_{ij}	-0.086	0.13	-0.62	0.062	0.16	-0.35
$p(r_{ij} = 0)$	0.68	0.52	$7.1 * 10^{-4}$	0.48	0.067	$4.4 * 10^{-5}$
θ_{ij}	80°	87°	57°	86°	81°	72°
$p(\hat{\theta} = \theta_{ij}, \hat{\theta} > \theta_{ij})$	0.5	0.8	0.02	0.6	0.2	0.006
$p(\hat{\theta} = \theta_{ij}, \hat{\theta} < \theta_{ij})$	0.5	0.2	0.98	0.4	0.8	0.99

Pearson correlation coefficients and associated angles pertaining to within-variable reliability and between-variable correlations of non-orthogonalized low-dimensional representations (sRAs).

$\tilde{r}_{ii}$ refers to the correlation between pairs of representations of task-relevant variable $i \in$ {BENEFIT, CHOICE, EXPECTED REWARD} across N=700 resampled datasets and was used as a measure of within-variable reliability (Figure 3; Supplementary Figure 7; Methods). r_{ij} refers to the correlation observed between representations of variables *i* and *j*, and associated $p(r_{ij} = 0)$ is the conventional statistic against null hypothesis of r = 0 (Student's *t* test). $\tilde{r}_{ij}$ refers to the null distribution of correlation between representations of variables *i* and *j* assuming the variables were perfectly correlated (or anti-correlated) but subject to independent noise, as estimated from $\tilde{r}_{ii}$.

θ_{ij} refers to the folded angle (range [0, 90°]) between representations (i.e., dimensions) of variables *i* and *j*. $\hat{\theta}$ refers to the null distribution of angles measured empirically between random dimensions (Methods) and to which angles θ_{ij} were compared.

For 2-tailed tests, $p(H_0)$ refers to the probability of falsely rejecting the null hypothesis H_0 in favor of the unsigned alternative hypothesis. For 1-tailed tests, $p(H_0, H_1)$ refers to the probability of falsely rejecting the null hypothesis H_0 in favor of the alternative hypothesis H_1. By convention, H: $X = \mu$ is the hypothesis that the values X arose from a distribution with mean μ.

When $p(\tilde{r}_{ij} = |r_{ij}|, \tilde{r}_{ij} > |r_{ij}|)$ was small, the representations of *i* and *j* were less correlated than expected given their respective reliability, i.e., they were *separable*. All pairs of sRAs were highly separable.

When $p(\hat{\theta} = \theta_{ij}, \hat{\theta} > \theta_{ij})$ or $p(\hat{\theta} = \theta_{ij}, \hat{\theta} < \theta_{ij})$ was small, the angle between representations *i* and *j* was smaller or larger (i.e., the representations were more similar or different), respectively, than expected given the dimensionality of the data (i.e., intrinsic correlations between neurons; see Methods). The null hypothesis $\hat{\theta} = \theta_{ij}$ was tested separately against each 1-tailed alternative hypothesis because a) the implications of each alternative were distinct (i.e., the observed representations were more similar, less similar, or no different than expected by chance) and b) given that the distribution $\hat{\theta}$ was not symmetrical, the probability of each alternative could not be inferred from the two-tailed probability $p(\hat{\theta} = \theta_{ij})$.

Most pairs of sRAs were not more similar than chance, and no pairs were more different than chance. That is, in general, the extent of separation between sRAs was consistent with independent encoding given the limits on independence placed by the dimensionality of the data.

Values below the dashed line were computed from sRAs discovered independently in a subset of data limited to single units, which showed a similar pattern as the full population.

* Probability (or difference between probability and unity) was less than machine precision.

Supplementary Table 3. Correlation between present- and previous-trial low-dimensional representations.

	Monkey N			Monkey K		
	Benefit x Previous Benefit	Choice x Previous Choice	Expected Reward x Experienced Reward	Benefit x Previous Benefit	Choice x Previous Choice	Expected Reward x Experienced Reward
r_{ij}	0.12	0.097	0.15	0.021	0.23	0.17
$p(r_{ij} = 0)$	0.35	0.43	0.24	0.70	$1.5 * 10^{-5}$	$1.4 * 10^{-3}$
θ_{ij}	78°	85°	80°	88°	81°	80°
$p(\hat{\theta} = \theta_{ij}, \hat{\theta} > \theta_{ij})$	0.3	0.6	0.3	0.8	0.09	0.05
$p(\hat{\theta} = \theta_{ij}, \hat{\theta} < \theta_{ij})$	0.8	0.4	0.7	0.3	0.9	0.95

Conventions are the same as Supplementary Table 2 except that comparisons are between low-dimensional representations (sRAs) of the same variable with respect to the present vs. previous trials. The correlation r_{ij}, angle θ_{ij}, and associated p-values between present- and previous-trial (*i* and *j*, respectively) sRAs are given here and can be visualized in Supplementary Figure 25. The angles θ_{ij} between previous-trial sRAs and their present-trial counterparts were neither smaller nor larger (i.e., the representations were neither more similar nor more different, respectively) than expected by chance (i.e., $p(\hat{\theta} = \theta_{ij}, \hat{\theta} > \theta_{ij})$ and $p(\hat{\theta} = \theta_{ij}, \hat{\theta} < \theta_{ij})$ were greater than 0.05). Unlike the comparisons between sRAs of different variables within the present (or previous) trial, the null model for the correlation coefficient $\tilde{r}_{ij}$ could not be computed when comparing sRAs for the same variable between the present and previous trials for technical reasons limiting identical execution of the underlying resampling procedure.

Supplementary Table 4. Reliability of and correlation between previous-trial low-dimensional representations.

	Monkey N			Monkey K		
**Within-variable**	Previous Benefit	Previous Choice	Experienced Reward	Previous Benefit	Previous Choice	Experienced Reward
$\tilde{r}_{ii}$	0.63	0.86	0.64	0.57	0.81	0.63
$p(\tilde{r}_{ii} = 0)$	$< 10^{-16}$*	$< 10^{-16}$*	$< 10^{-16}$*	$< 10^{-16}$*	$< 10^{-16}$*	$< 10^{-16}$*
**Between-variable**	Previous Benefit x Previous Choice	Previous Benefit x Experienced Reward	Previous Choice x Experienced Reward	Previous Benefit x Previous Choice	Previous Benefit x Experienced Reward	Previous Choice x Experienced Reward
r_{ij}	0.10	-0.53	-0.55	0.13	-0.73	-0.51
$p(r_{ij} = 0)$	0.42	$3.8 * 10^{-6}$	$9.0 * 10^{-7}$	0.014	$5.0 * 10^{-57}$	$1.9 * 10^{-24}$
mean($\tilde{r}_{ij}$)	0.73	0.63	0.74	0.68	0.60	0.72
$p(\tilde{r}_{ij} = \|r_{ij}\|, \tilde{r}_{ij} > \|r_{ij}\|)$	$< 10^{-16}$*	$< 10^{-16}$*	$< 10^{-16}$*	$< 10^{-16}$*	~1	$< 10^{-16}$*
θ_{ij}	84°	57°	57°	82°	43°	59°
$p(\hat{\theta} = \theta_{ij}, \hat{\theta} > \theta_{ij})$	0.6	0.001	$8 * 10^{-4}$	0.1	$4 * 10^{-18}$	$7 * 10^{-9}$
$p(\hat{\theta} = \theta_{ij}, \hat{\theta} < \theta_{ij})$	0.4	~1*	~1*	0.9	~1*	~1*

Table is identical to Supplementary Table 2 except that low-dimensional representations (sRAs) refer to previous-trial variables of previous benefit, previous choice, and experienced reward. The within-in variable correlation $\tilde{r}_{ii}$ (i.e., sRA reliability) can be visualized in Supplementary Figure 29a-d, whereas the correlations between sRAs (r_{ij}) can be visualized in Supplementary Figures 28 and 29e-h.

In all but one case (PREVIOUS BENEFIT vs. EXPERIENCED REWARD, monkey K only), the previous-trial representations were highly separable (i.e., $p(\tilde{r}_{ij} = |r_{ij}|, \tilde{r}_{ij} > |r_{ij}|) < 0.05$). The angles between PREVIOUS BENEFIT and PREVIOUS CHOICE were not smaller (i.e., the representations were not more similar) than expected by chance, whereas the remaining angles were smaller than chance.

* Probability (or difference between probability and unity) was less than machine precision.

Supplementary Note 1

Solving single-trial problem with trial-average responses

Here, we derive the trial-averaged regression model that is equivalent to the single-trial model in Equation (4). This allows us to write our model compactly as described in Equation (5) in terms of trial-averaged responses.

Let $R_n(r,t)$, as defined in Equation (4), be the firing rate of unit *n* at trial *r* and time bin *t*. Define an experimental condition *c* as the unique combination of *K* task-relevant variables. In the present study, this is the unique combination of benefit, choice and expected reward values. Let $R_n(\forall r_c,t) \in \mathbb{R}^{m_c}$ be the vector containing the firing rates of all trials that belongs to condition *c* and $P_k(\forall r_c) \in \mathbb{R}^{m_c}$ is the corresponding value of the task-relevant variable k, where m_c is the number of trials in condition $c \in \{1,\dots,C\}$. The single-trial model in Equation (4) for trials $r_c \in \{1,\dots,m_c\}$ of condition *c* can thus be written for all trials in vector form as:

$$R_n(\forall r_c,t) = 1_{m_c}\beta_{0,n}(t) + \beta_{1,n}(t)\, P_1(\forall r_c) + \beta_{2,n}(t)\, P_2(\forall r_c) + \beta_{3,n}(t)\, P_3(\forall r_c) + \varepsilon$$

where 1_{m_c} is a vector of ones of length m_c that repeats $\beta_{0,n}(t)$ as many times as trial count m_c.

Since the value of $P_k(r_c)$ is the same for all trials in experimental condition *c*, the above can be written as:

$$R_n(\forall r_c,t) = 1_{m_c}\beta_{0,n}(t) + 1_{m_c}\beta_{1,n}(t)\, P_1(c) + 1_{m_c}\beta_{2,n}(t)\, P_2(c) + 1_{m_c}\beta_{3,n}(t)\, P_3(c) + \varepsilon$$

where $P_k(c)$ is the task-relevant variable for condition *c*, which can be rewritten more compactly as:

$$R_n(\forall r_c,t) = 1_{m_c}P(c)^\top B(t) + \varepsilon$$

where $B(t) = \left[\beta_{0,n}(t), \beta_{1,n}(t), \beta_{2,n}(t), \beta_{3,n}(t)\right]^\top$ and $P(c) = [1, P_1(c), P_2(c), P_3(c)]^\top$.

To solve for all trials and conditions, we solve the following linear regression problem:

$$\hat{B} = argmin_{B(t)\in\mathbb{R}^{K+1}} \|R_n(t) - \, P^\top B(t)\|$$

where $R_n(t) = \begin{bmatrix} R_n(\forall r_1, t) \\ \vdots \\ R_n(\forall r_C, t) \end{bmatrix} \in \mathbb{R}^{\sum_{c=1}^{C} m_c}$ is the vector of all firing rates for unit *n* at a time bin *t* across all trials and conditions, and $P = \begin{bmatrix} 1_{m_1} P(1)^\top \\ \vdots \\ 1_{m_C} P(C)^\top \end{bmatrix} \in \mathbb{R}^{(\sum_{c=1}^{C} m_c) \times (K+1)}$ is the matrix with all task-relevant variables.

The solution of this problem is the least-square solution given by:

$$\hat{B}(t) = (P^\top P)^{-1} P^T R_n(t)$$

$$= \left(\begin{bmatrix} P(1)\, 1_{m_1}^\top & \cdots & P(C)\, 1_{m_C}^\top \end{bmatrix} \begin{bmatrix} 1_{m_1} P(1)^\top \\ \vdots \\ 1_{m_C} P(C)^\top \end{bmatrix} \right)^{-1} \begin{bmatrix} P(1)\, 1_{m_1}^\top & \cdots & P(C)\, 1_{m_C}^\top \end{bmatrix} \begin{bmatrix} R_n(\forall r_1, t) \\ \vdots \\ R_n(\forall r_C, t) \end{bmatrix}$$

$$= \left(\left[P(1)\, 1_{m_1}^\top 1_{m_1} P(1)^\top \cdots P(C)\, 1_{m_C}^\top 1_{m_C} P(C)^\top \right] \right)^{-1} \left[P(1)\, 1_{m_1}^\top R_n(\forall r_1, t) \cdots P(C)\, 1_{m_C}^\top R_n(\forall r_C, t) \right]$$

$$= \left(\left[m_1 P(1) P(1)^\top \quad \cdots \quad m_C P(C) P(C)^\top \right] \right)^{-1} \left[m_1 P(1) \bar{R}_n(1, t) \quad \cdots \quad m_C P(C) \bar{R}_n(C, t) \right]$$

where $\bar{R}_n(c, t) = \frac{1_{m_C}^\top R_n(\forall r_c, t)}{m_c} = \frac{1}{m_c} \sum_{r_c=1}^{m_c} R_n(r_c, t)$ is the trial-averaged firing rate of condition *c*.

The solution then reduces to:

$$\hat{B}(t) = \left(\left[P(1) \cdots P(C) \right] \begin{bmatrix} m_1 & & \\ & \ddots & \\ & & m_c \end{bmatrix} \begin{bmatrix} P(1)^\top \\ \vdots \\ P(C)^\top \end{bmatrix} \right)^{-1} \left[P(1) \cdots P(C) \right] \begin{bmatrix} m_1 & & \\ & \ddots & \\ & & m_c \end{bmatrix} \begin{bmatrix} \bar{R}_n(1, t) \\ \vdots \\ \bar{R}_n(C, t) \end{bmatrix}$$

$$= (\bar{P}^\top M \bar{P})^{-1} \bar{P}^\top M \bar{R}_n(t)$$

where $\bar{P} = \begin{bmatrix} P(1)^\top \\ \vdots \\ P(C)^\top \end{bmatrix}$, $M = \begin{bmatrix} m_1 & & \\ & \ddots & \\ & & m_c \end{bmatrix}$, and $\bar{R}_n(t) = \begin{bmatrix} \bar{R}_n(1, t) \\ \vdots \\ \bar{R}_n(C, t) \end{bmatrix}$.

Writing the trial count diagonal matrix as a product of square roots yields:

$$\hat{B}(t) = \left(\bar{P}^\top \sqrt{M} \sqrt{M} \bar{P} \right)^{-1} \bar{P}^\top \sqrt{M} \sqrt{M} \bar{R}_n(t) = \left(\left(\sqrt{M} \bar{P} \right)^\top \left(\sqrt{M} \bar{P} \right) \right)^{-1} \left(\sqrt{M} \bar{P} \right)^\top \left(\sqrt{M} \bar{R}_n(t) \right)$$

The expression above is the least-square solution to the following problem:

$$\hat{B}(t) = argmin_{B(t)\in\mathbb{R}^{K+1}} \left\| \sqrt{M}\bar{R}_n(t) - \sqrt{M}\bar{P}B(t) \right\|$$

The above problem only includes trial-averaged firing rates. Thus, we show equivalency between the model using single-trial observations (expressed in terms of R_n) and the model using trial-averaged observations (expressed in terms of $\bar{R}_n$), weighted by trial-count (given in M):

$$min_{B(t)\in\mathbb{R}^{K+1}} \| R_n(t) - P^{\top}B(t) \| \rightarrow min_{B(t)\in\mathbb{R}^{K+1}} \left\| \sqrt{M} \left(\bar{R}_n(t) - \sqrt{M}\bar{P}B(t) \right) \right\|$$

Supplementary References

1. Harvey, C. D., Coen, P. & Tank, D. W. Choice-specific sequences in parietal cortex during a virtual-navigation decision task. *Nature* **484**, 62–8 (2012).
2. Driscoll, L. N., Pettit, N. L., Minderer, M., Chettih, S. N. & Harvey, C. D. Dynamic Reorganization of Neuronal Activity Patterns in Parietal Cortex. *Cell* **170**, 986-999.e16 (2017).
3. Runyan, C. A., Piasini, E., Panzeri, S. & Harvey, C. D. Distinct timescales of population coding across cortex. *Nature* **548**, 92–96 (2017).
4. Akhlaghpour, H. *et al.* Dissociated sequential activity and stimulus encoding in the dorsomedial striatum during spatial working memory. *Elife* **5**, (2016).
5. Morcos, A. S. & Harvey, C. D. History-dependent variability in population dynamics during evidence accumulation in cortex. *Nat. Neurosci.* **19**, 1672–1681 (2016).
6. Xie, J. & Padoa-Schioppa, C. Neuronal remapping and circuit persistence in economic decisions. *Nat. Neurosci.* **19**, 855–61 (2016).
7. Rigotti, M. *et al.* The importance of mixed selectivity in complex cognitive tasks. *Nature* **497**, 585–590 (2013).
8. Raposo, D., Kaufman, M. T. & Churchland, A. K. A category-free neural population supports evolving demands during decision-making. *Nat. Neurosci.* **17**, 1784–1792 (2014).
9. Fusi, S., Miller, E. K. & Rigotti, M. Why neurons mix: High dimensionality for higher cognition. *Curr. Opin. Neurobiol.* **37**, 66–74 (2016).
10. Britten, K. H., Newsome, W. T., Shadlen, M. N., Celebrini, S. & Movshon, J. A. A relationship between behavioral choice and the visual responses of neurons in macaque MT. *Vis. Neurosci.* **13**, 87–100 (1996).
11. Luna, R., Hernández, A., Brody, C. D. & Romo, R. Neural codes for perceptual discrimination in primary somatosensory cortex. *Nat. Neurosci.* **8**, 1210–9 (2005).
12. Padoa-Schioppa, C. & Assad, J. A. Neurons in the orbitofrontal cortex encode economic value. *Nature* **441**, 223–6 (2006).
13. Strait, C. E., Blanchard, T. C. & Hayden, B. Y. Reward value comparison via mutual inhibition in ventromedial prefrontal cortex. *Neuron* **82**, 1357–1366 (2014).
14. Green, D. & Swets, J. *Signal detection theory and psychophysics*. (Wiley, 1966).
15. Law, C.-T. & Gold, J. I. Neural correlates of perceptual learning in a sensory-motor, but not a sensory, cortical area. *Nat. Neurosci.* **11**, 505–13 (2008).
