# Supplementary material for: Value and choice as separable and stable representations in orbitofrontal cortex
Source: Nat Commun. 2020 Jul 10;11:3466. doi: 10.1038/s41467-020-17058-y (PMC7351792; doi:10.1038/s41467-020-17058-y)
Supplement: Supplementary file 3 — Reporting Summary [file 41467_2020_17058_MOESM3_ESM.pdf]

nature research

Corresponding author(s): Daniel L. Kimmel

Last updated by author(s): May 25, 2020

Reporting Summary

Nature Research wishes to improve the reproducibility of the work that we publish. This form provides structure for consistency and transparency in reporting. For further information on Nature Research policies, see our Editorial Policies and the Editorial Policy Checklist.

Statistics

For all statistical analyses, confirm that the following items are present in the figure legend, table legend, main text, or Methods section.

n/a	Confirmed	
☐	☒	The exact sample size (n) for each experimental group/condition, given as a discrete number and unit of measurement
☐	☒	A statement on whether measurements were taken from distinct samples or whether the same sample was measured repeatedly
☐	☒	The statistical test(s) used AND whether they are one- or two-sided<br>*Only common tests should be described solely by name; describe more complex techniques in the Methods section.*
☐	☒	A description of all covariates tested
☐	☒	A description of any assumptions or corrections, such as tests of normality and adjustment for multiple comparisons
☐	☒	A full description of the statistical parameters including central tendency (e.g. means) or other basic estimates (e.g. regression coefficient) AND variation (e.g. standard deviation) or associated estimates of uncertainty (e.g. confidence intervals)
☐	☒	For null hypothesis testing, the test statistic (e.g. F, t, r) with confidence intervals, effect sizes, degrees of freedom and P value noted<br>*Give P values as exact values whenever suitable.*
☒	☐	For Bayesian analysis, information on the choice of priors and Markov chain Monte Carlo settings
☒	☐	For hierarchical and complex designs, identification of the appropriate level for tests and full reporting of outcomes
☐	☒	Estimates of effect sizes (e.g. Cohen's d, Pearson's r), indicating how they were calculated

Our web collection on statistics for biologists contains articles on many of the points above.

Software and code

Policy information about availability of computer code

Data collection	Behavioral control and stimulus presentation were managed by Apple Macintosh G5-based computers (Cupertino, CA) running Expo software (v. 1.5.5) written by Peter Lennie (University of Rochester, NY) with modifications by Julian Brown (Stanford University, CA). Behavioral and stimulus event data were acquired by the Plexon MAP System (Dallas, TX), while digital eye position samples were recorded natively on the EyeLink system.
Data analysis	Offline spike sorting was performed manually using Plexon Offline Sorter (v. 2.8). All analyses were performed with custom scripts written in MATLAB (Mathworks, Natick, MA) version R2019a (v. 9.6).

For manuscripts utilizing custom algorithms or software that are central to the research but not yet described in published literature, software must be made available to editors and reviewers. We strongly encourage code deposition in a community repository (e.g. GitHub). See the Nature Research guidelines for submitting code & software for further information.

Data

Policy information about availability of data

All manuscripts must include a data availability statement. This statement should provide the following information, where applicable:

- Accession codes, unique identifiers, or web links for publicly available datasets
- A list of figures that have associated raw data
- A description of any restrictions on data availability

The source data underlying Figures 1b-c, 2a-e, 3a-d, 4a-f, 5a-f, and 6a-h are available for download at https://github.com/danielkimmel/Kimmel_NatComm_2020.

Field-specific reporting

Please select the one below that is the best fit for your research. If you are not sure, read the appropriate sections before making your selection.

☒ Life sciences ☐ Behavioural & social sciences ☐ Ecological, evolutionary & environmental sciences

For a reference copy of the document with all sections, see nature.com/documents/nr-reporting-summary-flat.pdf

Life sciences study design

All studies must disclose on these points even when the disclosure is negative.

Sample size	As is common in primate electrophysiology, two adult male macaque monkeys, N and K, served as subjects in this study so as to ensure reproducibility of findings beyond one subject (e.g., Padoa-Schioppa & Assad, Nature, 2006; Elsayed et al., Nat Comm, 2016; Munuera et al., Nat Neuro, 2018; Wutz et al., Neuron, 2018; Sarafyazd & Jazayeri, Science, 2019). In our study, each animal participated in multiple experimental sessions, which was determined by the number required to adequately sample the anatomical space of OFC (specifically Brodmann areas 11 and 13).
Data exclusions	As described in Methods, we applied the following exclusion criteria, which were pre-established. We excluded units that were recorded for fewer than 60 trials total (44 and 33 units, monkey N and K, respectively), had fewer than 5 trials in any of the included conditions (i.e., up to 10 unique combinations of offer size and choice; 71 and 133 units), or for which the accompanying behavior was grossly aberrant (monkey K only: 1 session with accept rate of ~55% for 0-reward offers and 1 session with ~10% accept rate for all offers). When a unit was not present during a trial (i.e., mean spike rate across trial < 0.1 Hz) for 5 or more consecutive trials, we excluded those trials for that unit; this excluded both units with very low firing rates, as well as trials during which the unit may have been lost to recording. Finally, because our analysis would ultimately normalize a unit's firing rate by its variability, we excluded 23 units (monkey K only) with extremely low variability (s.d. < 0.5 Hz) as measured across conditions and time bins so that imprecision in estimating variability (to which low-variability units were particularly susceptible) would not result in spurious over-weighting of these units.<br>The following exclusion criterion depended on the animals' behavioral choices, and thus was applied after examining the data, but was not informed by its impact on any of the measures of interest. Specifically, because our analysis required that all included conditions were represented by all units, we excluded conditions for which less than 40% of units met the trial count threshold (i.e., 60 trials), including "0-reward, accept" for both animals and additionally "8-reward, reject" for monkey K. This criterion was chosen to jointly maximize the number of included units and conditions.
Replication	Results were replicated independently on complete population datasets from two animals. All attempts at replication of the reported findings were successful. Within a given analysis, statistical methods including bootstrapping and cross-validation were used to mitigate false positives and over-fitting.
Randomization	In a within subject design, a given subject performed a series of cost-benefit decision trials where the value of each offer was randomized from trial to trial.
Blinding	As above, randomization occurred at the trial-level (not subject-level). Experimenter and subject were blinded as to the identity of each trial until the trial occurred.

Reporting for specific materials, systems and methods

We require information from authors about some types of materials, experimental systems and methods used in many studies. Here, indicate whether each material, system or method listed is relevant to your study. If you are not sure if a list item applies to your research, read the appropriate section before selecting a response.

Materials & experimental systems

n/a	Involved in the study	
☒	☐	Antibodies
☒	☐	Eukaryotic cell lines
☒	☐	Palaeontology and archaeology
☐	☒	Animals and other organisms
☒	☐	Human research participants
☒	☐	Clinical data
☒	☐	Dual use research of concern

Methods

n/a	Involved in the study	
☒	☐	ChIP-seq
☒	☐	Flow cytometry
☒	☐	MRI-based neuroimaging

Animals and other organisms

Policy information about studies involving animals; ARRIVE guidelines recommended for reporting animal research

Laboratory animals	Two adult male macaque monkeys, N (Macacca fascicularis, age 10 y) and K (M. mulatta, age 7 y), served as subjects in this study.

Wild animals	No wild animals were used in the study.
Field-collected samples	No field-collected samples were used in the study.
Ethics oversight	All surgical, behavioral, and animal care procedures complied with National Institutes of Health guidelines and were approved by the Stanford Institutional Animal Care and Use Committee.

Note that full information on the approval of the study protocol must also be provided in the manuscript.
